# Fractalkine enhances oligodendrocyte regeneration and remyelination in a demyelination mouse model

**DOI:** 10.1016/j.stemcr.2022.12.001

**Published:** 2023-01-05

**Authors:** Monique M.A. de Almeida, Adrianne E.S. Watson, Sana Bibi, Nicole L. Dittmann, Kara Goodkey, Pedram Sharafodinzadeh, Danny Galleguillos, Maryam Nakhaei-Nejad, Jayasankar Kosaraju, Noam Steinberg, Beatrix S. Wang, Tim Footz, Fabrizio Giuliani, Jing Wang, Simonetta Sipione, Julia M. Edgar, Anastassia Voronova

**Affiliations:** 1 Department of Medical Genetics, Faculty of Medicine & Dentistry, University of Alberta, Edmonton, AB T6G 2H7, Canada; 2 Women and Children’s Health Research Institute, University of Alberta, 5-083 Edmonton Clinic Health Academy, 11405 87 Avenue NW, Edmonton, AB T6G 1C9, Canada; 3 Neuroscience and Mental Health Institute, Faculty of Medicine & Dentistry, Edmonton, AB T6G 2E1, Canada; 4 Department of Pharmacology, Faculty of Medicine & Dentistry, Edmonton, AB T6G 2H7, Canada; 5 Department of Medicine, Faculty of Medicine & Dentistry, Edmonton, AB T6G 2H7, Canada; 6 Regenerative Medicine Program, Ottawa Hospital Research Institute, Ottawa, ON K1H 8L6, Canada; 7 Department of Cellular and Molecular Medicine, Faculty of Medicine, University of Ottawa Brain and Mind Research Institute, Ottawa, ON K1H 8M5, Canada; 8 School of Infection and Immunity, College of Medical Veterinary and Life Sciences, University of Glasgow, Glasgow G12 8TA, UK; 9 Department of Cell Biology, Faculty of Medicine & Dentistry, Edmonton, AB T6G 2H7, Canada; 10 Multiple Sclerosis Centre and Department of Cell Biology, Faculty of Medicine & Dentistry, Edmonton, AB T6G 2H7, Canada

**Keywords:** OPC, NG2, myelination, differentiation, neuron-glia, chemokine, regeneration, CX3CR1, CX3CL1, multiple sclerosis

## Abstract

Demyelinating disorders of the central nervous system (CNS) occur when myelin and oligodendrocytes are damaged or lost. Remyelination and regeneration of oligodendrocytes can be achieved from endogenous oligodendrocyte precursor cells (OPCs) that reside in the adult CNS tissue. Using a cuprizone mouse model of demyelination, we show that infusion of fractalkine (CX3CL1) into the demyelinated murine brain increases *de novo* oligodendrocyte formation and enhances remyelination in the corpus callosum and cortical gray matter. This is achieved by increased OPC proliferation in the cortical gray matter as well as OPC differentiation and attenuation of microglia/macrophage activation both in corpus callosum and cortical gray matter. Finally, we show that activated OPCs and microglia/macrophages express fractalkine receptor CX3CR1 *in vivo*, and that in OPC-microglia co-cultures fractalkine increases *in vitro* oligodendrocyte differentiation by modulating both OPC and microglia biology. Our results demonstrate a novel pro-regenerative role of fractalkine in a demyelinating mouse model.

## Highlights

• CX3CR1 is expressed in mouse and human microglia and OPCs in demyelinated brain
• Intracerebral fractalkine infusion after demyelinating injury enhances remyelination
• Fractalkine enhances oligodendrogenesis by directly regulating microglia and OPCs

## Graphical abstract

**Figure:**
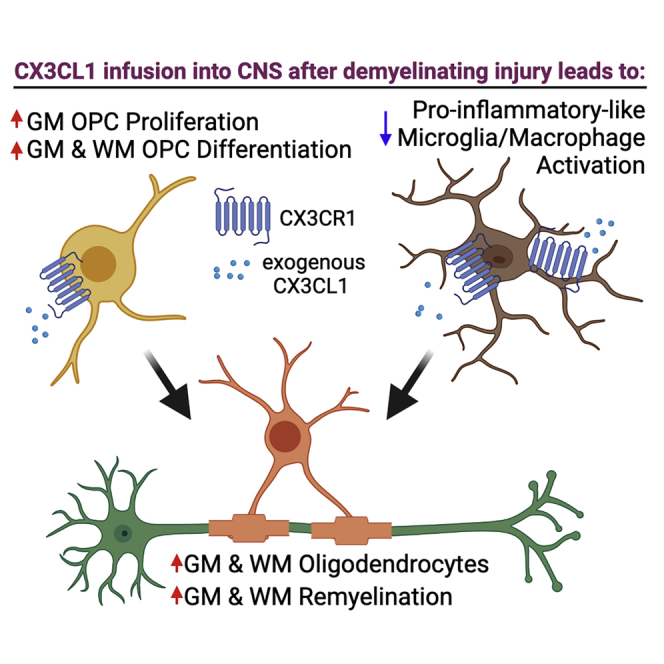

## Teaser

Here, authors demonstrate administration of the chemokine fractalkine (CX3CL1) into the brain after demyelinating injury enhances oligodendrocyte and myelin regeneration by modulating OPC proliferation and differentiation as well as microglia/macrophage activation *in vivo*. They further show OPCs and microglia express fractalkine receptor (CX3CR1) in the demyelinated CNS and that fractalkine increases *in vitro* oligodendrogenesis by modulating both OPC and microglia biology.

## Introduction

Multiple sclerosis (MS) is an autoimmune demyelinating disorder where myelin and the myelin-producing cells, oligodendrocytes, are damaged or lost in the central nervous system (CNS). There are currently no approved treatments that promote CNS remyelination, which are hypothesized to halt disease progression or enable neurological repair (Göttle et al., 2019). Remyelination can be achieved through *de novo* oligodendrocyte formation from adult parenchymal oligodendrocyte precursor cells (OPCs). When demyelination occurs, OPCs proliferate and migrate to the lesion site, where they differentiate into oligodendrocytes that remyelinate the lesion (McMurran et al., 2016). However, in people with MS, this process is highly inefficient (Yeung et al., 2019).

OPC fates, such as survival, proliferation, and differentiation, are important for efficient oligodendrocyte generation and are regulated by neighboring cells, such as microglia (Miron et al., 2013; Nemes-Baran et al., 2020; Sherafat et al., 2021b). While microglia can adopt a wide array of activation states, in general microglia can be either detrimental to regeneration through the release of pro-inflammatory cytokines, or beneficial by clearing the myelin debris and/or secreting anti-inflammatory and pro-oligodendrogenic proteins (Heß et al., 2020; Miron et al., 2013; Sherafat et al., 2021b).

OPCs are also regulated by chemokines, like neuronally secreted fractalkine (CX3CL1/FKN) (Watson et al., 2020). FKN signals through its sole receptor, CX3CR1, which is expressed at high levels in microglia and at lower levels in OPCs (Voronova et al., 2017; Watson et al., 2021). While the role of FKN signaling in adult parenchymal OPCs is unknown, we have shown that mice with constitutive Cx3cr1 KO (knockout), or with reduced levels of Cx3cr1 in cortical progenitors, have decreased developmental oligodendrocyte genesis (Voronova et al., 2017). Moreover, demyelinated Cx3cr1 KO mice and/or mice that express a human MS-associated *CX3CR1* variant display poor remyelination, impaired microglial phagocytosis, and decreased OPC migration and proliferation (Cardona et al., 2018; Huang et al., 2006; Lampron et al., 2015; Mendiola et al., 2022). These reports suggest that FKN-CX3CR1 signaling is a critical regulator of oligodendrogenesis and myelination, and that activation of this pathway may be beneficial for remyelination. Whether exogenous FKN can increase oligodendrocyte and myelin regeneration from adult parenchymal OPCs remains to be addressed.

We show exogenous FKN infusion into the brain after cuprizone-induced demyelinating injury increases *de novo* oligodendrocyte formation and remyelination from parenchymal OPCs and attenuates microglia activation *in vivo*. In co-culture conditions, both OPCs and microglia need to be stimulated with FKN to elicit an increase in oligodendrocyte differentiation, suggesting that FKN regulates both OPC and microglia biology for a pro-oligodendrogenic response.

## Results

### *Cx3cr1* is expressed in OPCs and microglia in demyelinated CNS

**Figure 1:**
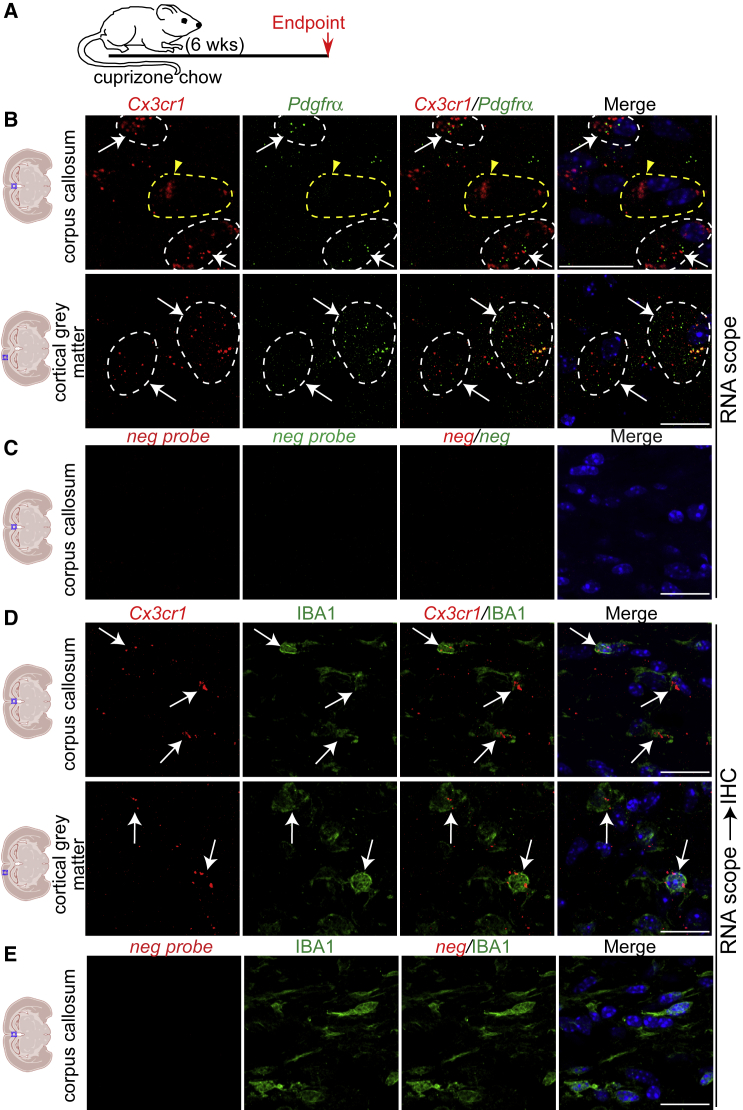
OPCs and microglia express *Cx3cr1* mRNA in demyelinated brain (A) Ten-week-old C57BL/6J mice were demyelinated with cuprizone chow for 6 weeks. (B and D) RNA scope analysis of demyelinated brains for *Cx3cr1* (red, B, D) and *Pdgfrα* (green, B) mRNAs or immunostained for IBA1 (green, D). White arrows and dashed lines indicate *Cxc3r1*+marker+ cells. Yellow arrowhead and dashed line indicate a *Cx3cr1*+*marker*- cell. (C and E) RNA Scope analysis of midline CC from demyelinated brains with negative probe. Nuclei are visualized with DAPI (blue). Scale bars, 20 μm. n = 2 for each group. See also Figure S1.

Analysis of a single nuclei RNA sequencing dataset from MS patient brain white matter lesions showed *CX3CR1* is expressed in committed OPCs (cOPCs), immune oligodendrocytes (ImOLGS), and in microglia/macrophages (Jäkel et al., 2019) (Figure S1A). To probe *Cx3cr1* mRNA expression in the murine demyelinated brain, we subjected 10-week-old wild-type (WT) mice to a 6-week cuprizone chow treatment, which leads to corpus callosum (CC) and cortical gray matter (GM) demyelination (Baxi et al., 2017) (Figures 1A, S1B and S1C). RNA scope showed *Cx3cr1* mRNA was detected in *Pdgfrα*+ OPCs and IBA1+ microglia/macrophages in demyelinated midline CC and cortical GM (Figures 1B–1E). We corroborated these results in CC focally demyelinated with lysolethicin (Figures S1D and S1E). Thus, OPCs and microglia/macrophages express *Cx3cr1* in the demyelinated CNS and are poised to respond to FKN.

### Exogenous FKN increases *de novo* oligodendrocyte genesis in remyelinating CC and cortical GM

**Figure 2:**
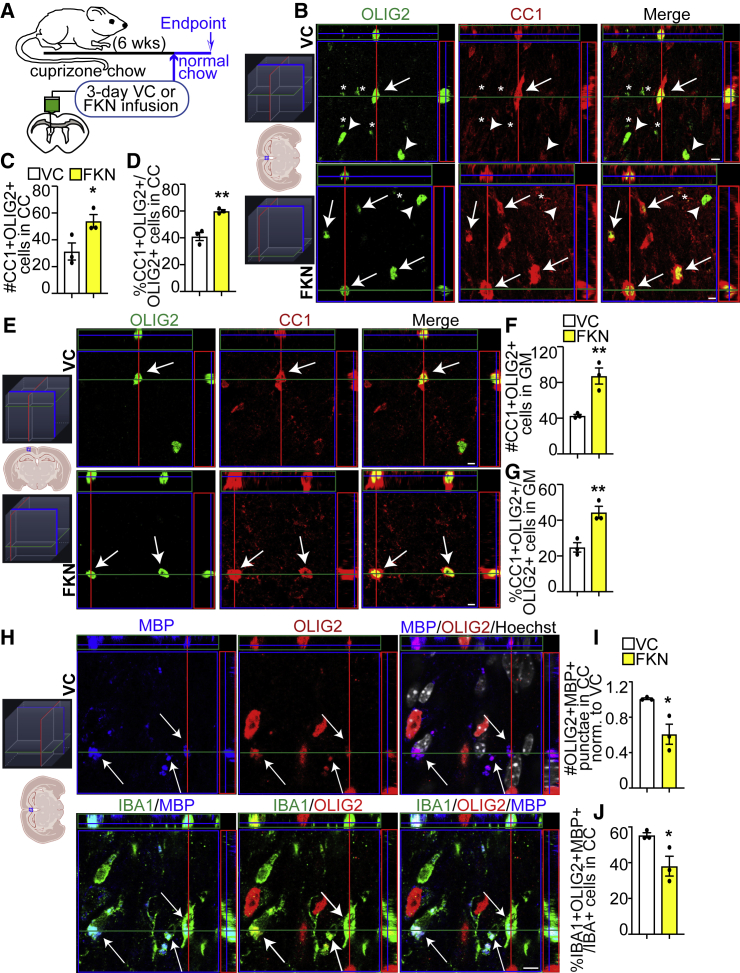
FKN increases oligodendrocyte density after demyelinating injury (A) Ten-week-old C57BL/6J mice were fed cuprizone chow for 6 weeks. VC/FKN was infused into the lateral ventricle for 3 days via osmotic mini-pump after cuprizone cessation and mice were returned to normal chow. (B and E) Representative orthogonal slices of z-stack images from midline CC (B) or cortical GM (E) immunostained for CC1 (red) and OLIG2 (green) from VC- (top) and FKN- (bottom) infused mice (see 3D schematic for orientation). Arrows indicate CC1+OLIG2+, arrowheads CC1-OLIG2+ cells, and asterisks OLIG2+ punctate staining (please see Figure 2H for more details). (C and D) Quantification of (B). (F and G) Quantification of (E). (H) Representative orthogonal slices of z-stack images from midline CC immunostained for MBP (blue), OLIG2 (red), and IBA1 (green) from VC-infused mice. Hoechst (white) indicates nuclei (see 3D schematic for orientation). Arrows indicate MBP+OLIG2+Hoechst- cellular material engulfed by IBA1+ cells. (I and J) Quantification of (H). Scale bars, 5 μm. Error bars represent SEM. Data were analyzed using unpaired t test, ^∗^p < 0.05, ^∗∗^p < 0.01, n = 3 mice per group. See also Figure S2.

To test whether FKN increases oligodendrocyte production after a demyelinating injury, cuprizone-treated WT mice were subjected to intracerebroventricular (ICV) surgery and 3-day VC/FKN infusion. After surgery, mice were returned to normal chow to allow remyelination (Figure 2A). We focused our analysis on medial brain areas, as they are remyelinated by parenchymal OPCs (Xing et al., 2014). At 3 days of infusion, the number and proportion of cells that expressed both OLIG2 (marker of oligodendroglial cells [OPCs and oligodendrocytes]) and CC1 (marker of mature oligodendrocytes), was increased ∼1.46- to 2-fold in the CC and cortical GM in FKN-infused mice when compared with VC (Figures 2B–2G, and S2).

Intriguingly, we detected punctate OLIG2 signal specifically in the CC of infused animals that were not associated with Hoechst staining. These OLIG2 puncta showed co-localization with myelin basic protein (MBP) and were engulfed by IBA1+ microglia/macrophages (Figure 2H), suggesting that these puncta represent degenerated oligodendrocyte material. Notably, both the number of OLIG2+MBP+ puncta, and the proportion of IBA1+ cells engulfing OLIG2+MBP+ puncta were decreased in FKN-infused animals when compared with VC (Figures 2I and 2J). These data support and expand prior reports, which demonstrate tissue-protective properties of FKN (Cipriani et al., 2011; Morganti et al., 2012; O'Sullivan and Dev, 2017; Pabon et al., 2011); and altered phagocytosis by microglia/macrophages in Cx3cr1 KO mice (Lampron et al., 2015).

**Figure 3:**
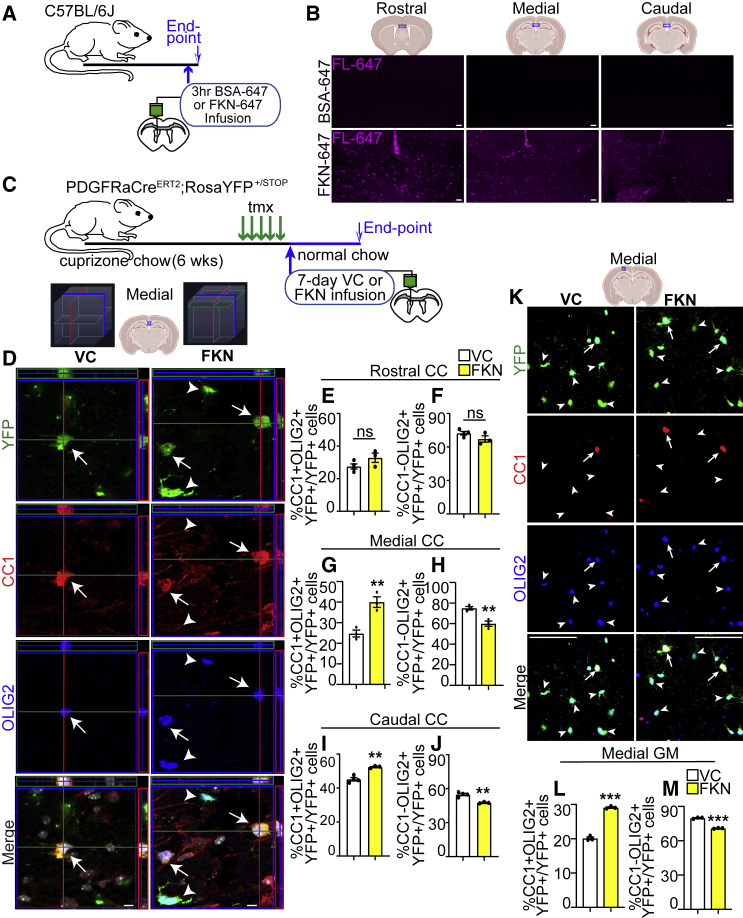
FKN increases *de novo* oligodendrocyte genesis after demyelinating injury (A) Adult WT mice were infused with FKN-647 or BSA-647 into the lateral ventricle and euthanized 3 h later. (B) Representative images from BSA-647 (top) and FKN-647 (bottom)-infused brains imaged using fluorescence from far-red (FL-647) channel. (C) Ten-week-old PDGFRαCre^ERT2^;RosaYFP ^STOP/+^ were fed cuprizone chow for 6 weeks. Tamoxifen was injected in the last week of cuprizone treatment. VC/FKN was infused into the lateral ventricle after cuprizone cessation for 7 days and mice were returned to normal chow. (D) Representative orthogonal slices of z-stack images of midline CC immunostained for YFP (green), CC1 (red), and OLIG2 (blue) from VC- (left) and FKN- (right) infused mice (see 3D schematic for orientation). Arrows indicate YFP+CC1+OLIG2+ and arrowheads YFP+CC1-OLIG2+ cells. (E–J) Quantification of (D) in rostral (E and F), medial (G and H) and caudal (I and J) CC. (K) Representative images of medial cortical GM immunostained for YFP (green), CC1 (red), and OLIG2 (blue). Arrows indicate YFP+CC1+OLIG2+ and arrowheads YFP+CC1-OLIG2+ cells. (L and M) Quantification of (K). Scale bars, 50 μm in (B), 5 μm in (D), 25 μm in (K). Error bars represent SEM. Data were analyzed using unpaired t test, ^∗^p < 0.05, ^∗∗^p < 0.01, n = 3 mice per group from at least two independent litters. See also Figure S3.

We then asked if the effect of FKN on oligodendrocyte density increase is preserved (1) in a rostro-caudal gradient, (2) at a later time point, and whether (3) it is due to OPC differentiation into *de novo* oligodendrocytes. First, we showed injection of FKN directly conjugated to fluorophore Alexa 647 (FKN-647) into the lateral ventricle led to the diffusion into the rostral, medial, and caudal regions of the brain (Figures 3A and 3B). We then subjected OPC lineage tracing mice (PDGFRαCre^ERT2^;RosaYFP^+/STOP^) to a 6-week cuprizone demyelination. Tamoxifen administration in these mice leads to efficient OPC labeling, where 100% of PDGFRα+OLIG2+ cells are YFP+, and 92.9% ± 2.3% of YFP+ cells are PDGFRα+OLIG2+ (Figures S3A–S3C). In the last week of cuprizone treatment, mice were injected with tamoxifen for 5 days to induce recombination in the activated PDGFRα+ OPCs. Seventy-two hours later, mice were subjected to VC/FKN ICV infusion, followed by a 7-day recovery phase (Figure 3C) to allow cortical GM repopulation by *de novo* oligodendrocytes, which occurs at a slower pace compared with CC (Baxi et al., 2017). Exogenous FKN caused an ∼1.45- to 1.6-fold increase in the medial cortical GM and CC in the proportion of newborn CC1+OLIG2+YFP+ mature oligodendrocytes (Figures 3D, 3G, 3K, and 3L), but did not alter the total number of YFP+ cells (Figures S3E and S3G). Concomitantly, FKN infusion led to a decrease in the proportion of CC1-OLIG2+YFP+ cells, which represent a mixture of OPCs and immature oligodendrocytes, in the medial cortical GM and CC (Figures 3H and 3M). Similarly, FKN also caused an increase in CC1+OLIG2+YFP+ oligodendrocytes and a decrease in CC1-OLIG2+YFP+ cells in the caudal CC (Figures 3I and 3J). However, there were no changes in the YFP+ oligodendroglial cells in the rostral CC (Figures 3E and 3F). This is likely because the rostral brain is preferentially remyelinated by SVZ NPCs, rather than parenchymal PDGFRα+ OPCs (Jablonska et al., 2010; Kang et al., 2010; Nait-Oumesmar et al., 1999; Xing et al., 2014).

Thus, FKN infusion leads to a persistent increase in the number of *de novo* oligodendrocytes from activated parenchymal OPCs. As this effect was most prominent in the medial brain regions, we focused our remaining analyses on this area.

### Exogenous FKN increases OPC proliferation in remyelinating cortical GM, but not CC

**Figure 4:**
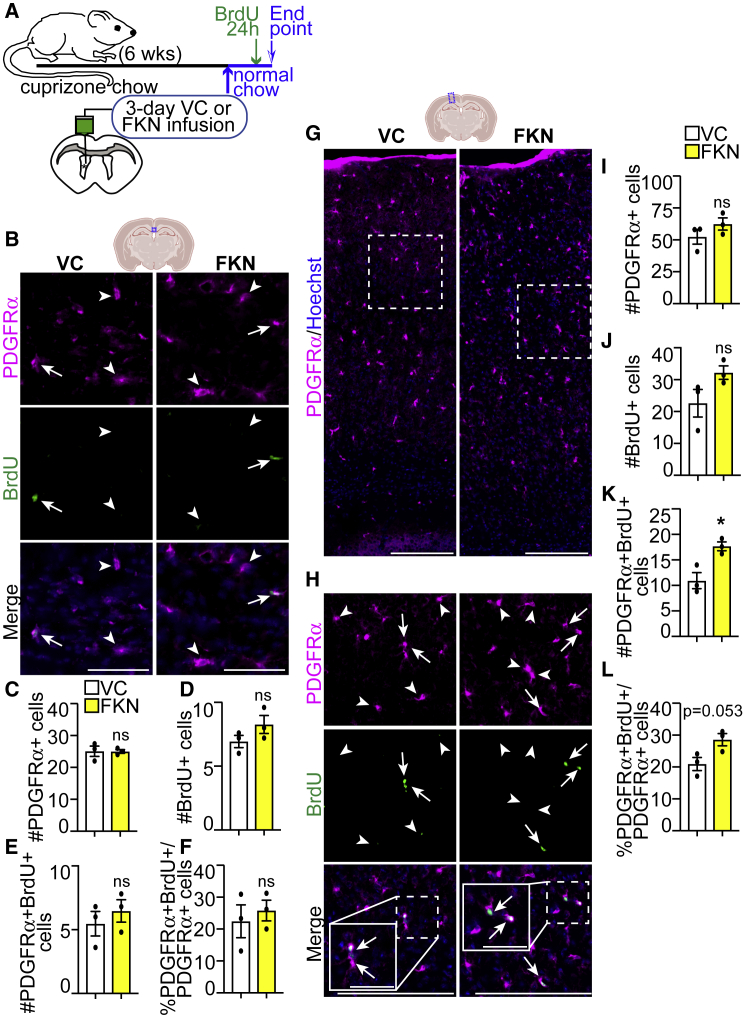
FKN increases OPC proliferation specifically in the remyelinating cortical GM (A) Ten-week-old C57BL/6J mice were fed cuprizone chow for 6 weeks. VC/FKN was infused into the lateral ventricle for 3 days after cuprizone cessation, and mice were returned to normal chow. BrdU was injected 24 h before euthanasia. (B) Representative images of midline CC immunostained for PDGFRα (magenta), BrdU (green), and counterstained with Hoechst (blue) from VC- and FKN-infused mice. (C–F) Quantification of (B). (G) Representative images of cortical GM immunostained for PDGFRα (magenta) and counterstained with Hoechst (blue) from VC- and FKN-infused mice. (H) High-magnification images from (G) immunostained for PDGFRα (magenta), BrdU (green), and counterstained with Hoechst (blue). Hatched insets are shown at higher magnification in solid boxes in merge panels. Arrows indicate PDGFRα+BrdU+ and arrowheads PDGFRα+BrdU− cells. (I–L) Quantification of (G and H). Scale bars, 50 μm in (B), 200 μm in (G and H), 50 μm in (H) insets. Error bars represent SEM. Data were analyzed using unpaired t test, ^∗^p < 0.05, ns = not significant. n = 3 mice per group.

To determine whether an increase in oligodendrocyte formation was due to changes in OPC proliferation, we subjected cuprizone-demyelinated WT mice to VC/FKN ICV and returned to normal chow for 3 days. 24 h before euthanasia, BrdU (Bromodeoxyuridine) was injected to label proliferating cells (Figure 4A). FKN infusion did not lead to changes in the number of PDGFRα+ OPCs or BrdU+ proliferating cells in the midline CC or cortical GM (Figures 4B–4D and 4G–4J). However, exogenous FKN led to an ∼1.6-fold increase in the number of PDGFRα+BrdU+ proliferating OPCs and a trending increase in the proliferative index of OPCs in the cortical GM, but not the CC (Figures 4E, 4F, 4K, and 4L).

### Exogenous FKN attenuates microglia/macrophage, but not astrocyte activation

**Figure 5:**
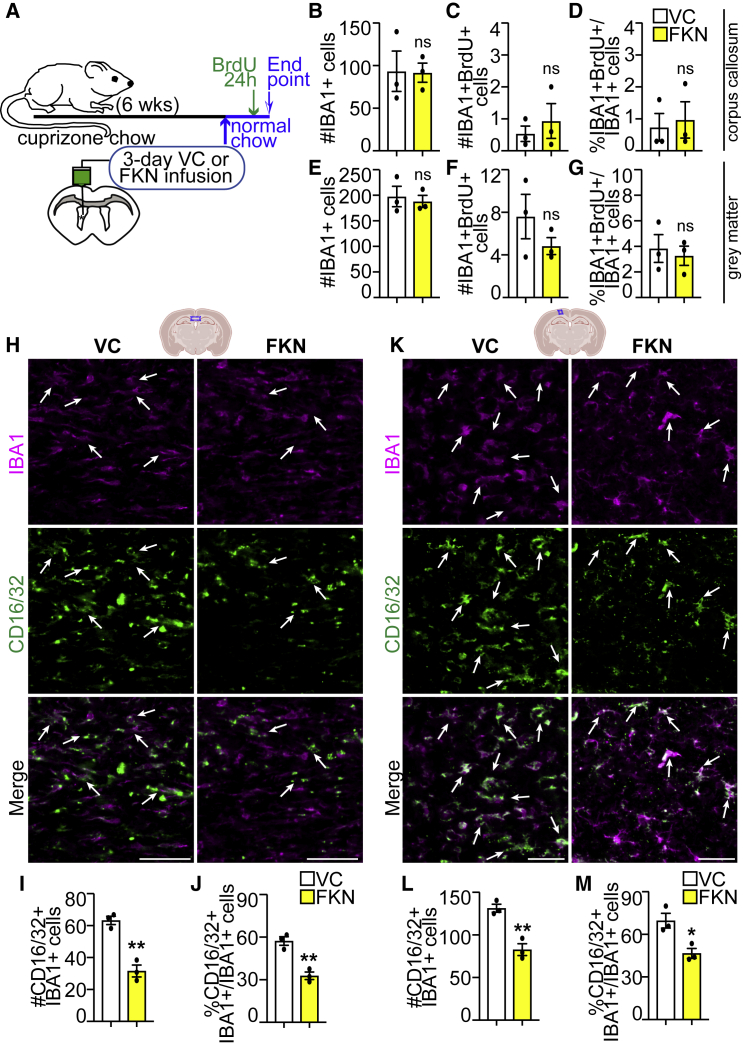
FKN attenuates microglia/macrophage activation in remyelinating brain (A) Ten-week-old C57BL/6J mice were fed cuprizone chow for 6 weeks. VC/FKN was infused into the lateral ventricle for 3 days after cuprizone cessation, and mice were returned to normal chow. BrdU was injected 24 h before euthanasia. (B–G) Analysis of midline CC (B–D) and cortical GM (E–G) for average number of total IBA1+ cells (B and E), IBA1+BrdU+ (C and F), and proliferative index of IBA1+ cells (%IBA1+BrdU+/IBA1+ cells [D and G]). (H and K) Representative images of midline CC (H) or cortical GM (K) immunostained for IBA1 (magenta) and CD16/32 (green) from VC- and FKN-infused mice. Arrows indicate IBA1+CD16/32+ microglia/macrophages. (I, J, L, and M) Quantification of (H and K). Scale bars, 50 μm. Error bars represent SEM. Data were analyzed using unpaired t test, ^∗∗^p < 0.01, ^∗^p < 0.05, ns = not significant. n = 3 mice per group. See also Figure S4.

Next, we tested whether exogenous FKN alters microglia biology *in vivo*. Cuprizone-treated WT mice were infused with VC/FKN for 3 days and 24 h before euthanasia, BrdU was injected to label proliferating cells (Figure 5A). Exogenous FKN did not alter the number of total IBA1+ cells, proliferating IBA1+BrdU+ cells, or the proliferative index of IBA1+ cells either in CC, or in cortical GM (Figures 5B–5G).

Previous reports demonstrated FKN infusion or FKN expression in CNS leads to changes in microglia/macrophage activation (Lyons et al., 2009; Pabon et al., 2011; Wang et al., 2021). Moreover, temporal regulation of microglia and macrophage activation is critical for efficient de- and remyelination (Lampron et al., 2015; Miron et al., 2013). In a cuprizone mouse model, the levels of iNOS, TNF-α, and TSPO, which are expressed in pro-inflammatory microglia/macrophages, increase by weeks 5–6 of cuprizone treatment, whereas the levels of CD206, CD163, and Arg1, which are expressed in alternate microglia/macrophages, do not significantly change during or after cuprizone treatment (Abdi et al., 2021; Klein et al., 2018). We therefore focused our analysis on CD16/32, which is expressed in pro-inflammatory microglia and macrophages (de Almeida et al., 2020; Miron et al., 2013).

At 3 days of FKN infusion, the proportion and number of CD16/32+IBA1+ microglia/macrophages in the CC and cortical GM were decreased (Figures 5H–5M). Notably, there was a small decrease in the number of CD16/32+IBA1+ microglia/macrophages in the cortical GM, but not CC at 7 days of FKN infusion (Figures S4A–S4E). Regarding astrocyte reactivity, there were no changes in the GFAP astrocyte signal intensity or GFAP+ cell number between VC- and FKN-infused brains (Figures S4F–S4N). Thus, FKN infusion leads to a reduction in pro-inflammatory microglia/macrophage, but not astrocyte, activation.

### Exogenous FKN enhances remyelination

**Figure 6:**
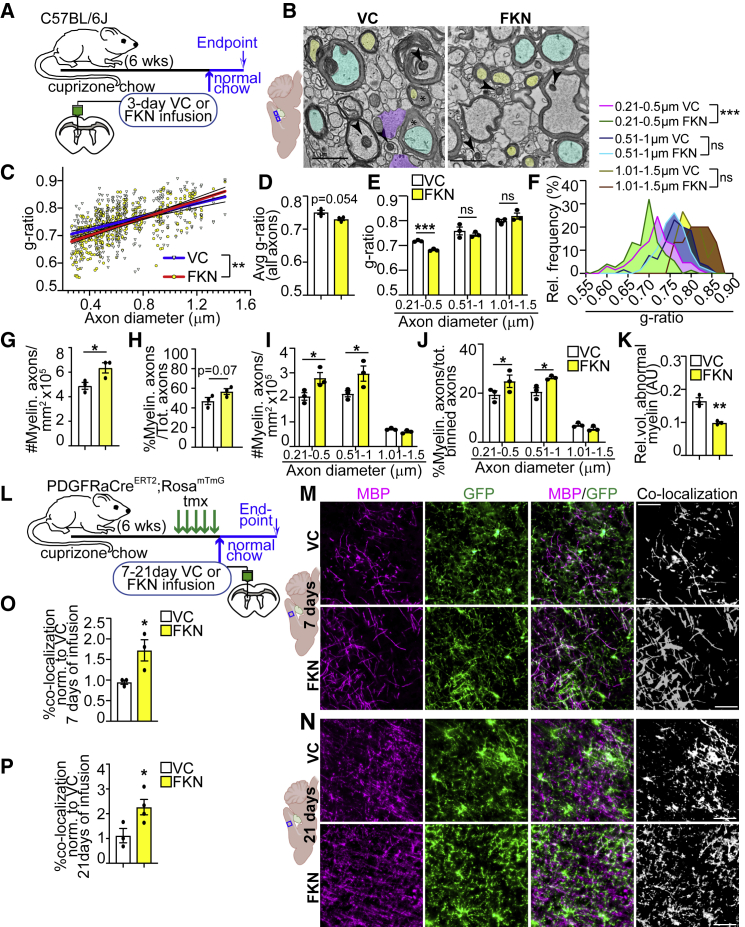
FKN enhances remyelination (A) Ten-week-old C57BL/6J mice were fed cuprizone chow for 6 weeks. VC/FKN was infused into the lateral ventricle for 3 days after cuprizone cessation, and mice were returned to normal chow. (B) Representative TEM images of CC sections from VC- and FKN-infused mice. Arrowheads designate well-preserved mitochondria, asterisks abnormal myelin, yellow and blue small and medium diameter axons, respectively, and purple glial fibrillar protein positive regions. (C–F) Analysis of (B) for g-ratio versus axon diameter correlation (C), average g-ratio for all axons (D), and average g-ratio (E) or g-ratio frequency distribution (F) for binned axons. (G–J) Analysis of (B) for total (G and H) or binned (I and J) myelinated axon density (G and I) or proportion (H and J). (K) Morphometric analysis of abnormal myelin. AU = arbitrary units. (L) Ten-week-old PDGFRαCre^ERT2^;Rosa^mT^^/^^mG^ were fed cuprizone chow for 6 weeks. Tamoxifen was injected in the last week of cuprizone treatment. VC/FKN was infused into the lateral ventricle after cuprizone cessation for 7–21 days and mice were returned to normal chow. (M and N) Representative images of VC- (top) and FKN- (bottom) infused cortical GM immunostained for MBP (magenta) and GFP (green) after 7 (M) and 21 (N) days of infusion. White represents MBP and GFP co-localization. (O and P) Quantification of (M), (N) normalized to VC after 7 (O) and 21 (P) days of infusion. Scale bars, 1 μm in (B), 50 μm in (M and N). Error bars represent SEM. Each data point in (C) represents an axon, and in (D–K) a mouse. Data were analyzed using unpaired t test, except data in (C and F) were analyzed with linear regression and data in (E, I, and J) with multiple t test. ^∗∗∗^p < 0.001, ^∗∗^p < 0.01, ^∗^p < 0.05, ns = not significant, n = 3–4 mice per group. See also Figure S5.

Since we observed an increase in oligodendrocyte densities and a reduction in microglia activation as early as 3 days of FKN infusion (Figures 2C and 5I), we performed transmission electron microscopy (TEM) on cuprizone-treated WT animals after 3 days of VC/FKN infusion to assess CC remyelination, similar to (Fernández-Castañeda et al., 2020) (Figures 6A and 6B). FKN infusion resulted in a slightly lower g-ratio (increased myelin thickness) for small-diameter axons with a reversed trend for large-diameter axons (Figure 6C). There was a non-significant trend toward lower average g-ratio for all diameter axons in the FKN-infused mice (Figure 6D). Frequency distribution of g-ratios, however, revealed that FKN caused a shift in g-ratio binning (Figure S5C). When g-ratios were binned into three axon diameter groups, there was a small FKN-mediated reduction in the g-ratio and g-ratio frequency for axons up to 0.5 μm diameter, and no significant changes for axons within 0.51–1.5 μm diameter (Figures 6E and 6F). Notably, there were no changes in axonal density or the relative abundance of axons within any of these bins (Figures S5D and S5E). Furthermore, FKN caused a significant increase in the density and a trending increase in the proportion of total myelinated axons (Figures 6G and 6H). When binned into three axon diameter groups, there was a significant increase in the density and proportion of both small (0.21–0.5 μm) and medium (0.51–1 μm) diameter axons with no changes for the large (1.01–1.5 μm) diameter axons (Figures 6I and 6J).

Next, we assessed differences in myelin or axonal health, critical parameters in myelin biogenesis, and neurodegeneration (Edgar et al., 2020; Peters, 2009). The relative volume of abnormal myelin, which included decompacted and redundant myelin, but not normal compact myelin, was decreased in FKN-infused animals (Figures 6K and S5F). The proportion of degenerating axons was not changed (Figure S5G).

Finally, we asked whether FKN increases cortical GM remyelination. We subjected 10-week-old PDGFRαCre^ERT2^;Rosa^mT/mG^ mice to a 6-week cuprizone chow, where tamoxifen was injected in the last week of cuprizone treatment. In these mice, all cells express TdTomato (mT). Upon tamoxifen injections, recombined cells express membranous GFP (mG) (Muzumdar et al., 2007). Since myelin is enriched in the oligodendrocyte cell membranes, a co-localization analysis between MBP, which is highly enriched in compact myelin (Snaidero et al., 2017), and mG is indicative of the formation of new myelin arising from differentiating PDGFRα+ OPCs. VC or FKN were infused for 7 or 21 days, during which time mice were returned to normal chow (Figure 6L). FKN increased new myelin formation by ∼1.8- to 2-fold when compared with VC after 7–21 days of infusion (Figures 6M−6P). Notably, these results are comparable to clemastine, the only remyelinating agent currently in clinical trials (Li et al., 2015).

Taken together, these results show FKN increases remyelination in both CC and cortical GM.

### FKN modulates proliferation and differentiation of OPCs cultured with and without microglia

**Figure 7:**
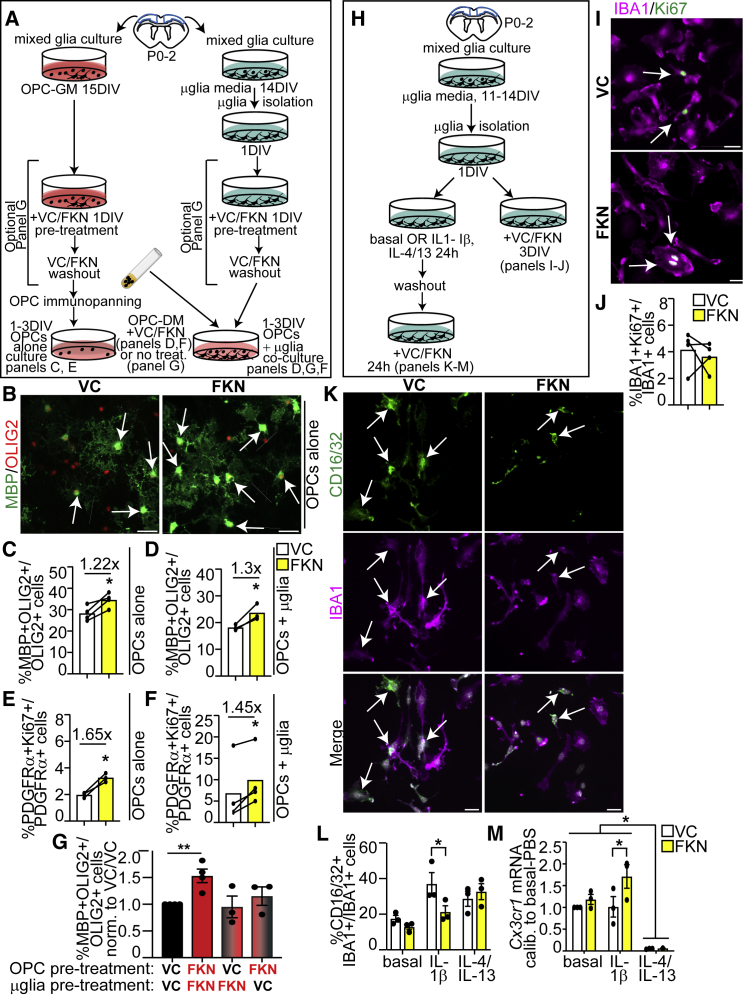
FKN regulates OPC and microglia cell fates *in vitro* (A) OPCs were cultured with or without microglia in OPC DM and VC/FKN for 1–3 days *in vitro* (DIV). (B) Representative images of OPCs cultured in VC or FKN for 3DIV and immunostained for MBP (green) and OLIG2 (red). Arrows designate MBP+OLIG2+ oligodendrocytes. (C–F) Quantification of % MBP+Olig2+ cells at 3DIV (C and D) and OPC proliferative index at 1DIV (E and F) in OPC cultures (C, E) or OPC-microglia co-cultures (D and F). (G) Quantification of % MBP+OLIG2+ oligodendrocytes in co-cultures with VC- and/or FKN-pre-treated OPCs and microglia. (H) Microglia were incubated with VC/FKN with or without pre-stimulation with IL-1β and IL-4/IL-13 cytokines or no cytokines (basal conditions). (I) Representative images of microglia treated with VC/FKN for 3DIV and immunostained with IBA1 (purple) and Ki67 (green). (J) Quantification of (I). (K) Representative images of IL-1β-pre-stimulated microglia treated with VC/FKN and immunostained with CD16/32 (green), IBA1 (purple) and counterstained with Hoechst (white). (L) Quantification of (K). (M) qPCR analysis of *Cx3cr1* mRNA expression normalized to *Gapdh* and *Hnrnpab* housekeeping genes and calibrated against basal VC-treated microglia. Scale bars, 20 μm. Error bars represent SEM. Each connected line corresponds to one biological replicate. Data were analyzed with Student’s paired t test, except graph in (G) was analyzed with one-way ANOVA followed by Dunnett’s post hoc test, graph in (L) was with multiple t test and graph in (M) with two-way ANOVA followed by Tukey post hoc test. ^∗∗^p < 0.01, ^∗^p < 0.05, n = 3–4 independent experiments. See also Figure S6.

To ask whether FKN modulates OPC biology directly or indirectly via actions on microglia, we cultured isolated primary cortical OPCs and microglia (Figures 7A, 7H, S6A–S6D, S6E–S6G), alone or together.

Exogenous FKN increased the proportion of MBP+OLIG2+ oligodendrocytes from OPCs cultured in the absence or presence of microglia (Figures 7B–7D) in OPC differentiation media containing T3 (triiodothyronine), a potent inducer of oligodendrocyte differentiation (herein referred to as OPC-DM) (Watson et al., 2021). It is known that PDGF-AA is highly expressed in CC but not cortical GM, and that OPCs in the white matter, but not GM, proliferate in response to PDGF-AA (Hill et al., 2013; Krupinski et al., 1997). We thus asked whether FKN modulates OPC proliferation in OPC-DM, which is devoid of growth factors. Figures S6D, 7E, and 7F show FKN increased OPC proliferative index in the presence and absence of microglia. Therefore, FKN enhances OPC proliferation and differentiation at least in part by directly affecting OPCs.

Next, we investigated changes in the interplay between OPCs and microglia after exposure to FKN. We pre-treated OPCs and/or microglia with VC or FKN, followed by VC/FKN washout and subsequent co-cultures of pre-treated cells in OPC-DM (without additional FKN) (Figure 7A). Figure 7G shows that the proportion of MBP+OLIG2+ oligodendrocytes was only increased in co-cultures, in which both OPCs and microglia were pre-treated with FKN. Pre-treating only OPCs or microglia with FKN did not alter the proportion of oligodendrocytes (Figure 7G).

These results provide a novel mechanistic insight into FKN-CX3CR1 signaling and demonstrate that FKN acts on both OPCs and microglia to increase oligodendrocyte formation.

### FKN modulates microglia biology *in vitro*

Next, we assessed *in vitro* microglia response to FKN. Exogenous FKN did not alter the proportion or proliferative index of IBA1+ microglia (Figures S6F, 7I, and 7J). We then incubated microglia with or without polarizing cytokines IL-1β or IL-4/IL-13 for 1DIV (Figure 7H). We confirmed TNF-α, a pro-inflammatory cytokine, was upregulated specifically in media conditioned by IL-1β pre-treated microglia (Figure S6H). Incubation of basal or pre-stimulated microglia with FKN did not affect the proportion of total IBA1+ microglia in basal, interleukin (IL)-1β, or IL-4/IL-13 conditions (Figures 7H and S6G). However, FKN reduced the proportion of CD16/32+IBA1+ IL-1β pre-stimulated microglia by ∼1.43-fold (Figures 7K and 7L). Thus, in agreement with *in vivo* results, FKN reduces CD16/32+ microglia *in vitro*.

It was recently shown that Cx3cr1 overexpression reduces pro-inflammatory microglia/macrophages or mediators (Inoue et al., 2021; Zhang et al., 2019). We thus hypothesized that FKN could be attenuating microglia activation by modulating Cx3cr1 expression. qRT-PCR revealed *Cx3cr1* mRNA was expressed in basal and IL-1β pre-stimulated microglia, but its expression was negligible in IL-4/IL-13 pre-stimulated microglia (Figure 7M). Notably, *Cx3cr1* mRNA was ∼1.7-fold upregulated by FKN treatment specifically in the IL-1β pre-stimulated microglia (Figure 7M). Therefore, FKN reduces pro-inflammatory microglia activation and upregulates *Cx3cr1* receptor expression.

Finally, we determined the phagocytic ability of microglia in the presence of FKN. Here, we treated isolated primary murine microglia with VC/FKN for 3 h in the presence of myelin debris conjugated to pHRodo, which fluoresces in the acidic lysosomal environment (pH ∼5.5) (Bhattacherjee et al., 2019) (Figure S6I). Figures S6J and S6K shows FKN-treated microglia had an ∼1.2-fold increase in pHRodo mean fluorescence intensity when compared with VC. Thus, FKN increases myelin debris phagocytosis by microglia *in vitro*.

## Discussion

Our data demonstrate exogenous FKN engages parenchymal OPCs, which express *Cx3cr1*, for oligodendrocyte regeneration and remyelination in a cuprizone demyelination mouse model.

Prior reports demonstrate administration of exogenous soluble FKN before induction of stroke or Parkinson-like lesions leads to neuroprotection (Cipriani et al., 2011; Morganti et al., 2012). However, the ability of FKN to promote regeneration after a demyelinating injury has not been tested. We show that ICV administration of soluble FKN after cuprizone-induced demyelination leads to increased production of *de novo* oligodendrocytes and remyelination *in vivo* from activated parenchymal OPCs.

Our results also show exogenous FKN increases small-diameter axon myelination. Intriguingly, patients with MS show specific neurodegeneration of small caliber axons (Evangelou et al., 2001; Lassmann, 2003). Our work highlights the potential neuroprotective role of FKN for these small caliber axons and raises an intriguing question about oligodendrocyte-axon interaction. Pío del Río-Hortega described four types of oligodendrocytes, from which type I and II oligodendrocytes myelinate small-diameter axons (reviewed in Simons and Nave [2015]). While we have previously shown FKN increases oligodendrocyte-axon interactions (Watson et al., 2021), we do not currently understand whether FKN stimulates the generation of specific oligodendrocyte subtype(s) that may have a preference for small-diameter axon myelination, and the biological significance of this phenomenon.

Notably, it was previously demonstrated that a CX3CR1 allosteric modulator AZD8797, which is proposed to act as an antagonist and which does not cross the blood brain barrier, protects from autoimmune demyelination by blocking peripheral leukocyte infiltration (Ridderstad Wollberg et al., 2014). On the other hand, FKN infusion or overexpression specifically in the CNS has been shown to have beneficial effects in normal healthy mice as well as rodent models of stress, stroke, schizophrenia, synucleinopathy, as well as Parkinson and Alzheimer diseases (Watson et al., 2021, and reviewed in Watson et al., 2020). Our results extend these reports and demonstrate that infusion of FKN into demyelinated CNS increases oligodendrocyte and myelin regeneration.

Our experimental design does not allow distinguishing direct *in vivo* effects of FKN on OPCs versus microglia. Our *in vitro* experiments, however, show FKN directly modulates OPC fates when cultured without microglia. Surprisingly, our co-culture results demonstrate that both OPCs and microglia need to be pre-treated with FKN to elicit an increase in oligodendrocyte formation. These results suggest that FKN acts on both OPCs and microglia to increase oligodendrocyte formation.

We also show CC and cortical GM OPCs display a differential proliferation response to FKN infusion, which was not associated with a differential response in microglia or astrocyte activation. It was previously shown that white matter and GM OPCs are transcriptionally homogeneous yet respond differently to IFNγ (Sherafat et al., 2021a). In the future, it will be interesting to determine whether FKN elicits different signaling cascades in white matter versus GM OPCs, and/or whether this is mediated by other cell types.

In summary, we demonstrate that infusion of FKN into the brain after demyelinating injury increases oligodendrocyte and myelin regeneration from parenchymal OPCs. Therefore, FKN represents a novel candidate for remyelination strategies.

## Experimental procedures

### Resource availability

#### Corresponding author

Further information and requests for resources and reagents should be directed to and fulfilled by the lead contact, Anastassia Voronova (voronova@ualberta.ca).

#### Materials availability

This study did not generate new unique reagents.

### Growth factors and cytokines

For cell cultures, murine soluble FKN (R&D Systems) was reconstituted in 1X PBS and used at 250 ng/mL. For ICV infusions, FKN was reconstituted in 0.2% BSA in 1X PBS and infused at 200 ng/day rate using Alzet osmotic mini-pumps as per Watson et al. (2021). Please see supplemental materials and methods for other details.

### Experimental model and subject details

#### Mice

Animal use protocols were approved by the Research Ethics Office at the Universities of Alberta and Ottawa in accordance with the Canadian Council of Animal Care Policies. Mice from both sexes were used for all experiments. PDGFRαCre^ERT2^ (*B6N.Cg-Tg(Pdgfra-cre/ERT)467Dbe/J*; RRID:IMSR_JAX:018280), RosaYFP^STOP^ (*B6.129X1-Gt(ROSA)26Sortm1(EYFP)Cos/J*; RRID:IMSR_JAX:006148), Rosa^mT/mG^ (*B6.129(Cg)-Gt(ROSA)26Sortm4(ACTB-tdTomato,-EGFP)Luo/J*; RRID:IMSR_JAX:007676) and WT C57BL/6J mice were obtained from Jackson Laboratories (Kang et al., 2010; Muzumdar et al., 2007; Srinivas et al., 2001). CD1 mice were purchased from Charles River Laboratory.

#### Cuprizone-induced demyelination experiments

Ten-week-old mice were subjected to nutragel (Bio-Serv) *ad libitum* containing 0.2% cuprizone (Sigma) for 6 weeks.

#### Tamoxifen injections

PDGFRαCre^ERT2^;RosaYFP^STOP^ or PDGFRαCre^ERT2^;Rosa^mT/mG^ animals were injected with 3 mg tamoxifen for 5 days as described in Li et al. (2022) and Watson et al. (2021). Seventy-two hours after last tamoxifen injection, ICV surgery was performed as per below.

#### ICV infusions

Infusions are described in Li et al. (2022) and Watson et al. (2021). Briefly, infusion was performed into the right ventricle using the following coordinates relative to bregma: −1.000 ML, −0.300 AP, −2.500 DV. For one-time injection, 0.5–1 μL of FKN-647 or matched volume and equimolar amount of BSA-647 were injected once into 2- to 3-month-old WT C57BL/6J mice as described in Watson et al. (2021). For multi-day infusion, 7-day or 28-day osmotic mini-pumps (Alzet, 1007D or 1004) were connected containing VC (vehicle-control) or FKN in VC. After surgery, mice were returned to normal chow. BrdU (Sigma) was injected at 100 mg/kg dose 24 h before perfusions.

#### Lysolecithin injections

Lysolecithin (LPC)-mediated demyelination was performed as described in Kosaraju et al. (2020). Briefly, 1-month-old WT mice were injected with LPC (Sigma, L1381) (1 μL of 1% solution in 1x PBS) using a stereotactic apparatus at two sites: +1.0 mm AP, +1.0 mm ML, −2.2 mm DV and +0.7 mm AP, +1.0ML, −2.2 DV. Animals were allowed to recover for 14 days.

#### Myelin isolation

Myelin was isolated as described in Bhattacherjee et al. (2019). Briefly, brains from 5-month-old Sprague-Dawley rats were homogenized, layered over sucrose, ultracentrifuged, and purified via density gradients. Purified myelin was labeled with pHRodo (Invitrogen) and stored at −80°C.

#### Tissue dissection and collection

Mice under 21 days of age were euthanized with CO_2_, and over 21 days with 102 mg/kg of body weight Euthansol (WDDC), followed by transcardiac perfusion with HBSS and 4% paraformaldehyde (PFA, Acros Organics). For TEM, animals were perfused with HBSS followed by 4% PFA in 0.1M Sodium Cacodylate buffer with 2 mM CaCl_2_. Dissected regions of interest were incubated with 2% PFA, 2.5% Glutaraldehyde, 0.1M Sodium Cacodylate buffer, and 2 mM CaCl_2_, infiltrated with Spurr’s resin and polymerized. Sections were cut from two blocks per sample using an ultramicrotome (Leica, EM UC6).

#### Primary cell cultures

Complete methods are in the supplemental information.

##### Microglia cultures

Microglia were cultured from P0-P2 CD1 cortices in DMEM/F12 (Gibco) with 10% FBS (Sigma), 1% Pen/Strep, 1% sodium pyruvate, and 50 μM β-mercaptoethanol (herein referred to as **microglia media**) as per Galleguillos et al. (2022) with slight modifications. Namely, 10 ng/mL GM-CSF was added on days 8–11. Microglia were isolated on day 14 using lidocaine (Sigma) and shaking. For proliferation analysis, microglia were cultured with VC/FKN and without β-mercaptoethanol for 3 days. For co-cultures, isolated microglia were optionally pre-treated with VC/FKN for 24 h. Please see supplemental materials and methods for more details. VC/FKN was washed off, and isolated OPCs (with or without VC/FKN pre-treatment) were plated on top of microglia as described below.

##### Cortical OPC cultures

OPCs were cultured from P0-P2 CD1 cortices in Serum-Free Media (SFM; please see supplemental materials and methods for details) supplemented with 2% B27 supplement (Life Technologies), 10 ng/mL FGF (Peprotech), and 10 ng/mL PDGF-AA (R&D) (herein referred to **OPC growth media [OPC-GM]**). On day 15, cultures were optionally pre-treated with VC or FKN for 24 h. On day 16, VC/FKN was washed off. OPCs were immunopanned on day 15 (without pre-treatment) or day 16 (when cells were pre-treated) with PDGFRα-specific magnetic beads (Miltenyi). Recovered cells were cultured on top of microglia or alone in SFM with 2% B27 and 40 ng/mL 3,3′,5-Triiodo-L-thyronine (T3, Sigma) and VC/FKN (herein referred to **OPC differentiation media [OPC-DM]**) for 1–3 days.

##### Microglia polarization

Isolated microglia were treated with no cytokines (basal conditions), 10 ng/mL IL-1β (Cedarlane), or 20 ng/mL IL-4 (Miltenyi) and IL-13 (R&D Systems) for 24 h. Media was changed and VC/FKN was added for additional 24 h.

##### Myelin phagocytosis assays

pHRodo-Myelin and VC/FKN was added to microglia cultured in basal conditions for 3 h and imaged as described below.

##### Conditioned medium preparation/ELISA

Conditioned medium was collected from pre-stimulated microglia, centrifuged at 2,000 × *g* for 7 min to remove dead cells, and subjected to TNF-α ELISA kit (Invitrogen).

### Reagents and immunostaining

#### Immunocytochemistry

Cell cultures were fixed and stained as described in Watson et al. (2021) with antibodies listed in the supplemental materials and methods. Nuclei were counterstained with Hoechst 33,258 (Riodel-De Haen Ag).

#### Immunohistochemistry

Cryopreserved brains were sectioned at 18 μm. For FKN-647 diffusion assay, sections were rehydrated in 1X PBS, immediately counterstained with Hoechst 33258 to visualize nuclei. Staining with FluoroMyelin was performed as per manufacturer’s instructions (Invitrogen). For all other staining, sections were stained as described in Li et al. (2022) and Watson et al. (2021). Antibodies are listed in the supplemental materials and methods. Nuclei were counterstained with Hoechst 33258.

#### RNA scope

Brain cryosections from WT demyelinated mice were subjected to RNA scope as described in Voronova et al. (2017) and Watson et al. (2021) with probes targeting murine *Cx3cr1* and/or *Pdgfrα* mRNA or negative control probe according to the manufacturer’s instructions (ACD). Some sections were counterstained with anti-IBA1 antibodies as in Voronova et al. (2017).

#### Microscopy

Brain sections were imaged with (1) Zeiss LSM700 confocal microscope with photomultiplier tube (PMT) with ×40 objective, where high-magnification digital image acquisition was performed with 2–5x digital Zoom using Zen (Zeiss), (2) Olympus IX81 fluorescence microscope equipped with Okogawa CSU X1 spinning disk confocal scan head, ×20 objective and a Hamamatsu camera (Hamamatsu), where digital image acquisition was performed with Volocity (Perkin Elmer) software, or (3) with Zeiss Axio Imager M2 fluorescence microscope, ORCA-Flash LT sCMOS Camera and the Zen software (Zeiss). Z-stacks spanning 6–10 μm were taken with optical slice thickness 0.2–0.5 μm and stacked images or orthogonal sections through 3D projections are shown.

Images from all fixed primary cell culture experiments were captured with ×20 objective using Zeiss Axio Imager M2 fluorescence microscope, ORCA-Flash LT sCMOS Camera, and the Zen software (Zeiss). Images were captured in a single plane.

Live microglia cultures were imaged using an inverted Zeiss Axio Observer Z1 microscope equipped with Axiocam 503 Mono camera and ×20 objective.

For TEM, images were acquired using JEOL JEM-2100, Gatan Orius camera with digital micrograph at 200 kV acceleration voltage.

#### RNA isolation and qRT-PCR

Total RNA was purified using Omega Biotek E.Z.N.A. microelute kit, and reverse transcribed to generate cDNA using QuantiTect Reverse Transcription Kit (Qiagen) as described in Voronova et al. (2017). qPCR was performed using the Ssoadvanced SYBR Green kit (BioRad), primers listed in the supplemental materials and methods, and using Eppendorf Realplex2 (Eppendorf) instrument. Data were normalized to *Gapdh* and *Hnrnpab* and analyzed using 2^−ΔΔCt^ method.

### Quantification, co-localization and statistical analysis

*In vitro* data are presented from at least three independent biological experiments. Five to 10 fields of view and at least 250 cells from each treatment and biological experiment were counted.

*In vivo* analysis was performed in a blind fashion by two independent observers. Five matched sections per brain were analyzed from six mice across three independent litters. At least 300 cells per CC and 800 cells per cortical column in every sample were counted.

For co-localization analysis, cortical GM was imaged using ×20 objective and captured using Z-stacks with an optical thickness of 0.5 μm spanning 5–8 μm. Z-stacks were subjected to Imaris and voxel-based MBP/mGFP co-localization analysis was performed.

TEM images were analyzed as described in Bando et al. (2015) and Edgar et al. (2020). In brief, the g-ratio was measured for myelinated axons, where axon and compact myelin morphology were normal. At least eight images and 100 axons per sample were analyzed. The number of intersections of normal and abnormal myelin with 64 equidistant grids overlaid in Fiji software was counted. Axonal density was analyzed using axons that fit within the ROI. At least 30 images per sample were analyzed.

All data were subjected to normality tests with D’Agostino & Pearson, Shapiro-Wilk, and Kolmogorov-Smirnov tests and were considered normal. For two group comparisons, two-tailed paired Student’s t tests (*in vitro* datasets) or two-tailed unpaired Student’s t tests (for *in vivo* datasets) or multiple t tests were used to assess statistical significance between means, where a p value <0.05 was considered significant. For three or more group comparisons, one-way or two-way ANOVA followed by Dunnett’s or Tukey multiple comparisons tests were used. For comparing slopes of g-ratios with axon diameters, simple linear regression was applied where 95% CI of the best-fit line was used.

## Data Availability

This study did not generate new unique datasets.

## Supplemental information

Document S1. Figures S1–S6 and Supplemental experimental procedures

Document S2. Article plus supplemental information
