# Supplementary material for: Fractalkine enhances oligodendrocyte regeneration and remyelination in a demyelination mouse model
Source: Stem Cell Reports. 2023 Jan 5;18(2):519–33. doi: 10.1016/j.stemcr.2022.12.001 (PMC9968989; doi:10.1016/j.stemcr.2022.12.001)
Supplement: Document S1. Figures S1–S6 and Supplemental experimental procedures [file mmc1.pdf]

Stem Cell Reports, Volume *18*

Supplemental Information

Fractalkine enhances oligodendrocyte regeneration and remyelination in a demyelination mouse model

Monique M.A. de Almeida, Adrianne E.S. Watson, Sana Bibi, Nicole L. Dittmann, Kara Goodkey, Pedram Sharafodinzadeh, Danny Galleguillos, Maryam Nakhaei-Nejad, Jayasankar Kosaraju, Noam Steinberg, Beatrix S. Wang, Tim Footz, Fabrizio Giuliani, Jing Wang, Simonetta Sipione, Julia M. Edgar, and Anastassia Voronova

Figure S1

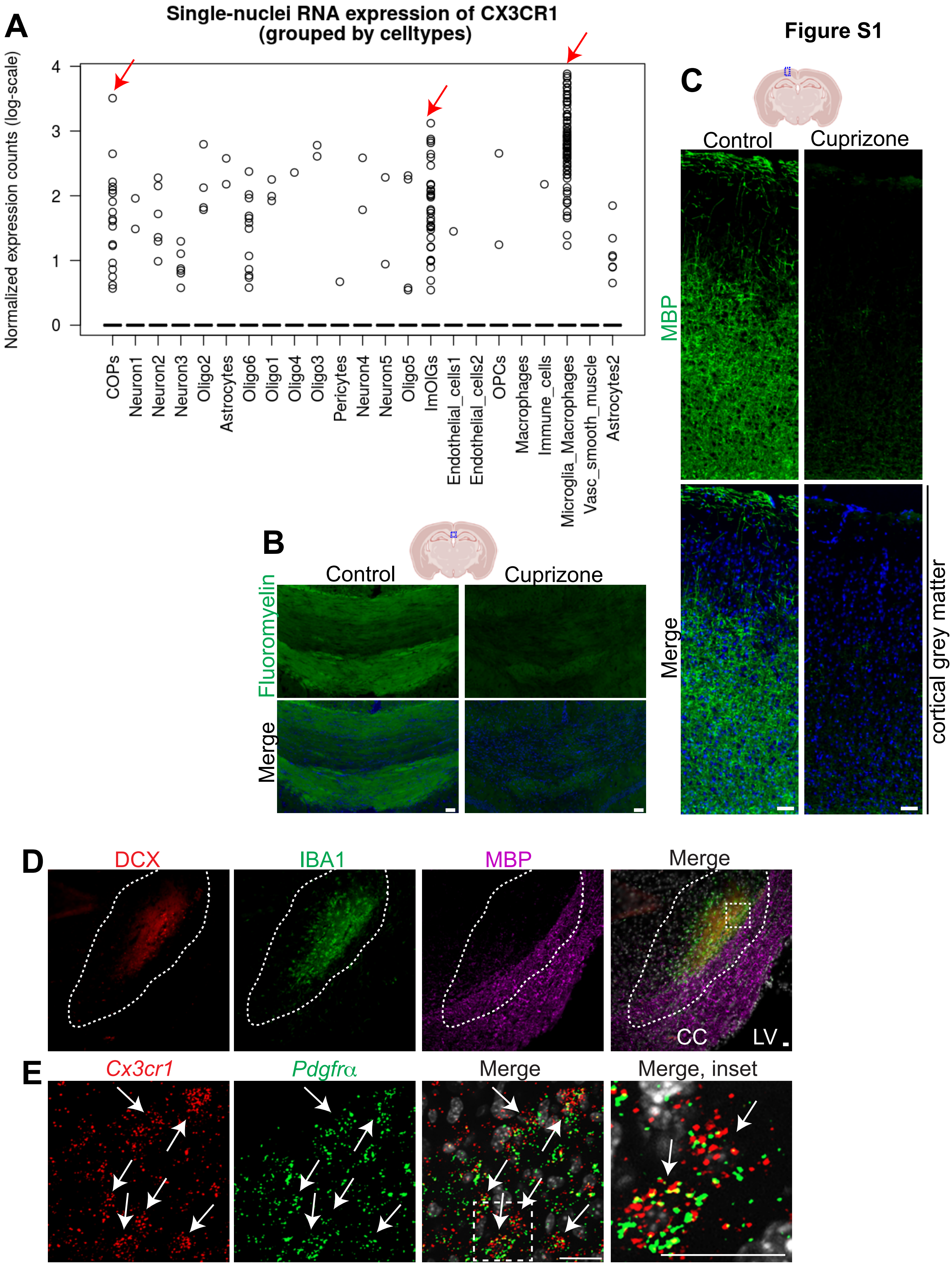

A
Single-nuclei RNA expression of CX3CR1
(grouped by celltypes)
Normalized expression counts (log-scale)
0
1
2
3
4
COPs
Neuron1
Neuron2
Neuron3
Oligo2
Astrocytes
Oligo6
Oligo1
Oligo4
Oligo3
Pericytes
Neuron4
Neuron5
Oligo5
ImOlGs
Endothelial_cells1
Endothelial_cells2
OPCs
Macrophages
Immune_cells
Microglia_Macrophages
Vasc_smooth_muscle
Astrocytes2
B
Control
Cuprizone
Fluoromyelin
Merge
C
Control
Cuprizone
MBP
Merge
cortical grey matter
D
DCX
IBA1
MBP
Merge
CC
LV
E
Cx3cr1
Pdgfrα
Merge
Merge, inset

Figure S1. Related to Figure 1. ***Cx3cr1 mRNA is expressed in OPCs and microglia/macrophages.*** **A.** *CX3CR1* mRNA expression extracted from single nuclei RNA sequencing from brain white matter of individuals with progressive MS (Jäkel et al., 2019), https://castelobranco.shinyapps.io/MSCtrl_CCA_18/). ImOLGs=Immune oligodendroglia; Oligo = Oligodendrocyte cluster; cOPC=committed OPC. Red arrows designate cell clusters with enriched *CX3CR1* mRNA expression. **B-C**. Representative images of mice fed normal chow (control, left) and demyelinated mice subjected to cuprizone chow for 6 weeks (cuprizone, right). Myelin is detected with Fluoromyelin stain in corpus callosum (green, **B**) and MBP in cortical grey matter (green, **C**). Images counter-stained with Hoechst 33258 (blue) and are representative of areas analyzed throughout the paper when referring to midline corpus callosum and cortical grey matter. **D.** Representative image of 1-month old mouse injected with lysolethicin into corpus callosum and immunostained for DCX (red), IBA1 (green) and MBP (purple). Dashed lines indicate demyelination and DCX/IBA1 cell infiltration boundary. **E**. Double-label RNA scope analysis of lysolethicin-demyelinated corpus callosum for *Cx3cr1* (red) and *Pdgfr*α (green) mRNAs. Approximate location of image is shown in hatched box in C in Merge panel. Arrows indicate *Cxc3r1*-postive, *Pdgfr*α-positive cells. Inset in hatched box is shown on the right side. Scale bars are 50 µm in **B-C** and 20 µm in **D-E**. LV = lateral ventricle.

Figure S2

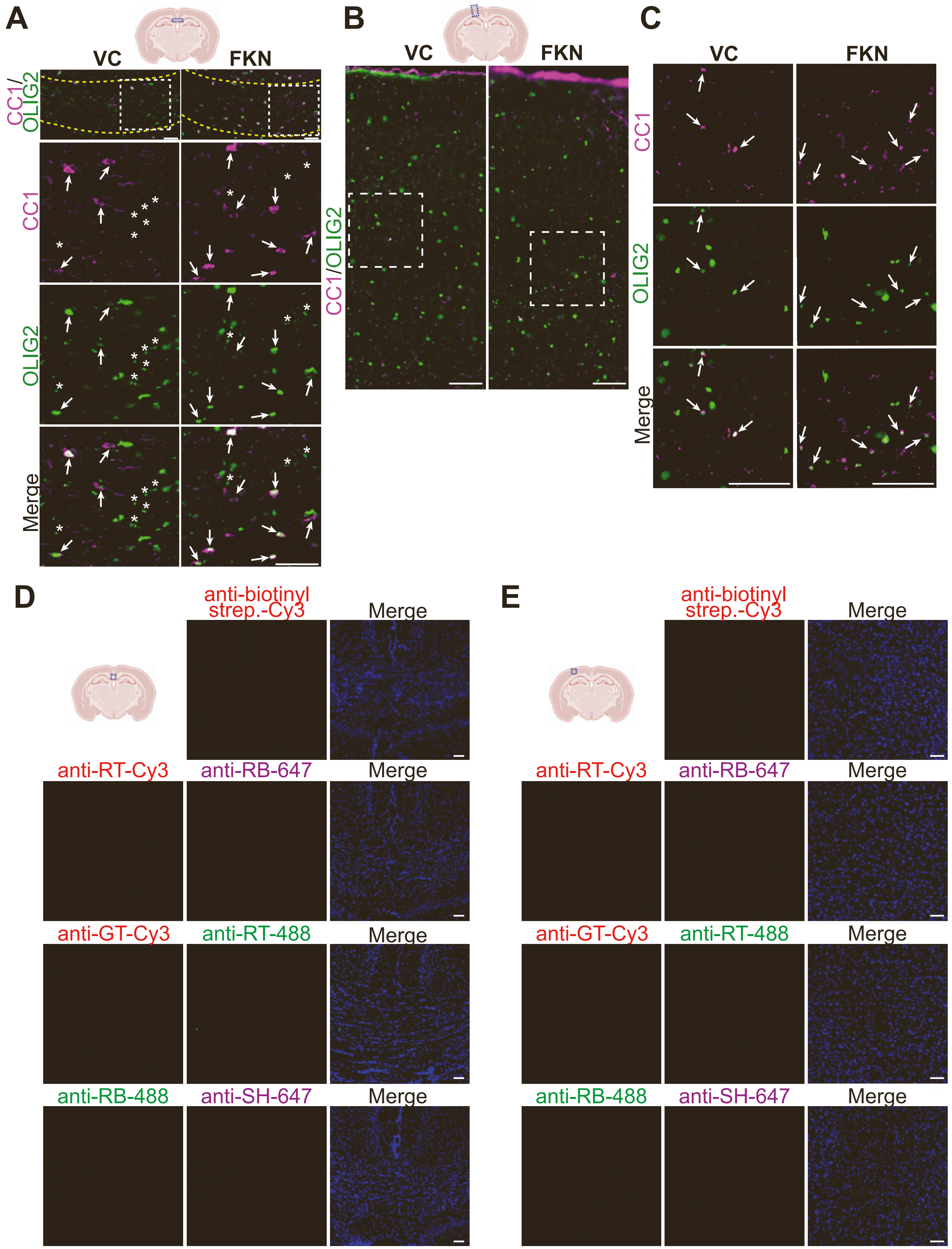
A
VC
FKN
CC1/OLIG2
CC1
OLIG2
Merge
B
VC
FKN
CC1/OLIG2
C
VC
FKN
CC1
OLIG2
Merge
D
anti-biotinyl strep.-Cy3
Merge
anti-RT-Cy3
anti-RB-647
Merge
anti-GT-Cy3
anti-RT-488
Merge
anti-RB-488
anti-SH-647
Merge
E
anti-biotinyl strep.-Cy3
Merge
anti-RT-Cy3
anti-RB-647
Merge
anti-GT-Cy3
anti-RT-488
Merge
anti-RB-488
anti-SH-647
Merge

Figure S2. Related to Figure 2. ***FKN infusion increases oligodendrocyte density after demyelination.*** **A**. Top: representative images of midline corpus callosum immunostained for CC1 (magenta) and OLIG2 (green) from demyelinated VC- and FKN-infused mice (please see Fig. 2A for experimental design). Approximate location is indicated by blue rectangle in the brain schematic. Dashed squares from top are shown at higher magnification in the bottom panels. Arrows indicate double-positive CC1+OLIG2+ oligodendrocytes, and asterisks OLIG2+ punctate staining (please see Fig. 2H for more details). Marker+ cells were quantified in Fig. 2C-D in the area between yellow hatched lines. Scale bar: 50 µm. **B-C.** Representative images of cortical column (**B**) immunostained for CC1 (magenta) and OLIG2 (green) from VC- and FKN-infused mice. Approximate location is indicated by blue rectangle in the brain schematic. Dashed squares from **B** are shown at higher magnification in **C**. Arrows indicate double-positive CC1+OLIG2+ oligodendrocytes. Scale bar: 200 µm. **D-E.** Secondary antibody controls in medial midline corpus callosum (**D**) and cortical grey matter (**E**). Brain sections from mice treated with cuprizone chow for 6 weeks were returned for 3 (**D**) and 7 (**E**) days to normal chow. Resulting brain sections were processed and stained as described as in the "Immunohistochemistry" section in Materials and Methods with the exclusion of primary antibodies. Sections were counter-stained with Hoechst 33258 (blue; "Merge" panel). Approximate location is indicated by blue rectangle in the brain schematics. GT = goat, RB = rabbit, RT = rat, SH = sheep, Strep = streptavidin. Scale bars are 50 µm.

Figure S3

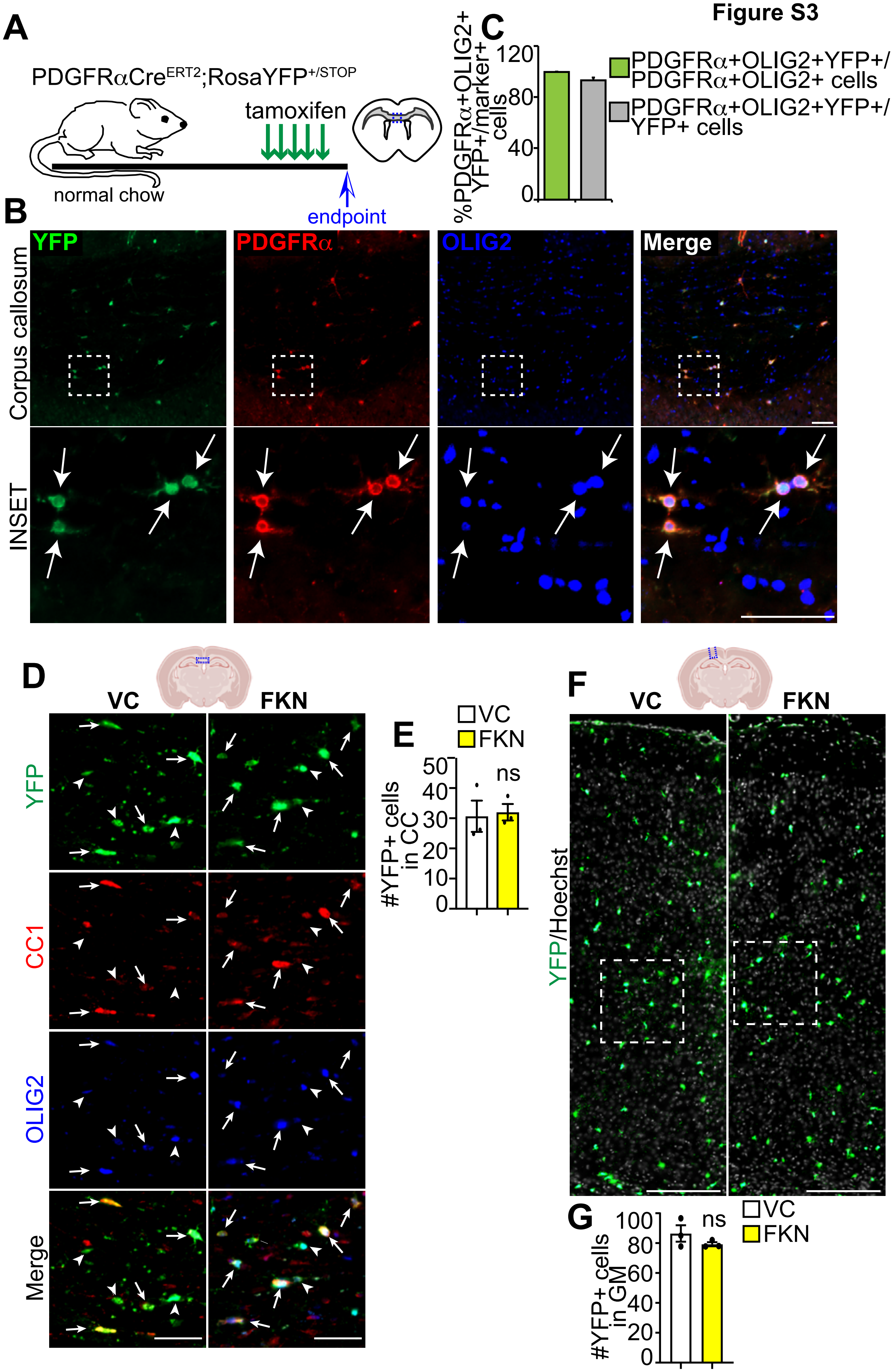
A
PDGFRαCre^{ERT2};RosaYFP$^{+/STOP}$
tamoxifen
normal chow
endpoint
C
%PDGFRα+OLIG2+ YFP+/marker+ cells
120
80
40
0
PDGFRα+OLIG2+YFP+/ PDGFRα+OLIG2+ cells
PDGFRα+OLIG2+YFP+/ YFP+ cells
B
Corpus callosum
INSET
YFP
PDGFRα
OLIG2
Merge
D
VC
FKN
YFP
CC1
OLIG2
Merge
E
VC
FKN
#YFP+ cells in CC
50
40
30
20
10
0
ns
F
VC
FKN
YFP/Hoechst
G
#YFP+ cells in GM
100
80
60
40
20
0
ns
VC
FKN

Figure S3. Related to Figure 3. ***FKN infusion increases de novo oligodendrocyte formation after demyelination*** **A**. Schematic illustration of **B-C**. 10 week-old PDGFRαCre^{ERT2};RosaYFP$^{STOP/+}$ were injected with tamoxifen once daily for 5 days. 72 hours later, mice were perfused. **B.** Representative images of midline corpus callosum immunostained for YFP (green), PDGFRα (red) and OLIG2 (blue). Approximate location is indicated by blue rectangle in the brain schematic. Arrows indicate triple-positive YFP+PDGFRα+OLIG2+ recombined OPCs. Scale bar: 50 µm. **C.** Quantification of **B** for % PDGFRα+OLIG2+YFP+/PDGFRα+OLIG2+ cells (green bar) and % PDGFRα+OLIG2+YFP+/YFP+ cells (grey bar). n=3 mice, error bars represent SEM. **D.** Representative images of midline corpus callosum immunostained for YFP (green), CC1 (red) and OLIG2 (blue) from remyelinating VC- and FKN-infused PDGFRαCre^{ERT2};RosaYFP$^{STOP/+}$ mice (please see Fig. 3C for experimental design). Approximate location is indicated by blue rectangle in the brain schematic. Arrows indicate triple-positive YFP+CC1+OLIG2+ newborn oligodendrocytes. Arrowheads indicate YFP+CC1-OLIG2+ cells. Scale bar: 50 µm. **E.** Quantification of Fig S3D for average number of YFP+ cells per section in the medial midline corpus callosum. * $p<0.05$. n=3 mice per group from at least 2 independent litters **F.** Representative images of cortical grey matter column immunostained for YFP (green) and counterstained with Hoechst 33258 (white) from remyelinating VC- and FKN-infused PDGFRαCre^{ERT2};RosaYFP$^{STOP/+}$ mice (please see Fig. 3C for experimental design). Approximate location is indicated by a blue rectangle in the brain schematic. Dashed squares are shown at higher magnification in Fig. 3K. **G.** Quantification of **F** for average number of YFP+ cells per section. n=3 mice per group from at least 2 independent litters. 3-5 anatomically matched sections per brain were analyzed with at least 300 cells counted per brain. For all graphs error bars represent SEM. Data was analyzed using unpaired t-test.

Figure S4

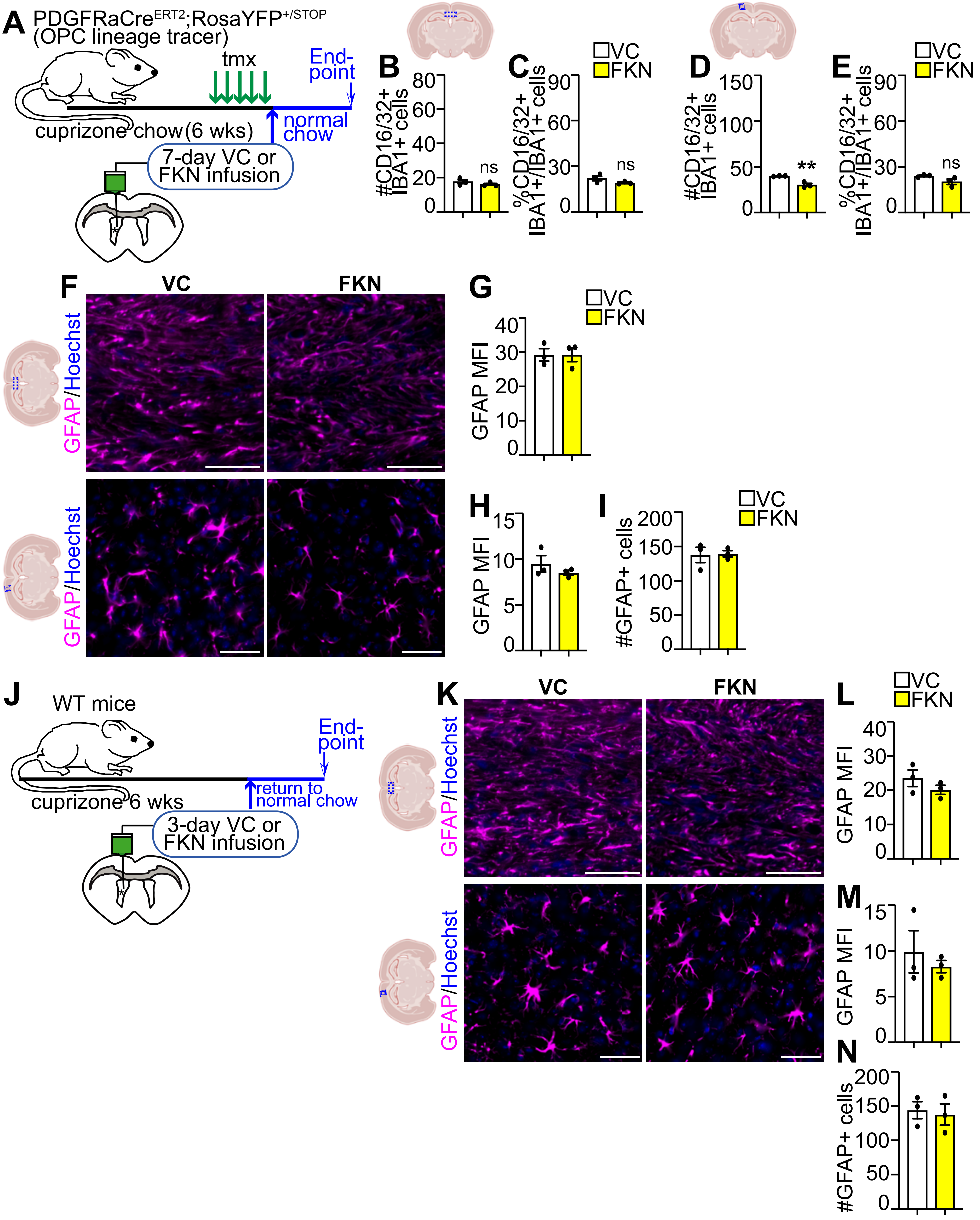
A
PDGFRaCre^ERT2;RosaYFP^+/STOP
(OPC lineage tracer)
tmx
End-point
normal chow
cuprizone chow (6 wks)
7-day VC or FKN infusion
B
#CD16/32+ IBA1+ cells
ns
C
%CD16/32+ IBA1+/IBA1+ cells
VC
FKN
D
#CD16/32+ IBA1+ cells
**
E
%CD16/32+ IBA1+/IBA1+ cells
F
VC
FKN
GFAP/Hoechst
G
GFAP MFI
H
GFAP MFI
I
#GFAP+ cells
J
WT mice
cuprizone 6 wks
return to normal chow
End-point
3-day VC or FKN infusion
K
VC
FKN
GFAP/Hoechst
L
GFAP MFI
M
GFAP MFI
N
#GFAP+ cells

Figure S4. Related to Figure 5. ***FKN does not affect astrocyte activation or density.*** **A-I.** Analysis of microglia and astrocyte activation in 7-day infused remyelinating mice. **A.** Schematic illustration of experiment. 10 week-old PDGFRαCre^{ERT2};RosaYFP$^{STOP/+}$ were demyelinated with cuprizone chow for 6 weeks. In the last week of cuprizone treatment, mice were injected with tamoxifen once daily for 5 days. 72 hours later, intracerebral ventricular (ICV) surgery was performed. FKN or VC was infused unilaterally into the lateral ventricle (indicated by asterisk) for 7 days via osmotic mini-pump. After ICV surgery, mice were returned to normal chow for recovery. **B-E.** Analysis of average number of CD16/32+IBA1+ cells (**B, D**) and the proportion of IBA1+CD16/32+ cells (%IBA1+CD16/32+/IBA1+ cells **C, E**) in midline corpus callosum (**B-C**) and cortical grey matter (**D-E**). Approximate location of analyzed images is depicted as blue rectangles in brain schematics. ** $p<0.01$, ns = not significant. n=3 mice per group from at least 2 independent litters. **F.** Representative images of midline corpus callosum (top row) and cortical grey matter (bottom row) immunostained for GFAP (magenta) and counterstained with Hoechst 33258 (blue) from VC- and FKN-infused mice. Approximate location is indicated by blue rectangle in the brain schematic. Scale bars: 50 µm. **G-H.** Analysis of **F** for mean fluorescence intensity (MFI) of GFAP signal in corpus callosum (**G**) and cortical grey matter (**H**). **I**. Quantification of **F** for average number of GFAP+ cells in cortical grey matter of VC and FKN-infused mice. n=3 mice per group from at least 2 independent litters. **J-N.** Analysis of astrocyte activation in 3-day infused remyelinating mice **J.** Schematic illustration of experiment. 10 week-old WT C57BL/6J mice were demyelinated with cuprizone-chow administration for 6 weeks. At this point, FKN or VC was infused unilaterally into the lateral ventricle (indicated by asterisk) for 3 days via osmotic mini-pump. After intracerebral ventricular (ICV) surgery, mice were returned to normal chow for recovery. **K**. Representative images of midline corpus callosum (top row) and cortical grey matter (bottom row) immunostained for GFAP (magenta) and counterstained with Hoechst 33258 (blue) from VC- and FKN-infused mice. Approximate location is indicated by blue rectangle in the brain schematic. Scale bars: 50 µm. **L-M.** Analysis of **K** for mean fluorescence intensity (MFI) of GFAP signal in corpus callosum (**L**) and cortical grey matter (**M**). **N**. Quantification of **K** for average number of GFAP+ cells in cortical grey matter of VC and FKN-infused mice. n=3 mice per group from at least 2 independent litters. Data were analyzed using unpaired t-test. All error bars represent SEM.

Figure S5

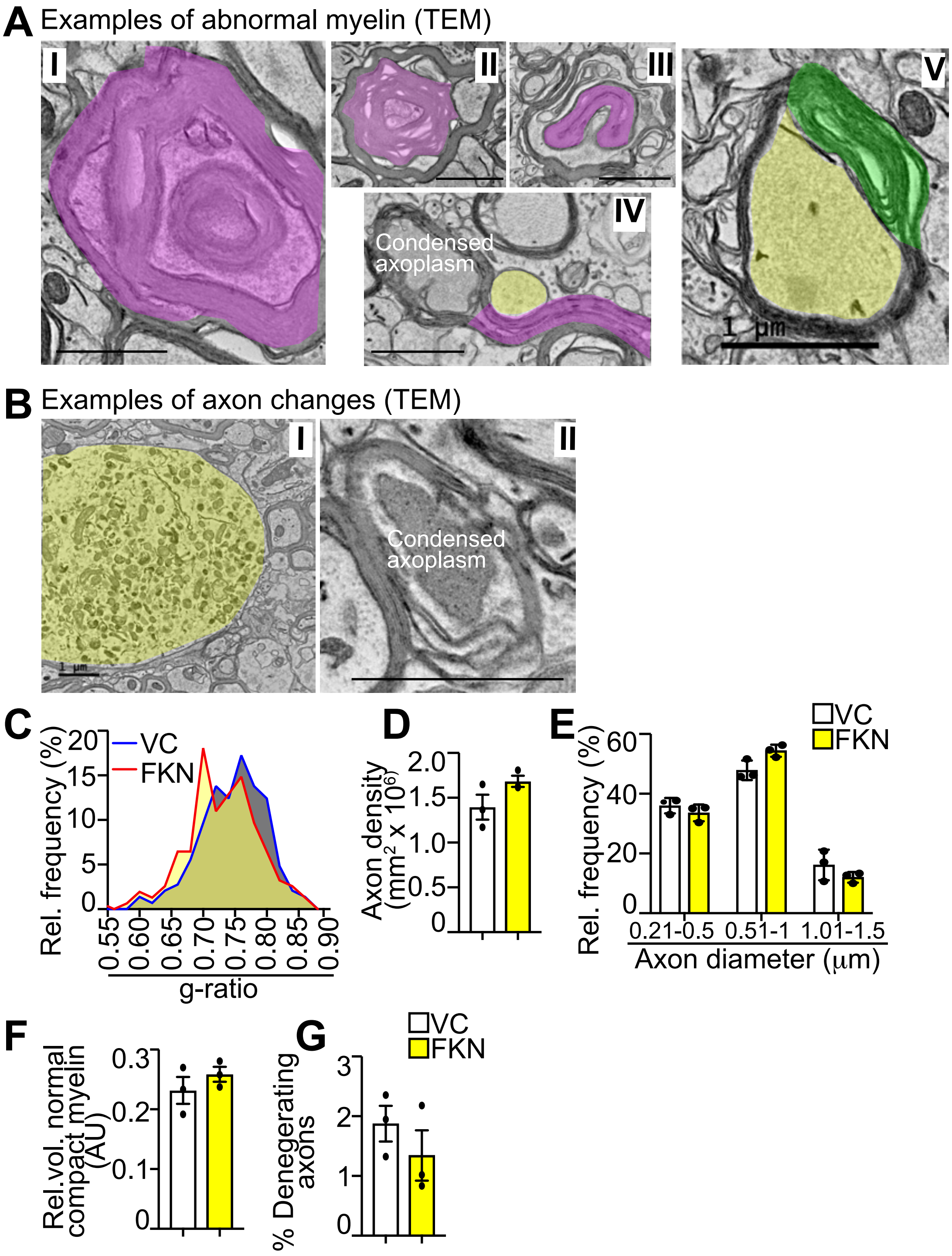
A Examples of abnormal myelin (TEM)
I
II
III
IV
V
Condensed axoplasm
1 μm
B Examples of axon changes (TEM)
I
II
Condensed axoplasm
1 μm
C
VC
FKN
Rel. frequency (%)
g-ratio
D
Axon density (mm² x 10⁶)
E
Rel. frequency (%)
Axon diameter (μm)
0.21-0.5
0.51-1
1.01-1.5
VC
FKN
F
Rel.vol. normal compact myelin (AU)
G
% Denegerating axons
VC
FKN

Figure S5. Related to Figure 6. ***Examples of abnormal myelin and axon changes.*** **A.** Representative TEM images from VC-infused (micrographs in I, III-V) or FKN-infused (micrograph in II) mice illustrating redundant (purple) and split (green) abnormal myelin. **B**. Representative TEM images from VC-infused (micrograph in I) or FKN-infused (micrograph in II) mice illustrating swollen (yellow with filled organelles) and condensed (dark grey) abnormal axoplasm. Scale bars: 1 µm. **C-E**. Analysis of Fig. 6B for g-ratio frequency distribution (**C**), average total axon density (**D**, each data point represents a mouse), relative axon frequency within 0.21-0.5, 0.51-1 and 1.01-1.5 µm diameter (**E**, each data point represents a mouse). **F-G**. Morphometric analysis of **A** for normal compact myelin (**F**, each data point represents a mouse) and of **B** for degenerating axons (**G**, each data point represents a mouse). n=3 mice per group from at least 2 independent litters. For g-ratio analysis, at least 100 axons from 8 images per sample were analyzed. For morphometric analysis, 30 images per sample were analyzed. For all bar graphs error bars represent SEM. Data were analyzed using unpaired t-test, except data in **E** were analyzed with multiple t-test.

Figure S6

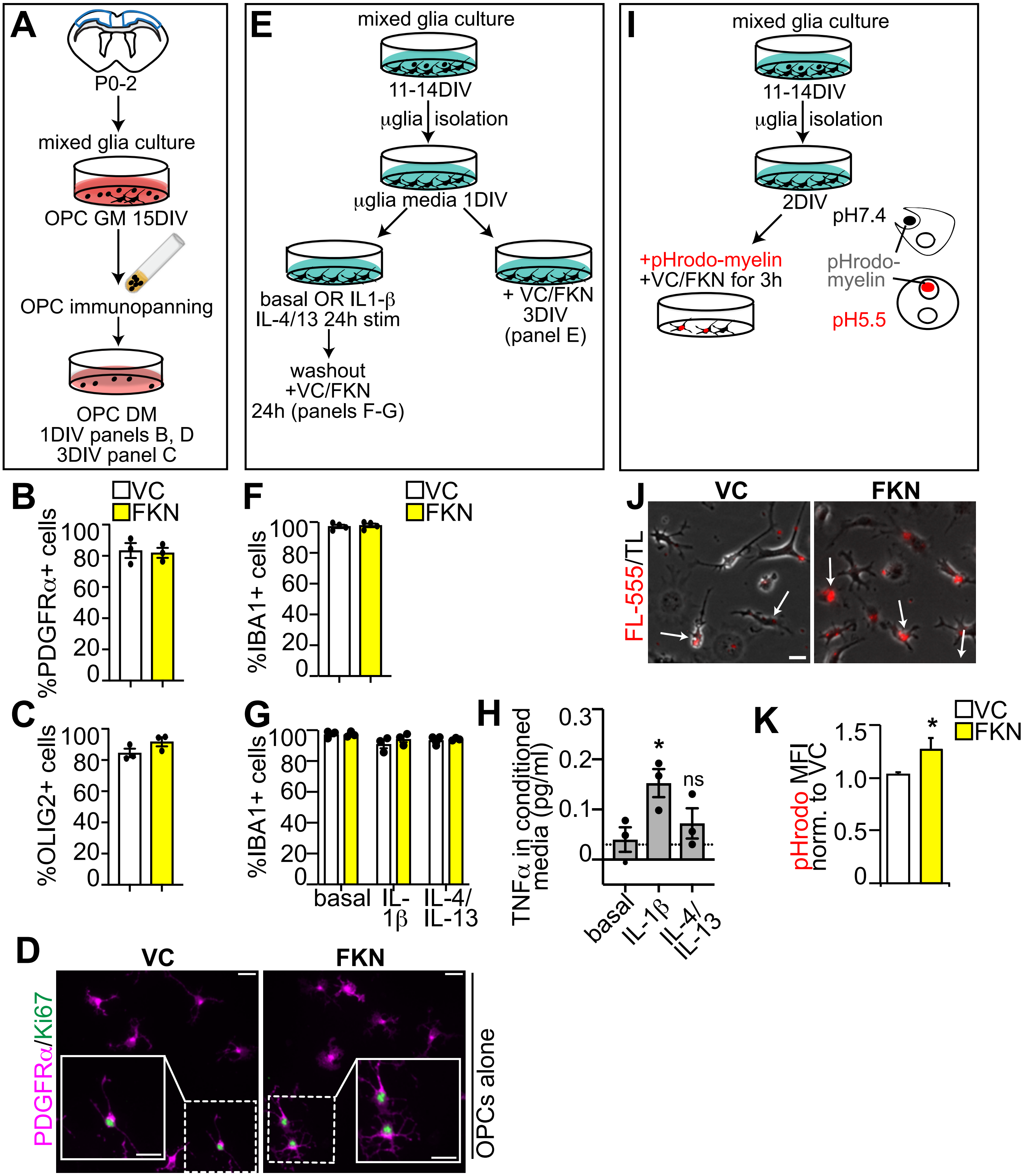
A
P0-2
mixed glia culture
OPC GM 15DIV
OPC immunopanning
OPC DM
1DIV panels B, D
3DIV panel C
E
mixed glia culture
11-14DIV
μglia isolation
μglia media 1DIV
basal OR IL1-β
IL-4/13 24h stim
washout
+VC/FKN
24h (panels F-G)
+ VC/FKN
3DIV
(panel E)
I
mixed glia culture
11-14DIV
μglia isolation
2DIV
+pHrodo-myelin
+VC/FKN for 3h
pH7.4
pHrodo-
myelin
pH5.5
B
VC
FKN
%PDGFRα+ cells
C
%OLIG2+ cells
D
VC
FKN
PDGFRα/Ki67
OPCs alone
F
VC
FKN
%IBA1+ cells
G
%IBA1+ cells
basal
IL-1β
IL-4/IL-13
H
TNFα in conditioned media (pg/ml)
ns
basal
IL-1β
IL-4/IL-13
J
VC
FKN
FL-555/TL
K
VC
FKN
pHrodo MFI norm. to VC

Figure S6. Related to Figure 7. ***FKN increases myelin debris phagocytosis by microglia in vitro.*** **A.** Schematic illustration of **B-D**. Cortices from P0-P2 CD1 pups were cultured as mixed glia in OPC GM (growth media). After 15 days OPC were isolated by immunopanning using PDGFRα+ magnetic beads (please see Experimental Procedures for more details). OPCs were cultured for 1-3 DIV in OPC DM (differentiation media). **B**. Quantification of % PDGFRα+ cells in OPC cultures treated with VC/FKN for 1DIV. n=3 biological replicates. **C**. Quantification of % OLIG2+ cells in OPC cultures treated with VC/FKN for 3DIV. n=3 biological replicates. **D**. Representative images of OPCs cultured in the absence of microglia and presence of VC or 250 ng/ml FKN for 1DIV and immunostained for PDGFRα (magenta) and Ki67 (green). Insets are shown at higher magnification and demonstrate PDGFRα+Ki67+ OPCs. Scale bar: 20 μm. **E.** Schematic illustration of **F-H**. Cortices from P0-P2 CD1 pups were cultured as mixed glia in microglia media. After 11-14 days, microglia were isolated by shaking. Microglia were then pre-stimulated with IL-1β and IL-4/IL-13 cytokines or no cytokines (basal conditions) for 24h and further treated with VC/FKN for additional 24h (results in panels **F-G**). Alternately, isolated microglia were cultured in microglia media with VC/FKN for 3DIV (results in panel **F**). **F**. Quantification of % IBA1+ cells in microglia cultures after 3DIV. n=3 biological replicates. **G**. Quantification of % IBA1+ cells in basal, IL-1β and IL-4/IL-13 pre-stimulated microglia cultures treated with VC or FKN for additional 24h. n=3 biological replicates. **H**. ELISA measurements of TNFα in media conditioned by basal, IL-1β and IL-4/IL-13 pre-stimulated microglia. Dashed line corresponds to media not exposed to microglia. Each data point corresponds to biological replicate. * $p<0.05$, ns = not significant. n=3 biological replicates. **I**. Schematic illustration of **J-K**. Cortices from P0-P2 CD1 pups were cultured as mixed glia in microglia media. After 11-14 days, microglia were isolated by shaking. Microglia were then cultured without FBS for 24h and further incubated with pHRodo-myelin and VC/FKN for additional 3h. **J**. Representative images of primary microglia (cells imaged with transmitted light [TL]) incubated with pHRodo-myelin (red fluorescence designated as FL-555) from VC- and FKN-treated cultures. Arrows designate microglia with pHRodo signal. Scale bars: 20 μm. **K**. Quantification of **J** for mean fluorescence intensity (MFI) of pHRodo signal normalized to VC. * $p<0.05$. n=4 biological replicates. Data were analyzed with paired t-test, except data in **G** were analyzed with multiple t-test and data in **H** were analyzed with one-way ANOVA followed by Dunnett's post-hoc test. All error bars represent SEM.

Supplemental Experimental Procedures

MATERIALS AND METHODS

Growth factors and cytokines: Murine soluble FKN (CX3CL1) was obtained from R&D Systems (cat #458-MF), FKN-647 from Almac (cat #CAF-51-A-01, #CAF-14-A-03). BSA-647 was obtained from ThermoFisher (cat# A34785). For primary cell culture experiments, FKN was reconstituted in sterile 1X PBS at a concentration of 100 µg/mL and used at 250 ng/ml. For intracerebral ventricular (ICV) infusions, FKN-647 was reconstituted at 0.5 mg/mL in 0.1% BSA in 1X PBS, and unconjugated FKN was reconstituted at 16.7 ng/mL for 3-7 day infusion or 75.8 ng/mL for 21-day infusion in sterile 0.2% bovine serum albumin (BSA, Jackson ImmunoResearch) in 1x PBS and infused at 200 ng/day rate using Alzet osmotic mini-pumps as per (Watson et al., 2021). Murine IL-1β was obtained from Cedarlane (cat# CLCYT273), reconstituted in cell culture grade H_2O supplemented with 0.1% BSA at a concentration of 10 µg/ml and used at 10 ng/ml; mouse IL-4 was obtained from Miltenyi Biotec (cat#130-097-761) and reconstituted in cell culture grade H_2O at 10 µg/ml; mouse IL-13 was purchased from R&D Systems (cat#413ML/CF) and resuspended in D-PBS at 50µg/ml, IL-4 and IL-13 were used at 20 ng/ml; GM-CSF (granulocyte-macrophage colony-stimulating factor) was obtained from R&D Systems (cat# 415ML/CF), reconstituted at 100 µg/ml and used at 10 ng/ml; FGF was obtained from Peprotech (cat #100-18B), reconstituted in cell culture grade H_2O at 10 µg/ml and used at 10 ng/ml; PDGF-AA was obtained from R&D (cat# 1055-AA-050), reconstituted in sterile 4 mM HCl at 10 µg/ml and used at 10 ng/ml.

Experimental Model And Subject Details

Mice: Animal use protocols were approved by the Research Ethics Office at the University of Alberta and the University of Ottawa in accordance with the Canadian Council of Animal Care Policies. Mice from both sexes were used for all cell culture and *in vivo* experiments. PDGFRαCre^{ERT2} (B6N.Cg-Tg(Pdgfra-cre/ERT)467Dbe/J; RRID:IMSR_JAX:018280), RosaYFP^{STOP} (B6.129X1-Gt(ROSA)26Sortm1(EYFP)Cos/J; RRID:IMSR_JAX:006148), Rosa$^{mT/mG}$ (B6.129(Cg)-Gt(ROSA)26Sortm4(ACTB-tdTomato,-EGFP)Luo/J; RRID:IMSR_JAX:007676) and wild-type (WT) C57BL/6J mice were obtained from Jackson Laboratories (Kang et al., 2010; Muzumdar et al., 2007; Srinivas et al., 2001). Experimental mice were generated by mating PDGFRαCre^{ERT2}-positive hemizygous male with homozygous RosaYFP^{STOP} or Rosa$^{mT/mG}$ females. PDGFRαCre^{ERT2}-positive progeny were used in lineage tracing experiments. For some demyelination experiments as well as for RNA scope, WT animals were used as indicated in the figure legends. CD1 mice purchased from Charles River Laboratory were used for all primary cell culture experiments (developmental age: postnatal day [P] 0-2). For genotyping, the following primers were used: PDGFRαCre^{ERT2}: TCAGCCTTAAGCTGGGACAT (5'-3', forward), ATGTTTAGCTGGCCCAAATG (5'-3', reverse), RosaYFP^{STOP}: AAAGTCGCTCTGAGTTGTTAT (5'-3', common), GGAGCGGGAGAAATGGATATG (5'-3', wildtype), AAGACCGCGAAGAGTTTGTC, (5'-3', mutant), Rosa$^{mT/mG}$: CTTTAAGCCTGCCCAGAAGA (5'-3', common), AGGGAGCTGCAGTGGAGTAG (5'-3', wildtype), TAGAGCTTGCGGAACCCTTC (5'-3', mutant).

Cuprizone-induced demyelination experiments: For cuprizone-induced demyelination, 10-week old WT, PDGFRαCre^{ERT2};RosaYFP^{STOP} or PDGFRαCre^{ERT2};Rosa$^{mT/mG}$ mice were subjected to nutragel (Bio-Serv) containing 0.2% cuprizone (bis-cyclohexanone oxaldihydrazone, Sigma) for 6 weeks as described in (Saito et al., 2021). Animal weight and health was monitored

weekly. After 6 weeks of cuprizone chow, intracerebral ventricular surgery (ICV) was performed (please see details below), at which point mice were returned to normal chow for 3-21 days to allow remyelination.

Tamoxifen injections: PDGFRαCre^{ERT2};RosaYFP^{STOP} or PDGFRαCre^{ERT2};mT/mG animals were injected with 3 mg tamoxifen dissolved in 10% ethanol (Commercial Alcohols) and 90% sunflower seed oil (Sigma) daily for 5 days during last week of cuprizone treatment. 72h after last tamoxifen injection, intracerebral ventricular surgery (ICV) was performed. Normal (non-demyelinated) 10-week old PDGFRαCre^{ERT2}; RosaYFP^{STOP} mice were injected with tamoxifen as above. 72h after last tamoxifen injection, mice were perfused as per details below.

ICV infusions: For FKN-647 and BSA-647 one-time injection experiments, 2-3-month old wild-type C57/BL6J mice were used. For FKN infusion experiments, we used wild-type C57/BL6J, PDGFRαCre^{ERT2};RosaYFP^{STOP} or PDGFRαCre^{ERT2};mT/mG demyelinated with cuprizone as described above. Mice were anesthetized via inhalation of isoflurane and placed in a stereotaxic frame. Syringe needle (for one-time injection) or cannulas (Alzet, for multi-day infusion) were positioned after craniotomy for infusion into right ventricle using the following coordinates relative to bregma: -1.000 medio-lateral (ML), -0.300 anterior-posterior (AP), -2.500 dorso-ventral (DV). For one-time injection, 0.5-1 µl of FKN-647 or matched volume and equimolar amount of BSA-647 were injected once over a 10-20 min period as described in (Watson et al., 2021). For multi-day infusion, 7-day or 28-day osmotic mini-pumps (Alzet, 1007D or 1004) were connected containing VC (vehicle-control: 0.2% BSA in 1x PBS) or 16.7 ng/mL or 75.8 ng/mL FKN in VC. Overall, 200 ng of FKN was delivered over every 24h for 3-21 days. Cannula was secured to the skull with Loctite 454. After surgery, mice were returned to normal chow. BrdU (5-bromo-2'-deoxyuridine, Sigma) was injected at 100 mg/kg dose 24h before perfusions.

Lysolethicin (LPC) injections: LPC-mediated demyelination was performed as described in (Kosaraju et al., 2020). Briefly, one-month old WT mice were injected with LPC (Sigma, L1381) (1 µl of 1% solution in 1xPBS) using a stereotactic apparatus at two sites: +1.0 mm AP, +1.0 mm ML, −2.2 mm DV and + 0.7 mm AP, +1.0ML, −2.2 DV. After injection, needle was held in place for 3 min to reduce back-flow. Animals were allowed to recover for 14 days.

Myelin isolation: Myelin was isolated as described in (Bhattacherjee et al., 2019). Briefly, brains from 5-month old Sprague Dawley rats were minced and homogenized in ice-cold 0.3 M sucrose. The homogenate was layered over 0.83 M sucrose and ultracentrifuged for 45 min at 75,000 g. The crude myelin was extracted, homogenized and ultracentrifuged twice more (12,000 g, 15 and 10 min, 4°C). Myelin was further purified by another density gradient and subjected to Tris·Cl buffer osmotic shock. Myelin was then resuspended in 100 mM $NaHCO_3$, pH 8.5 at 100 mg/ml concentration and incubated with 10 µM of pHRodo dye (Invitrogen) for 1h at room temperature. The excess dye was washed out by washing myelin in 1xPBS four times. The labeled myelin was stored in 1xPBS containing 1% DMSO as single-use aliquots at −80°C.

Tissue dissection and collection: Mice under 21 days of age were euthanized with CO_2. Dissected brains were kept in ice-cold Hank's Balanced Salt Solution (HBSS, Gibco) until dissections and subsequent primary cultures. Mice over 21 days of age were anesthetized with 102 mg/kg of body weight Euthansol (WDDC) and perfused transcardially with HBSS, followed by 4% paraformaldehyde in 1X PBS (PFA, Acros Organics). Dissected brains were incubated in 4% PFA at 4°C for additional 24 hours, after which brains were cryopreserved for 72 hours in 30% sucrose (Fisher) in 1X PBS. Brains were embedded in optimal cutting temperature (O.C.T) compound (Thermo Scientific™ Shandon™ Cryomatrix™) and flash frozen for downstream

assays. For transmission electron microscopy, animals were perfused with HBSS followed by 4% PFA in 0.1M Sodium Cacodylate buffer with 2 mM $CaCl_2$ (solution A). The brain was removed and post-fixed in solution A for 1 hour at RT, followed by 24h incubation at 4°C. Then, 1 mm slices were cut with sagittal mouse brain matrix (RWD), and region of interest dissected into 2.5 mm thickness pieces, which were transferred into 2% PFA, 2.5% Glutaraldehyde, 0.1M Sodium Cacodylate buffer with 2 mM $CaCl_2$. Samples were infiltrated with Spurr's resin and polymerized in 70°C oven with fresh resin for 24 h. Ultrathin 70 nm sections were generated using an ultramicrotome (Leica, EM UC6).

Primary cell cultures:

Microglia cultures: Microglial cells were expanded and isolated through a mixed glial culture as per (Galleguillos et al., 2022; Saura et al., 2003) with slight modifications. Briefly, cerebral cortices from P0-P2 CD1 brains were enzymatically digested and mechanically dissociated and cortical cells were seeded in DMEM/F12 (Dulbecco's Modified Eagle Medium, Ham's F-12, Hyclone) with 10% FBS (Fetal Bovine Serum, Sigma), 1% penicillin/streptomycin (Pen/Strep, Lonza), 1% sodium pyruvate (Invitrogen) and 50 μM β-mercaptoethanol (herein referred to as **microglia media**). Media was replaced twice a week. To increase the number of microglial cells, the culture was treated with 10 ng/ml GM-CSF (Granulocyte Macrophage Colony Stimulating Factor, R&D systems) on days 8-11, which was replaced with microglia media without penicillin/streptomycin the day before microglia isolation. On day 14, conditioned media was collected and filtered and microglial cells were isolated using 15 mM lidocaine (Sigma) and shaking the plate at 100 rpm for 10 min as described in (Galleguillos et al., 2022; Rabinovitch and DeStefano, 1975). Isolated microglial cells were plated at 47,000-52,000 cells/cm^2 density for 24h in 50% conditioned media and 50% microglia media for polarization, assessment of proliferation or for co-culture with OPCs as described below. For proliferation analysis, after 24h media was replaced to microglia media without β-mercaptoethanol and VC (1xPBS) or 250 ng/ml FKN in VC was added for 3 days. For co-cultures, isolated OPCs were plated on top of microglia as described below. For co-cultures with pre-treatment, isolated microglia were plated at 47,000-52,000 cells/cm2 density for 24h in 50% conditioned media and 50% microglia media for 24h, followed by 24h in microglia media without FBS but with VC (1xPBS) or 250ng/ml FKN. VC/FKN pre-treated cells were washed once with microglia media without FBS. Isolated pre-treated OPCs were plated on top of microglia as described below.

Cortical OPC cultures: Cerebral cortices from P0-P2 CD1 brains were dissociated as described above and cultured on plates pre-coated with 40 µg/ml Poly-D-Lysine (Sigma) and 4 µg/ml laminin (Corning) in Serum-Free Media (SFM: DMEM low glucose [Gibco], F12 [Gibco], 0.6% glucose [Sigma], 0.1125% $NaHCO_3$ [Gibco], 5 mM N-2-hydroxyethylpiperazine-N-2-ethane sulfonic acid [HEPES, Gibco], 100 µg/mL L-glutamine [Lonza], 1% Pen/Strep) supplemented with 2% B27 supplement (Life Technologies), 10 ng/ml FGF (Peprotech) and 10 ng/ml PDGF-AA (R&D) (herein referred to **OPC growth media [GM]**). Media was changed every 4 days. After 15 days, cells were lifted with accutase (Life Technologies) and subjected to immunopanning with PDGFRα-specific magnetic beads as per manufacturer's instructions (Miltenyi). Recovered cells were then seeded at 47,000 cells/cm^2 on top of pre-plated microglia or alone in SFM supplemented with 2% B27 and 40 ng/ml 3,3',5-Triiodo-L-thyronine (T3, Sigma) (herein referred to **OPC differentiation media [DM]**). Cells were incubated for an additional 1-3 days. For co-cultures with pre-treatment, OPCs were cultured for 15 days as described above, followed by treatment with VC (1xPBS) or 250 ng/mL FKN for 24h. Cells were then washed, lifted with accutase, isolated via immunopanning, and seeded on top of pre-plated pre-treated microglia and incubated for an additional 3 days in OPC DM as described above.

Microglia polarization: Isolated microglia were treated with polarizing cytokines as described in (Durafourt et al., 2012; Galleguillos et al., 2022; Krasnow et al., 2017). Briefly, microglia were plated on poly-D-lysine and laminin coated coverslips and incubated in 50% conditioned media/50% microglia media for 24h. At this time point, media was changed to microglia medium without FBS or β-mercaptoethanol containing no cytokines (basal conditions), 10 ng/ml IL-1β (Cedarlane) or 20 ng/ml IL-4 (Miltenyi) and IL-13 (R&D Systems). After 24h polarization, conditioned media was collected for ELISA (enzyme-linked immunosorbent assay) as specified below, and replaced with microglia media without FBS or β-mercaptoethanol and containing VC (1xPBS) or 250 ng/ml FKN dissolved in VC. 24h after VC/FKN treatment, cells were processed for downstream assays.

Myelin phagocytosis assays: Myelin labeled with pHRodo was added to microglia cultured in basal conditions at 1:1,000 concentration. VC (1x PBS) or 250 ng/ml FKN (dissolved in VC) was added at the same time as myelin debris. Cultures were incubated for 3h and imaged as described below.

Conditioned medium preparation/ELISA: microglia were cultured in the presence of no cytokines (basal conditions), IL-1β or IL-4 and IL-13 as described above. Conditioned medium was collected and centrifuged at 1000 g for 7 min to remove dead cells and debris. TNF-α protein detection was performed via ELISA kit (Invitrogen) following manufacturer's directions. Measurements were performed via technical duplicates from 3 independent preparations of microglia conditioned medium.

<u>Reagents and Immunostaining:</u>

Immunocytochemistry (ICC): Cell cultures were fixed and stained as described in (Watson et al., 2021). Briefly, adherent cell cultures were fixed with 4% PFA for 10 minutes at room temperature, permeabilized with 0.2% NP-40 (Sigma) for 5 min, and blocked with 6% donkey serum (Jackson ImmunoResearch) in 0.5% BSA in 1X PBS. Primary antibodies were added for overnight at 4°C, and appropriate secondary antibodies were added for 1 hour at room temperature (list of antibodies is in the "Antibodies" section below). Nuclei were counter-stained with Hoechst 33258 (Riodel-De Haen Ag). Slides were mounted with Fluoromount G (ThermoFisher).

Immunohistochemistry (IHC): Cryopreserved brains were sectioned at 18 μm and stained as described in (Watson et al., 2021). For FKN-647 diffusion assay, sections were rehydrated in 1XPBS, immediately counterstained with Hoechst 33258 to visualize nuclei and mounted with Fluoromount G. For all other staining, sections were rehydrated in 1X PBS and then permeabilized and blocked with 5% BSA and 0.3% Triton-X100 in 1X PBS for 1 hour at room temperature. Tissue sections were incubated overnight at 4°C with appropriate primary antibodies listed in "Antibodies" section diluted in 5% BSA in 1X PBS. Mouse on mouse (MOM) kit was used for primary antibodies raised in mouse, in accordance with manufacturer's instructions (VectorLabs). Appropriate secondary antibodies listed in "Antibodies" section were added for 1h at room temperature. Nuclei were counterstained with Hoechst 33258. Sections were mounted with Fluoromount G.

Staining with FluoroMyelin was performed as per manufacturer's instructions (Invitrogen). Briefly, sections were rehydrated in 1X PBS and then permeabilized and blocked with PBS and 0.2% Triton-X100 in 1X PBS for 25 min at room temperature. Stock FluoroMyelin was diluted 300X in 1X PBS and added to sections for 20 min at room temperature. Nuclei were counterstained with Hoechst 33258. Sections were mounted with Fluoromount G.

Antibodies: Mouse anti-APC/CC1 (1:300, Calbiochem, RRID:AB_2057371), sheep anti-BrdU (Abcam, 1:1000, ICC, RRID:AB_302659), rabbit anti-GFAP (Dako, 1:1000, immunostaining, RRID: AB_10013382), rat anti-GFAP (Thermo Fisher, 1;1000, RRID:AB_2532994), chicken anti-eGFP (Abcam, 1:1000, IHC, RRID: AB_300798), goat anti-IBA1 (Novus Bio, 1:300 for IHC, 1:1000 for ICC, RRID:AB_521594), rabbit anti-IBA1 (Wako, 1:1000, RRID: AB_839504); mouse anti-Ki67 (BD Pharmingen, 1:300, ICC, RRID: AB_396287), rat anti-MBP (a. a. 82-87) (Millipore, 1:500, ICC, RRID: AB_94975), rabbit anti-Olig2 (Millipore, 1:1000 for IHC, 1:2000 for ICC, immunostaining, RRID: AB_570666), mouse anti-Olig2 (Millipore, 1:1000 for IHC, 1:500 for ICC, RRID: AB_10807410), goat anti-PDGFRα (R&D Systems, 1:400 for IHC, 1:300 for ICC, RRID: AB_2236897), rat anti-CD16/32 (Tonbo Biosciences, 1:400 for IHC, 1:400 for ICC, RRID:AB_2621487), rabbit anti-DCX (Abcam, 1:1,000 for IHC, RRID:AB_732011). Fluorescently labeled highly cross-absorbed secondary antibodies (Jackson ImmunoResearch) were used at 1:1000 dilution. If MOM kit was used, Cy3-, DTAF-, or Cy5 conjugated streptavidin (Jackson ImmunoResearch) were used at 1:1000 dilution.

RNA scope: Brain cryosections from WT demyelinated mice were subjected to RNA scope as described in (Voronova et al., 2017; Watson et al., 2021) with probes targeting murine *Cx3cr1* and/or *Pdgfrα* mRNA or negative control probe purchased from Advanced Cell Diagnostics according to the manufacturer's instructions. Briefly, 18 μm sections were dehydrated with ethanol and rehydrated with protease (RNA scope kit) diluted 1:5 in 1xPBS for 10 min at 37°C. For RNA scope-IHC combination, sections were rehydrated with protease diluted 1:10 in 1xPBS for 10 min at 37°C to preserve protein epitopes. Protease-treated sections were washed with wash buffer provided by the RNA scope kit and incubated with probes for 2h at 37°C. Sections were then washed again and incubated with signal amplification solutions as per the manufacturer's protocol. At this point, sections were either counter-stained with DAPI (provided in the RNA scope kit) and mounted, or processed for IHC. For latter, RNA scope-processed sections were post-fixed in 4% PFA for 10 min at room temperature. After extensive washes with 1xPBS, sections were permeabilized, blocked and stained with anti-IBA1 antibodies as described in the IHC section.

Microscopy: Brain sections were imaged with Zeiss LSM700 confocal microscope with photomultiplier tube (PMT) with 40X objective, where high magnification digital image acquisition was performed with 2-5x digital Zoom using Zen (Zeiss) (Figs. 1, 2, 3D), or with Olympus IX81 fluorescence microscope equipped with Okogawa CSU X1 spinning disk confocal scan head, 20x objective and a Hamamatsu camera (Hamamatsu), where digital image acquisition was performed with Volocity (Perkin Elmer) software (Fig. S1E). Z-stacks spanning 6-10 μm were taken with optical slice thickness 0.2-0.5 μm and stacked images or orthogonal sections through 3D projections are shown.

Images from all fixed primary cell culture experiments as well as *in vivo* experiments in Figs. 3K, 4-5, 6M-N, S1C-D, S3, S4 were captured with 20X objective using Zeiss Axio Imager M2 fluorescence microscope, ORCA-Flash LT sCMOS Camera and the Zen software (Zeiss). Images were captured in single plane except images in Fig. 6M-N were captured using Z-stacks with optical thickness of 0.5 μm spanning 5-8 μm.

Live microglia cultures were imaged using an inverted Zeiss Axio Observer Z1 microscope equipped with Axiocam 503 Mono camera and 20x objective. Five random pictures per well were taken with transmitted light and red excitation filter.

For electron microscopy, ultrathin 70 nm sections were cut from two blocks per sample and imaged under a transmission electron microscope (JEOL JEM-2100, Gatan Orius camera with Digital micrograph) at 200kV acceleration voltage.

RNA isolation and qRT-PCR: Total RNA from primary microglia was purified using Omega Biotek E.Z.N.A. microelute kit as per manufacturer's instructions. 50-80 ng of purified total RNA was reverse transcribed to generate cDNA using QuantiTect Reverse Transcription Kit (Qiagen) as described in (Voronova et al., 2017). One-fortieth of RT reaction was used as a template for qPCR amplification using the Ssoadvanced SYBR Green kit (Biorad) and the following primers: Cx3cr1 (Forward: CGGCCATCTTAGTGGCGTC and Reverse: GGATGTTGACTTCCGAGTTGC), housekeeping genes Hnrnpab (Forward: AGGACGCGGGAAAAATGTTC and Reverse: CAGTCAACAACCTCTCCAAACT) and β-actin (Forward: GGCTGTATTCCCCTCCATCG and Reverse: CCAGTTGGTAACAATGCCATGT). Data were acquired using Eppendorf Realplex2 (Eppendorf) instrument in technical duplicates and from three biological replicates. Data were normalized to β-actin and Hnrnpab as recommended in (Tanaka et al., 2017) and analyzed using $2^{-\Delta\Delta Ct}$ method as in (Livak and Schmittgen, 2001). Basal PBS treated microglia samples were used as a calibrator (equated to 1). All primers were validated, and qPCR assays were performed in accordance with Minimum Information for Publication of Quantitative Real- Time PCR Experiments guidelines (Bustin et al., 2009).

QUANTIFICATION, CO-LOCALIZATION AND STATISTICAL ANALYSIS

Cell cultures were analyzed with a Zeiss Axioimager fluorescence microscope. Digital image acquisition was performed with ZEN (Zeiss) software. In all culture experiments, 5-10 fields of view were captured with a 20X objective. At least 250 cells from each treatment and biological experiment were counted. Proliferation index is presented as %Ki67-positive, marker-positive cells over total marker-positive cells. *In vitro* data (ICC and q-PCR) are presented from at least 3 independent biological experiments.

For all *in vivo* experiments, midline corpus callosum or cortical grey matter in medial sections was imaged using 20X objective as indicated in figures, with exception of Fig. 3E-F, I-J, where rostral and caudal corpus callosum was imaged. Areas of interest were identified with Hoechst staining. For PDGFRαCre^{ERT2};RosaYFP^{STOP} mice, the results are presented as percentage of marker-positive, YFP-positive cells relative to total YFP-positive cells. For other quantifications, average # marker+ cells per field of view or defined cortical column is presented. Proliferative index is presented as % marker+BrdU+/marker+ cells. 5 matched sections per brain were analyzed from 6 mice across 3 independent litters. At least 300 cells per corpus callosum and 800 cells per cortical column in every sample were counted. *In vivo* analysis was performed in a blind fashion by two independent observers.

For co-localization analysis, cortical grey matter was imaged using 20X objective and captured using Z-stacks with an optical thickness of 0.5 μm spanning 5-8 μm. At least 4 anatomically matched images per mouse, from 3 mice per treatment group from at least two independent litters were analyzed. Z-stacks were then subjected to Imaris software (Imaris64 9.3.0) and voxel-based MBP/mGFP co-localization analysis was performed. Colocalization was calculated within the region of interest (ROI) and determined by the intensity threshold. % of ROI co-localized was normalized to VC.

Electron microscopy images were analyzed for g-ratio and morphometric parameters as described in (Bando et al., 2015; Edgar et al., 2010; Edgar et al., 2009; Edgar et al., 2020). In brief, the g-ratio was measured for myelinated axons, where axon and compact myelin morphology were normal as described in (Bando et al., 2015). The following axons were excluded from g-ratio analysis: axons with abnormal myelin morphology (e.g. decompacted, redundant and splitting myelin, see Fig. S5A) and axons that showed signs of degradation or swelling (see Fig. S5B) (Bando et al., 2015). On average, at least 8 images and 100 axons per sample were analyzed. Axonal and myelin abnormalities were separately measured using

morphometric analysis as described in (Edgar et al., 2020; Schäffner et al., 2021). Briefly, relative proportion of normal and abnormal myelin was determined by manually counting the number of intersections of normal and abnormal myelin with 64 equidistant grids overlaid in Fiji software. To determine the proportion of degenerating (e.g. axons with darkened axoplasm or swollen axons with organelle accumulation) or myelinated/unmyelinated axons, axons identified based on microtubules and neurofilaments were counted within a region of interest (ROI) superimposed onto the micrographs. Axonal density was analyzed using axons that fit within the ROI is presented as number of total or myelinated axons/mm^2. Proportion of degenerating or myelinated axons is presented as % of total axons or as total axons binned by axonal diameter. For axonal density and morphometric analyses, at least 30 images per sample were analyzed.

Images were counted in Zen or Fiji software (Schindelin et al., 2012) in a blinded fashion. Representative images were processed in Photoshop CC 2015 and figures in Adobe Illustrator CC 2015. Biorender was used to generate brain schematics.

Sample sizes (n) indicated in the figure legends 2-7 correspond to the number of biological replicates analyzed. Data in Fig. 1 and S1 is presented from two mice per treatment. All data are presented as mean ± SEM or as connected lines, where each line corresponds to one biological replicate.

All data were subjected to normality tests with D'Agostino & Pearson, Shapiro-Wilk and Kolmogorov-Smirnov tests and were considered normal. For two group comparisons, two-tailed paired student's t-tests (*in vitro* datasets) or two-tailed unpaired student's t-tests (for *in vivo* datasets) or multiple t-tests were used to assess statistical significance between means, where a p-value <0.05 was considered significant. For three or more group comparisons one-way or two-way ANOVA followed by Dunnett's or Tukey multiple comparisons tests were used. For comparing slopes of g-ratios with axon diameters, simple linear regression was applied where 95% CI of the best-fit line was used. In all cases, Prism (version 8.0.2) was used. Number of experiments and statistical information are stated in the corresponding figure legends. In figures, asterisks denote statistical significance marked by *, $p < 0.05$; **, $p < 0.01$; ***, $p < 0.001$.

References:

Bando, Y., Nomura, T., Bochimoto, H., Murakami, K., Tanaka, T., Watanabe, T., and Yoshida, S. (2015). Abnormal morphology of myelin and axon pathology in murine models of multiple sclerosis. Neurochemistry international 81, 16-27, https://doi.org/10.1016/j.neuint.2015.01.002.

Bhattacherjee, A., Rodrigues, E., Jung, J., Luzentales-Simpson, M., Enterina, J.R., Galleguillos, D., St Laurent, C.D., Nakhaei-Nejad, M., Fuchsberger, F.F., Streith, L., *et al.* (2019). Repression of phagocytosis by human CD33 is not conserved with mouse CD33. Communications biology 2, 450, https://doi.org/10.1038/s42003-019-0698-6.

Bustin, S.A., Benes, V., Garson, J.A., Hellemans, J., Huggett, J., Kubista, M., Mueller, R., Nolan, T., Pfaffl, M.W., Shipley, G.L., *et al.* (2009). The MIQE guidelines: minimum information for publication of quantitative real-time PCR experiments. Clin Chem 55, 611-622, https://doi.org/10.1373/clinchem.2008.112797.

Durafourt, B.A., Moore, C.S., Zammit, D.A., Johnson, T.A., Zaguia, F., Guiot, M.C., Bar-Or, A., and Antel, J.P. (2012). Comparison of polarization properties of human adult microglia and blood-derived macrophages. Glia 60, 717-727, https://doi.org/10.1002/glia.22298.

Edgar, J.M., McCulloch, M.C., Montague, P., Brown, A.M., Thilemann, S., Pratola, L., Gruenenfelder, F.I., Griffiths, I.R., and Nave, K.A. (2010). Demyelination and axonal

preservation in a transgenic mouse model of Pelizaeus-Merzbacher disease. EMBO Mol Med 2, 42-50, https://doi.org/10.1002/emmm.200900057.

Edgar, J.M., McLaughlin, M., Werner, H.B., McCulloch, M.C., Barrie, J.A., Brown, A., Faichney, A.B., Snaidero, N., Nave, K.A., and Griffiths, I.R. (2009). Early ultrastructural defects of axons and axon-glia junctions in mice lacking expression of Cnp1. Glia 57, 1815-1824, https://doi.org/10.1002/glia.20893.

Edgar, J.M., Smith, R.S., and Duncan, I.D. (2020). Transmission Electron Microscopy and Morphometry of the CNS White Matter. Methods Mol Biol 2143, 233-261, https://doi.org/10.1007/978-1-0716-0585-1_18.

Galleguillos, D., Wang, Q., Steinberg, N., Zaidi, A., Shrivastava, G., Dhami, K., Daskhan, G.C., Schmidt, E.N., Dworsky-Fried, Z., Giuliani, F., *et al.* (2022). Anti-inflammatory role of GM1 and other gangliosides on microglia. J Neuroinflammation 19, 9, https://doi.org/10.1186/s12974-021-02374-x.

Jäkel, S., Agirre, E., Mendanha Falcão, A., van Bruggen, D., Lee, K.W., Knuesel, I., Malhotra, D., ffrench-Constant, C., Williams, A., and Castelo-Branco, G. (2019). Altered human oligodendrocyte heterogeneity in multiple sclerosis. Nature 566, 543-547, https://doi.org/10.1038/s41586-019-0903-2.

Kang, S.H., Fukaya, M., Yang, J.K., Rothstein, J.D., and Bergles, D.E. (2010). NG2+ CNS glial progenitors remain committed to the oligodendrocyte lineage in postnatal life and following neurodegeneration. Neuron 68, 668-681, https://doi.org/10.1016/j.neuron.2010.09.009.

Kosaraju, J., Seegobin, M., Gouveia, A., Syal, C., Sarma, S.N., Lu, K.J., Ilin, J., He, L., Wondisford, F.E., Lagace, D., *et al.* (2020). Metformin promotes CNS remyelination and improves social interaction following focal demyelination through CBP Ser436 phosphorylation. Exp Neurol 334, 113454, https://doi.org/10.1016/j.expneurol.2020.113454.

Krasnow, S.M., Knoll, J.G., Verghese, S.C., Levasseur, P.R., and Marks, D.L. (2017). Amplification and propagation of interleukin-1β signaling by murine brain endothelial and glial cells. J Neuroinflammation 14, 133, https://doi.org/10.1186/s12974-017-0908-4.

Livak, K.J., and Schmittgen, T.D. (2001). Analysis of relative gene expression data using real-time quantitative PCR and the 2(-Delta Delta C(T)) Method. Methods 25, 402-408, https://doi.org/10.1006/meth.2001.1262.

Muzumdar, M.D., Tasic, B., Miyamichi, K., Li, L., and Luo, L. (2007). A global double-fluorescent Cre reporter mouse. Genesis 45, 593-605, https://doi.org/10.1002/dvg.20335.

Rabinovitch, M., and DeStefano, M.J. (1975). Use of the local anesthetic lidocaine for cell harvesting and subcultivation. In vitro 11, 379-381, https://doi.org/10.1007/bf02616374.

Saito, L.B., Fernandes, J.P., Smith, M.J., Doan, M.A.L., Branton, W.G., Schmitt, L.M., Wuest, M., Monaco, M.C., Major, E.O., Wuest, F., *et al.* (2021). Intranasal anti-caspase-1 therapy preserves myelin and glucose metabolism in a model of progressive multiple sclerosis. Glia 69, 216-229, https://doi.org/10.1002/glia.23896.

Saura, J., Tusell, J.M., and Serratosa, J. (2003). High-yield isolation of murine microglia by mild trypsinization. Glia 44, 183-189, https://doi.org/10.1002/glia.10274.

Schäffner, E., Edgar, J., Lehning, M., Strauß, J., Bosch-Queralt, M., Wieghofer, P., Berghoff, S., Krueger, M., Morawski, M., Reinert, T., *et al.* (2021). Myelin insulation as a risk factor for axonal degeneration in autoimmune demyelinating disease. bioRxiv, 2021.2011.2011.468223, https://doi.org/10.1101/2021.11.11.468223.

Schindelin, J., Arganda-Carreras, I., Frise, E., Kaynig, V., Longair, M., Pietzsch, T., Preibisch, S., Rueden, C., Saalfeld, S., Schmid, B., *et al.* (2012). Fiji: an open-source platform for biological-image analysis. Nature Methods 9, 676-682, https://doi.org/10.1038/nmeth.2019.

Srinivas, S., Watanabe, T., Lin, C.S., William, C.M., Tanabe, Y., Jessell, T.M., and Costantini, F. (2001). Cre reporter strains produced by targeted insertion of EYFP and ECFP into the ROSA26 locus. BMC Dev Biol 1, 4, https://doi.org/10.1186/1471-213x-1-4.

Tanaka, A., To, J., O'Brien, B., Donnelly, S., and Lund, M. (2017). Selection of reliable reference genes for the normalisation of gene expression levels following time course LPS stimulation of murine bone marrow derived macrophages. BMC immunology 18, 43, https://doi.org/10.1186/s12865-017-0223-y.

Voronova, A., Yuzwa, S.A., Wang, B.S., Zahr, S., Syal, C., Wang, J., Kaplan, D.R., and Miller, F.D. (2017). Migrating Interneurons Secrete Fractalkine to Promote Oligodendrocyte Formation in the Developing Mammalian Brain. Neuron 94, 500-516.e509, https://doi.org/10.1016/j.neuron.2017.04.018.

Watson, A.E.S., de Almeida, M.M.A., Dittmann, N.L., Li, Y., Torabi, P., Footz, T., Vetere, G., Galleguillos, D., Sipione, S., Cardona, A.E., *et al.* (2021). Fractalkine signaling regulates oligodendroglial cell genesis from SVZ precursor cells. Stem Cell Reports 16, 1968-1984, https://doi.org/10.1016/j.stemcr.2021.06.010.
