# Supplementary material for: Fractalkine enhances oligodendrocyte regeneration and remyelination in a demyelination mouse model
Source: Stem Cell Reports. 2023 Jan 5;18(2):519–33. doi: 10.1016/j.stemcr.2022.12.001 (PMC9968989; doi:10.1016/j.stemcr.2022.12.001)
Supplement: Document S2. Article plus supplemental information [file mmc2.pdf]

Stem Cell Reports
Article
OPEN ACCESS

Fractalkine enhances oligodendrocyte regeneration and remyelination in a demyelination mouse model

Monique M.A. de Almeida,[1,3,11] Adrianne E.S. Watson,[1,2,11] Sana Bibi,[1] Nicole L. Dittmann,[1,3] Kara Goodkey,[1,2] Pedram Sharafodinzadeh,[1] Danny Galleguillos,[3,4] Maryam Nakhaei-Nejad,[5] Jayasankar Kosaraju,[6] Noam Steinberg,[3,4] Beatrix S. Wang,[1,2] Tim Footz,[1] Fabrizio Giuliani,[5,10] Jing Wang,[6,7] Simonetta Sipione,[3,4] Julia M. Edgar,[8] and Anastassia Voronova[1,2,3,9,10,*]

[1]Department of Medical Genetics, Faculty of Medicine & Dentistry, University of Alberta, Edmonton, AB T6G 2H7, Canada
[2]Women and Children's Health Research Institute, University of Alberta, 5-083 Edmonton Clinic Health Academy, 11405 87 Avenue NW, Edmonton, AB T6G 1C9, Canada
[3]Neuroscience and Mental Health Institute, Faculty of Medicine & Dentistry, Edmonton, AB T6G 2E1, Canada
[4]Department of Pharmacology, Faculty of Medicine & Dentistry, Edmonton, AB T6G 2H7, Canada
[5]Department of Medicine, Faculty of Medicine & Dentistry, Edmonton, AB T6G 2H7, Canada
[6]Regenerative Medicine Program, Ottawa Hospital Research Institute, Ottawa, ON K1H 8L6, Canada
[7]Department of Cellular and Molecular Medicine, Faculty of Medicine, University of Ottawa Brain and Mind Research Institute, Ottawa, ON K1H 8M5, Canada
[8]School of Infection and Immunity, College of Medical Veterinary and Life Sciences, University of Glasgow, Glasgow G12 8TA, UK
[9]Department of Cell Biology, Faculty of Medicine & Dentistry, Edmonton, AB T6G 2H7, Canada
[10]Multiple Sclerosis Centre and Department of Cell Biology, Faculty of Medicine & Dentistry, Edmonton, AB T6G 2H7, Canada
[11]These authors contributed equally
*Correspondence: voronova@ualberta.ca
https://doi.org/10.1016/j.stemcr.2022.12.001

SUMMARY

Demyelinating disorders of the central nervous system (CNS) occur when myelin and oligodendrocytes are damaged or lost. Remyelination and regeneration of oligodendrocytes can be achieved from endogenous oligodendrocyte precursor cells (OPCs) that reside in the adult CNS tissue. Using a cuprizone mouse model of demyelination, we show that infusion of fractalkine (CX3CL1) into the demyelinated murine brain increases *de novo* oligodendrocyte formation and enhances remyelination in the corpus callosum and cortical gray matter. This is achieved by increased OPC proliferation in the cortical gray matter as well as OPC differentiation and attenuation of microglia/macrophage activation both in corpus callosum and cortical gray matter. Finally, we show that activated OPCs and microglia/macrophages express fractalkine receptor CX3CR1 *in vivo*, and that in OPC-microglia co-cultures fractalkine increases *in vitro* oligodendrocyte differentiation by modulating both OPC and microglia biology. Our results demonstrate a novel pro-regenerative role of fractalkine in a demyelinating mouse model.

INTRODUCTION

Multiple sclerosis (MS) is an autoimmune demyelinating disorder where myelin and the myelin-producing cells, oligodendrocytes, are damaged or lost in the central nervous system (CNS). There are currently no approved treatments that promote CNS remyelination, which are hypothesized to halt disease progression or enable neurological repair (Göttle et al., 2019). Remyelination can be achieved through *de novo* oligodendrocyte formation from adult parenchymal oligodendrocyte precursor cells (OPCs). When demyelination occurs, OPCs proliferate and migrate to the lesion site, where they differentiate into oligodendrocytes that remyelinate the lesion (McMurran et al., 2016). However, in people with MS, this process is highly inefficient (Yeung et al., 2019).

OPC fates, such as survival, proliferation, and differentiation, are important for efficient oligodendrocyte generation and are regulated by neighboring cells, such as microglia (Miron et al., 2013; Nemes-Baran et al., 2020; Sherafat et al., 2021b). While microglia can adopt a wide array of activation states, in general microglia can be either detrimental to regeneration through the release of pro-inflammatory cytokines, or beneficial by clearing the myelin debris and/or secreting anti-inflammatory and pro-oligodendrogenic proteins (Heß et al., 2020; Miron et al., 2013; Sherafat et al., 2021b).

OPCs are also regulated by chemokines, like neuronally secreted fractalkine (CX3CL1/FKN) (Watson et al., 2020). FKN signals through its sole receptor, CX3CR1, which is expressed at high levels in microglia and at lower levels in OPCs (Voronova et al., 2017; Watson et al., 2021). While the role of FKN signaling in adult parenchymal OPCs is unknown, we have shown that mice with constitutive Cx3cr1 KO (knockout), or with reduced levels of Cx3cr1 in cortical progenitors, have decreased developmental oligodendrocyte genesis (Voronova et al., 2017). Moreover, demyelinated Cx3cr1 KO mice and/or mice that express a human MS-associated *CX3CR1* variant display poor remyelination, impaired microglial phagocytosis, and decreased OPC

This is an open access article under the CC BY license (http://creativecommons.org/licenses/by/4.0/).

migration and proliferation (Cardona et al., 2018; Huang et al., 2006; Lampron et al., 2015; Mendiola et al., 2022). These reports suggest that FKN-CX3CR1 signaling is a critical regulator of oligodendrogenesis and myelination, and that activation of this pathway may be beneficial for remyelination. Whether exogenous FKN can increase oligodendrocyte and myelin regeneration from adult parenchymal OPCs remains to be addressed.

We show exogenous FKN infusion into the brain after cuprizone-induced demyelinating injury increases *de novo* oligodendrocyte formation and remyelination from parenchymal OPCs and attenuates microglia activation *in vivo*. In co-culture conditions, both OPCs and microglia need to be stimulated with FKN to elicit an increase in oligodendrocyte differentiation, suggesting that FKN regulates both OPC and microglia biology for a pro-oligodendrogenic response.

RESULTS

Cx3cr1 is expressed in OPCs and microglia in demyelinated CNS

Analysis of a single nuclei RNA sequencing dataset from MS patient brain white matter lesions showed *CX3CR1* is expressed in committed OPCs (cOPCs), immune oligodendrocytes (ImOLGS), and in microglia/macrophages (Jäkel et al., 2019) (Figure S1A). To probe *Cx3cr1* mRNA expression in the murine demyelinated brain, we subjected 10-week-old wild-type (WT) mice to a 6-week cuprizone chow treatment, which leads to corpus callosum (CC) and cortical gray matter (GM) demyelination (Baxi et al., 2017) (Figures 1A, S1B and S1C). RNA scope showed *Cx3cr1* mRNA was detected in *Pdgfrα*+ OPCs and IBA1+ microglia/macrophages in demyelinated midline CC and cortical GM (Figures 1B–1E). We corroborated these results in CC focally demyelinated with lysolethicin (Figures S1D and S1E). Thus, OPCs and microglia/macrophages express *Cx3cr1* in the demyelinated CNS and are poised to respond to FKN.

Exogenous FKN increases *de novo* oligodendrocyte genesis in remyelinating CC and cortical GM

To test whether FKN increases oligodendrocyte production after a demyelinating injury, cuprizone-treated WT mice were subjected to intracerebroventricular (ICV) surgery and 3-day VC/FKN infusion. After surgery, mice were returned to normal chow to allow remyelination (Figure 2A). We focused our analysis on medial brain areas, as they are remyelinated by parenchymal OPCs (Xing et al., 2014). At 3 days of infusion, the number and proportion of cells that expressed both OLIG2 (marker of oligodendroglial cells [OPCs and oligodendrocytes]) and CC1 (marker of mature oligodendrocytes), was increased ~1.46- to 2-fold in the CC and cortical GM in FKN-infused mice when compared with VC (Figures 2B–2G, and S2).

Intriguingly, we detected punctate OLIG2 signal specifically in the CC of infused animals that were not associated with Hoechst staining. These OLIG2 puncta showed colocalization with myelin basic protein (MBP) and were engulfed by IBA1+ microglia/macrophages (Figure 2H), suggesting that these puncta represent degenerated oligodendrocyte material. Notably, both the number of OLIG2+MBP+ puncta, and the proportion of IBA1+ cells engulfing OLIG2+MBP+ puncta were decreased in FKN-infused animals when compared with VC (Figures 2I and 2J). These data support and expand prior reports, which demonstrate tissue-protective properties of FKN (Cipriani et al., 2011; Morganti et al., 2012; O'Sullivan and Dev, 2017; Pabon et al., 2011); and altered phagocytosis by microglia/macrophages in Cx3cr1 KO mice (Lampron et al., 2015).

We then asked if the effect of FKN on oligodendrocyte density increase is preserved (1) in a rostro-caudal gradient, (2) at a later time point, and whether (3) it is due to OPC differentiation into *de novo* oligodendrocytes. First, we showed injection of FKN directly conjugated to fluorophore Alexa 647 (FKN-647) into the lateral ventricle led to the diffusion into the rostral, medial, and caudal regions of the brain (Figures 3A and 3B). We then subjected OPC lineage tracing mice (PDGFRαCre^{ERT2};RosaYFP$^{+/STOP}$) to a 6-week cuprizone demyelination. Tamoxifen administration in these mice leads to efficient OPC labeling, where 100% of PDGFRα+OLIG2+ cells are YFP+, and 92.9% ± 2.3% of YFP+ cells are PDGFRα+OLIG2+ (Figures S3A–S3C). In the last week of cuprizone treatment, mice were injected with tamoxifen for 5 days to induce recombination in the activated PDGFRα+ OPCs. Seventy-two hours later, mice were subjected to VC/FKN ICV infusion, followed by a 7-day recovery phase (Figure 3C) to allow cortical GM repopulation by *de novo* oligodendrocytes, which occurs at a slower pace compared with CC (Baxi et al., 2017). Exogenous FKN caused an ~1.45- to 1.6-fold increase in the medial cortical GM and CC in the proportion of newborn CC1+OLIG2+YFP+ mature oligodendrocytes (Figures 3D, 3G, 3K, and 3L), but did not alter the total number of YFP+ cells (Figures S3E and S3G). Concomitantly, FKN infusion led to a decrease in the proportion of CC1-OLIG2+YFP+ cells, which represent a mixture of OPCs and immature oligodendrocytes, in the medial cortical GM and CC (Figures 3H and 3M). Similarly, FKN also caused an increase in CC1+OLIG2+YFP+ oligodendrocytes and a decrease in CC1-OLIG2+YFP+ cells in the caudal CC (Figures 3I and 3J). However, there were no changes in the YFP+

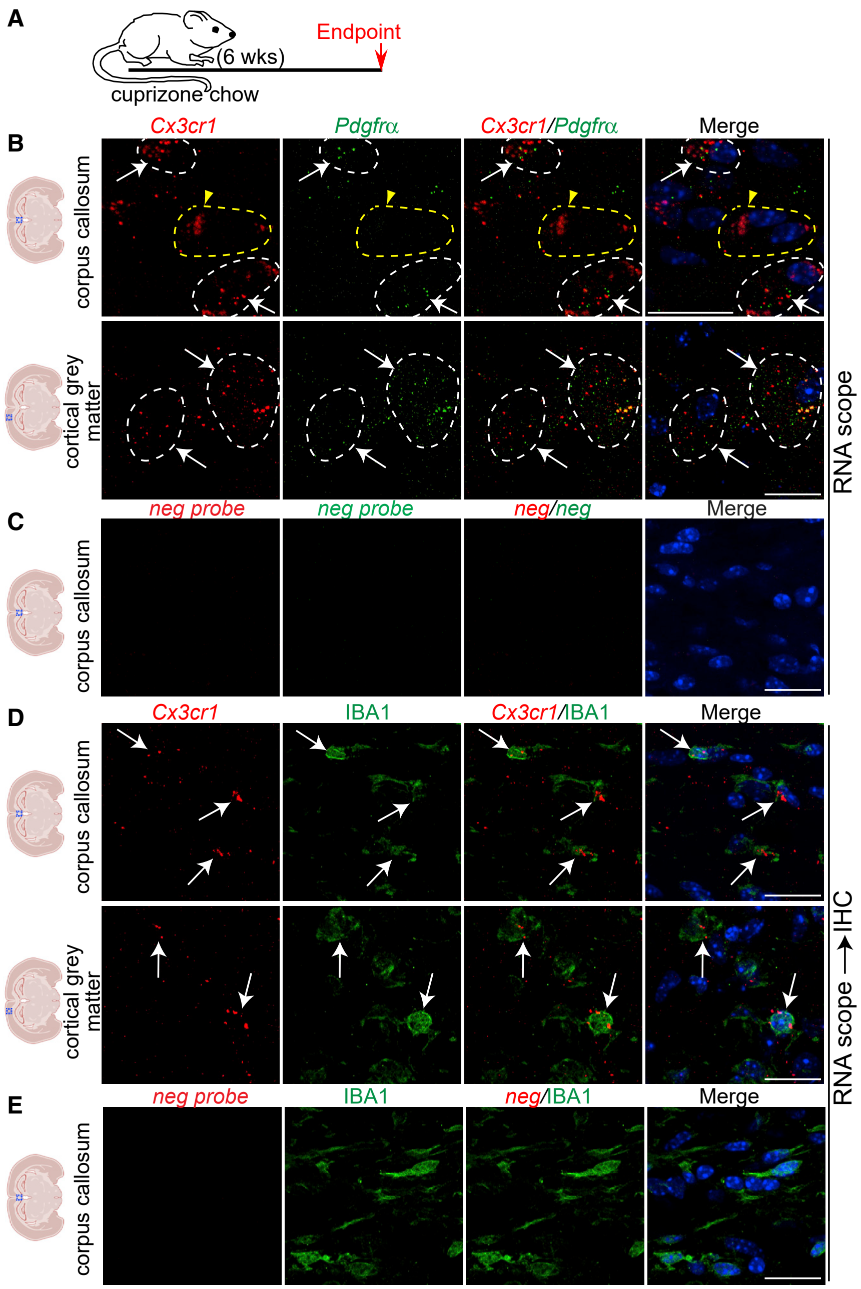

Figure 1. OPCs and microglia express *Cx3cr1* mRNA in demyelinated brain

(A) Ten-week-old C57BL/6J mice were demyelinated with cuprizone chow for 6 weeks.

(B and D) RNA scope analysis of demyelinated brains for *Cx3cr1* (red, B, D) and *Pdgfrα* (green, B) mRNAs or immunostained for IBA1 (green, D). White arrows and dashed lines indicate *Cxc3r1*+marker+ cells. Yellow arrowhead and dashed line indicate a *Cx3cr1+marker-* cell.

(C and E) RNA Scope analysis of midline CC from demyelinated brains with negative probe. Nuclei are visualized with DAPI (blue). Scale bars, 20 μm. n = 2 for each group.

See also Figure S1.

oligodendroglial cells in the rostral CC (Figures 3E and 3F). This is likely because the rostral brain is preferentially remyelinated by SVZ NPCs, rather than parenchymal PDGFRα+ OPCs (Jablonska et al., 2010; Kang et al., 2010; Nait-Oumesmar et al., 1999; Xing et al., 2014).

Thus, FKN infusion leads to a persistent increase in the number of *de novo* oligodendrocytes from activated parenchymal OPCs. As this effect was most prominent in the medial brain regions, we focused our remaining analyses on this area.

Exogenous FKN increases OPC proliferation in remyelinating cortical GM, but not CC

To determine whether an increase in oligodendrocyte formation was due to changes in OPC proliferation, we subjected cuprizone-demyelinated WT mice to VC/FKN ICV and returned to normal chow for 3 days. 24 h before euthanasia, BrdU (Bromodeoxyuridine) was injected to label proliferating cells (Figure 4A). FKN infusion did not lead to changes in the number of PDGFRα+ OPCs or BrdU+ proliferating cells in the midline CC or cortical GM

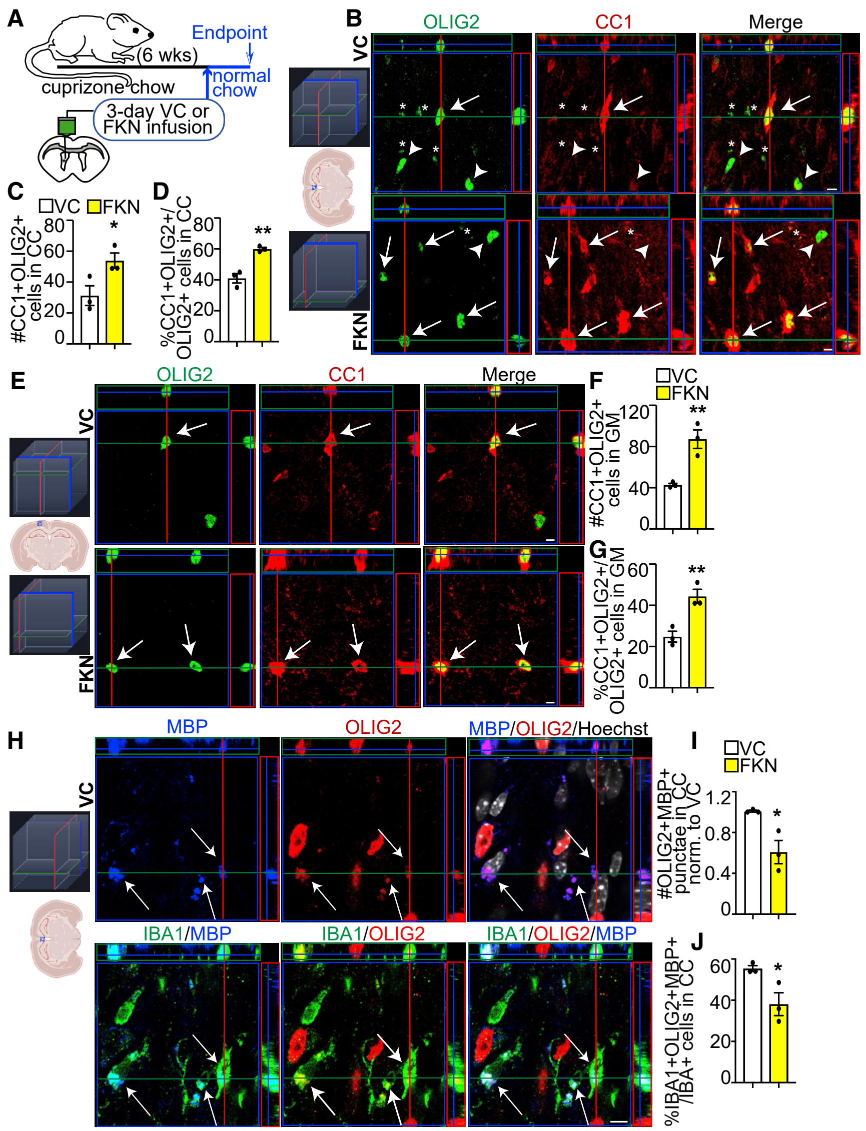

Figure 2. FKN increases oligodendrocyte density after demyelinating injury

(A) Ten-week-old C57BL/6J mice were fed cuprizone chow for 6 weeks. VC/FKN was infused into the lateral ventricle for 3 days via osmotic mini-pump after cuprizone cessation and mice were returned to normal chow.

(B and E) Representative orthogonal slices of z-stack images from midline CC (B) or cortical GM (E) immunostained for CC1 (red) and OLIG2 (green) from VC- (top) and FKN- (bottom) infused mice (see 3D schematic for orientation). Arrows indicate CC1+OLIG2+, arrowheads CC1-OLIG2+ cells, and asterisks OLIG2+ punctate staining (please see Figure 2H for more details).

(C and D) Quantification of (B).

(F and G) Quantification of (E).

(H) Representative orthogonal slices of z-stack images from midline CC immunostained for MBP (blue), OLIG2 (red), and IBA1 (green) from VC-infused mice. Hoechst (white) indicates nuclei (see 3D schematic for orientation). Arrows indicate MBP+ OLIG2+Hoechst- cellular material engulfed by IBA1+ cells.

(I and J) Quantification of (H). Scale bars, 5 μm. Error bars represent SEM. Data were analyzed using unpaired t test, $^{*}p < 0.05$, $^{**}p < 0.01$, n = 3 mice per group.

See also Figure S2.

(Figures 4B–4D and 4G–4J). However, exogenous FKN led to an ~1.6-fold increase in the number of PDGFRα+BrdU+ proliferating OPCs and a trending increase in the proliferative index of OPCs in the cortical GM, but not the CC (Figures 4E, 4F, 4K, and 4L).

Exogenous FKN attenuates microglia/macrophage, but not astrocyte activation

Next, we tested whether exogenous FKN alters microglia biology *in vivo*. Cuprizone-treated WT mice were infused with VC/FKN for 3 days and 24 h before euthanasia, BrdU was injected to label proliferating cells (Figure 5A). Exogenous FKN did not alter the number of total IBA1+ cells, proliferating IBA1+BrdU+ cells, or the proliferative index of IBA1+ cells either in CC, or in cortical GM (Figures 5B–5G).

Previous reports demonstrated FKN infusion or FKN expression in CNS leads to changes in microglia/macrophage activation (Lyons et al., 2009; Pabon et al., 2011; Wang et al., 2021). Moreover, temporal regulation of microglia and macrophage activation is critical for efficient de- and remyelination (Lampron et al., 2015; Miron et al., 2013). In a cuprizone mouse model, the levels of iNOS, TNF-α, and TSPO, which are expressed in pro-inflammatory microglia/macrophages, increase by weeks 5–6 of cuprizone treatment, whereas the levels of CD206, CD163, and Arg1, which are expressed in alternate microglia/macrophages, do not significantly change during or after cuprizone treatment (Abdi et al.,

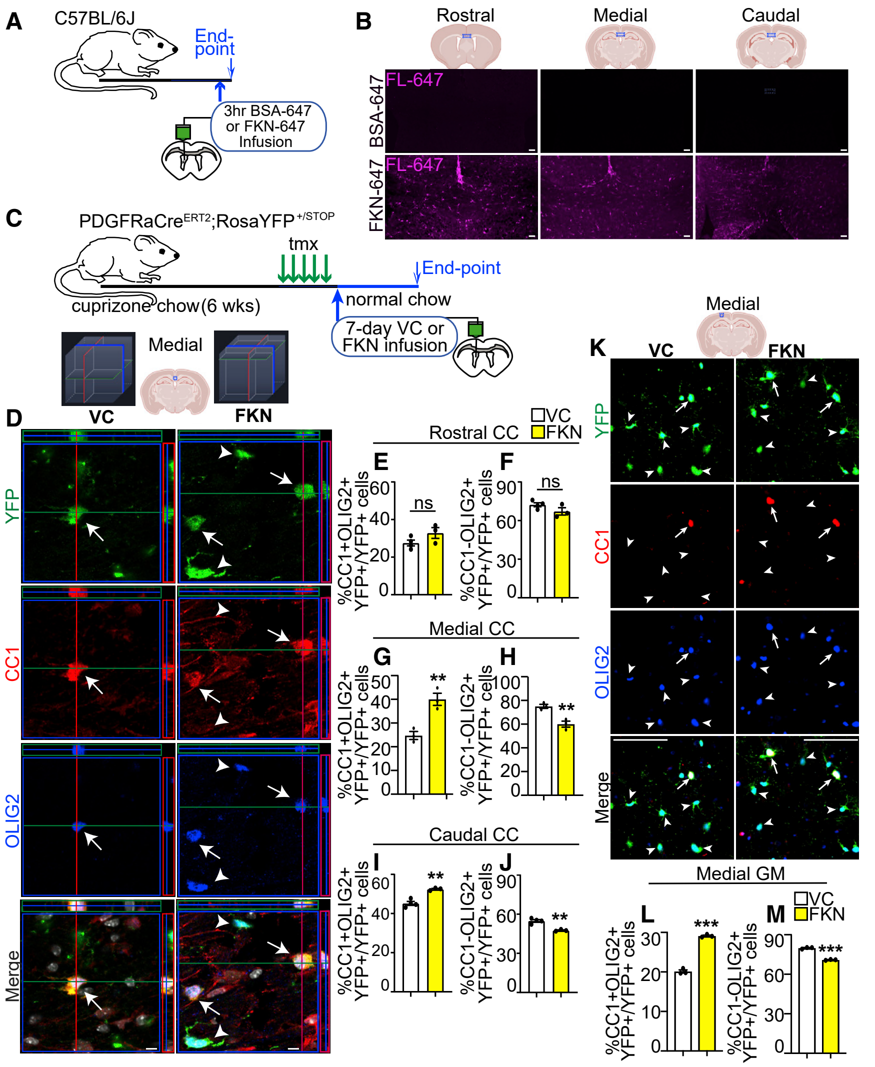

Figure 3. FKN increases *de novo* oligodendrocyte genesis after demyelinating injury

(A) Adult WT mice were infused with FKN-647 or BSA-647 into the lateral ventricle and euthanized 3 h later.

(B) Representative images from BSA-647 (top) and FKN-647 (bottom)-infused brains imaged using fluorescence from far-red (FL-647) channel.

(C) Ten-week-old PDGFRαCre^{ERT2};RosaYFP$^{STOP/+}$ were fed cuprizone chow for 6 weeks. Tamoxifen was injected in the last week of cuprizone treatment. VC/FKN was infused into the lateral ventricle after cuprizone cessation for 7 days and mice were returned to normal chow.

(D) Representative orthogonal slices of z-stack images of midline CC immunostained for YFP (green), CC1 (red), and OLIG2 (blue) from VC- (left) and FKN- (right) infused mice (see 3D schematic for orientation). Arrows indicate YFP+CC1+OLIG2+ and arrowheads YFP+CC1-OLIG2+ cells.

(E–J) Quantification of (D) in rostral (E and F), medial (G and H) and caudal (I and J) CC.

(K) Representative images of medial cortical GM immunostained for YFP (green), CC1 (red), and OLIG2 (blue). Arrows indicate YFP+CC1+OLIG2+ and arrowheads YFP+CC1-OLIG2+ cells.

(L and M) Quantification of (K). Scale bars, 50 μm in (B), 5 μm in (D), 25 μm in (K). Error bars represent SEM. Data were analyzed using unpaired t test, $^{*}p < 0.05$, $^{**}p < 0.01$, n = 3 mice per group from at least two independent litters. See also Figure S3.

2021; Klein et al., 2018). We therefore focused our analysis on CD16/32, which is expressed in pro-inflammatory microglia and macrophages (de Almeida et al., 2020; Miron et al., 2013).

At 3 days of FKN infusion, the proportion and number of CD16/32+IBA1+ microglia/macrophages in the CC and cortical GM were decreased (Figures 5H–5M). Notably, there was a small decrease in the number of CD16/32+IBA1+ microglia/macrophages in the cortical GM, but not CC at 7 days of FKN infusion (Figures S4A–S4E). Regarding astrocyte reactivity, there were no changes in the GFAP astrocyte signal intensity or GFAP+ cell number between VC- and FKN-infused brains (Figures S4F–S4N). Thus, FKN infusion leads to a reduction in pro-inflammatory microglia/macrophage, but not astrocyte, activation.

Exogenous FKN enhances remyelination

Since we observed an increase in oligodendrocyte densities and a reduction in microglia activation as early as 3 days of FKN infusion (Figures 2C and 5I), we performed transmission electron microscopy (TEM) on cuprizone-treated WT animals after 3 days of VC/FKN infusion to assess CC remyelination, similar to (Fernández-Castañeda et al., 2020) (Figures 6A and 6B). FKN infusion resulted in a slightly lower g-ratio (increased myelin thickness) for small-diameter axons with a reversed trend for large-diameter axons (Figure 6C). There was a non-significant trend toward lower average g-ratio for all diameter axons in the FKN-infused mice (Figure 6D). Frequency distribution of g-ratios, however, revealed that FKN caused a shift in g-ratio binning (Figure S5C). When g-ratios were binned into three axon diameter groups, there was a small FKN-mediated reduction in the g-ratio and

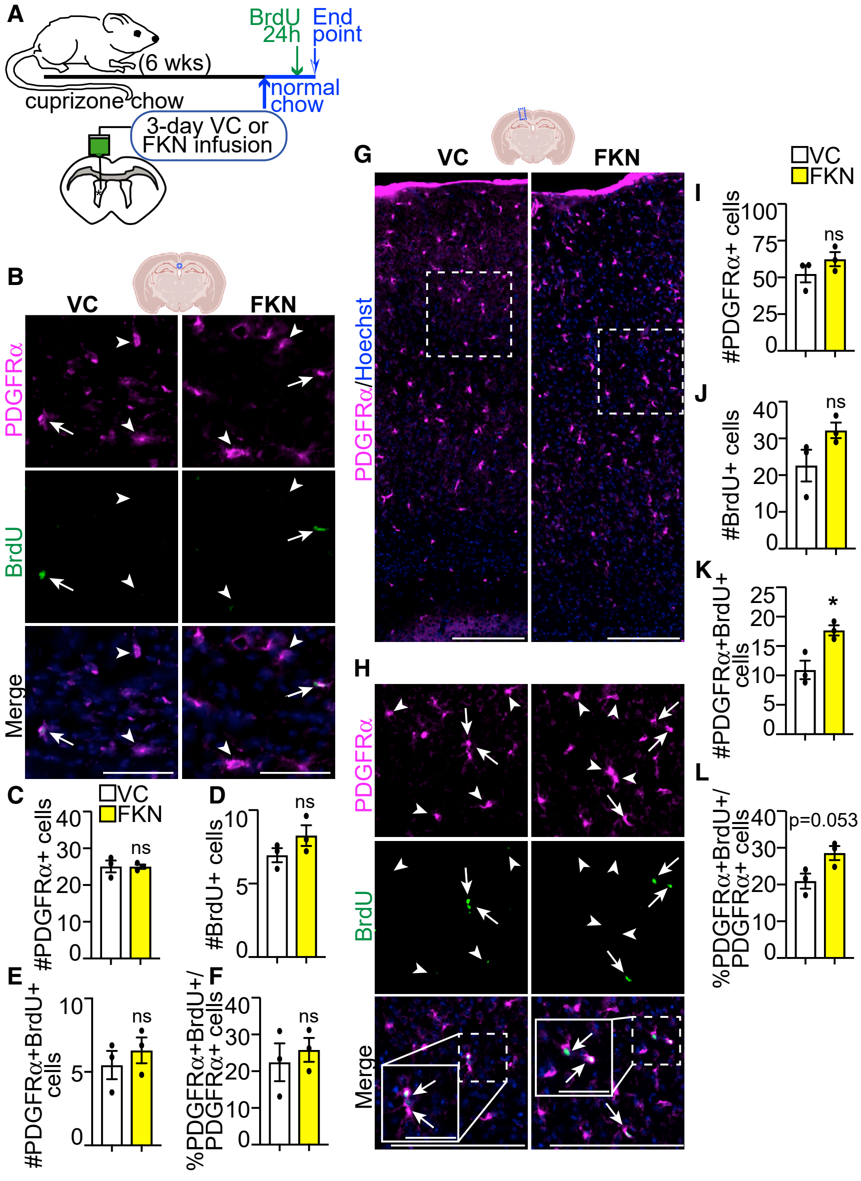

Figure 4. FKN increases OPC proliferation specifically in the remyelinating cortical GM

(A) Ten-week-old C57BL/6J mice were fed cuprizone chow for 6 weeks. VC/FKN was infused into the lateral ventricle for 3 days after cuprizone cessation, and mice were returned to normal chow. BrdU was injected 24 h before euthanasia.

(B) Representative images of midline CC immunostained for PDGFRα (magenta), BrdU (green), and counterstained with Hoechst (blue) from VC- and FKN-infused mice.

(C–F) Quantification of (B).

(G) Representative images of cortical GM immunostained for PDGFRα (magenta) and counterstained with Hoechst (blue) from VC- and FKN-infused mice.

(H) High-magnification images from (G) immunostained for PDGFRα (magenta), BrdU (green), and counterstained with Hoechst (blue). Hatched insets are shown at higher magnification in solid boxes in merge panels. Arrows indicate PDGFRα+BrdU+ and arrowheads PDGFRα+BrdU− cells.

(I–L) Quantification of (G and H). Scale bars, 50 μm in (B), 200 μm in (G and H), 50 μm in (H) insets. Error bars represent SEM. Data were analyzed using unpaired t test, $*p < 0.05$, ns = not significant. n = 3 mice per group.

g-ratio frequency for axons up to 0.5 μm diameter, and no significant changes for axons within 0.51–1.5 μm diameter (Figures 6E and 6F). Notably, there were no changes in axonal density or the relative abundance of axons within any of these bins (Figures S5D and S5E). Furthermore, FKN caused a significant increase in the density and a trending increase in the proportion of total myelinated axons (Figures 6G and 6H). When binned into three axon diameter groups, there was a significant increase in the density and proportion of both small (0.21–0.5 μm) and medium (0.51–1 μm) diameter axons with no changes for the large (1.01–1.5 μm) diameter axons (Figures 6I and 6J).

Next, we assessed differences in myelin or axonal health, critical parameters in myelin biogenesis, and neurodegeneration (Edgar et al., 2020; Peters, 2009). The relative volume of abnormal myelin, which included decompacted and redundant myelin, but not normal compact myelin, was decreased in FKN-infused animals (Figures 6K and S5F). The proportion of degenerating axons was not changed (Figure S5G).

Finally, we asked whether FKN increases cortical GM remyelination. We subjected 10-week-old PDGFRαCre^{ERT2}; Rosa$^{mT/mG}$ mice to a 6-week cuprizone chow, where tamoxifen was injected in the last week of cuprizone treatment. In these mice, all cells express TdTomato (mT). Upon tamoxifen injections, recombined cells express membranous GFP

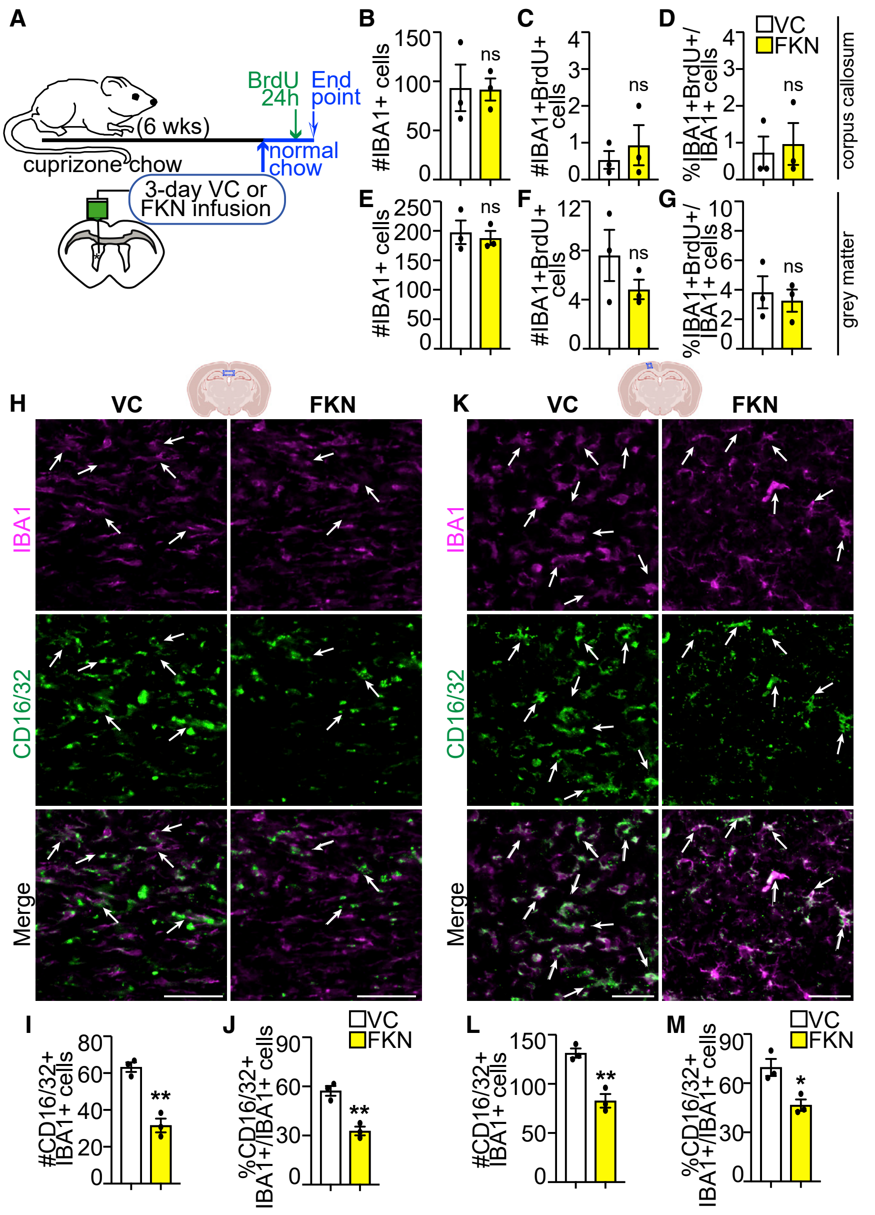

Figure 5. FKN attenuates microglia/macrophage activation in remyelinating brain

(A) Ten-week-old C57BL/6J mice were fed cuprizone chow for 6 weeks. VC/FKN was infused into the lateral ventricle for 3 days after cuprizone cessation, and mice were returned to normal chow. BrdU was injected 24 h before euthanasia.

(B–G) Analysis of midline CC (B–D) and cortical GM (E–G) for average number of total IBA1+ cells (B and E), IBA1+BrdU+ (C and F), and proliferative index of IBA1+ cells (%IBA1+BrdU+/IBA1+ cells [D and G]).

(H and K) Representative images of midline CC (H) or cortical GM (K) immunostained for IBA1 (magenta) and CD16/32 (green) from VC- and FKN-infused mice. Arrows indicate IBA1+CD16/32+ microglia/macrophages.

(I, J, L, and M) Quantification of (H and K). Scale bars, 50 μm. Error bars represent SEM. Data were analyzed using unpaired t test, $^{**}p < 0.01$, $^{*}p < 0.05$, ns = not significant. n = 3 mice per group.

See also Figure S4.

(mG) (Muzumdar et al., 2007). Since myelin is enriched in the oligodendrocyte cell membranes, a co-localization analysis between MBP, which is highly enriched in compact myelin (Snaidero et al., 2017), and mG is indicative of the formation of new myelin arising from differentiating PDGFRα+ OPCs. VC or FKN were infused for 7 or 21 days, during which time mice were returned to normal chow (Figure 6L). FKN increased new myelin formation by ~1.8- to 2-fold when compared with VC after 7–21 days of infusion (Figures 6M–6P). Notably, these results are comparable to clemastine, the only remyelinating agent currently in clinical trials (Li et al., 2015).

Taken together, these results show FKN increases remyelination in both CC and cortical GM.

FKN modulates proliferation and differentiation of OPCs cultured with and without microglia

To ask whether FKN modulates OPC biology directly or indirectly via actions on microglia, we cultured isolated primary cortical OPCs and microglia (Figures 7A, 7H, S6A–S6D, S6E–S6G), alone or together.

Exogenous FKN increased the proportion of MBP+ OLIG2+ oligodendrocytes from OPCs cultured in the absence or presence of microglia (Figures 7B–7D) in

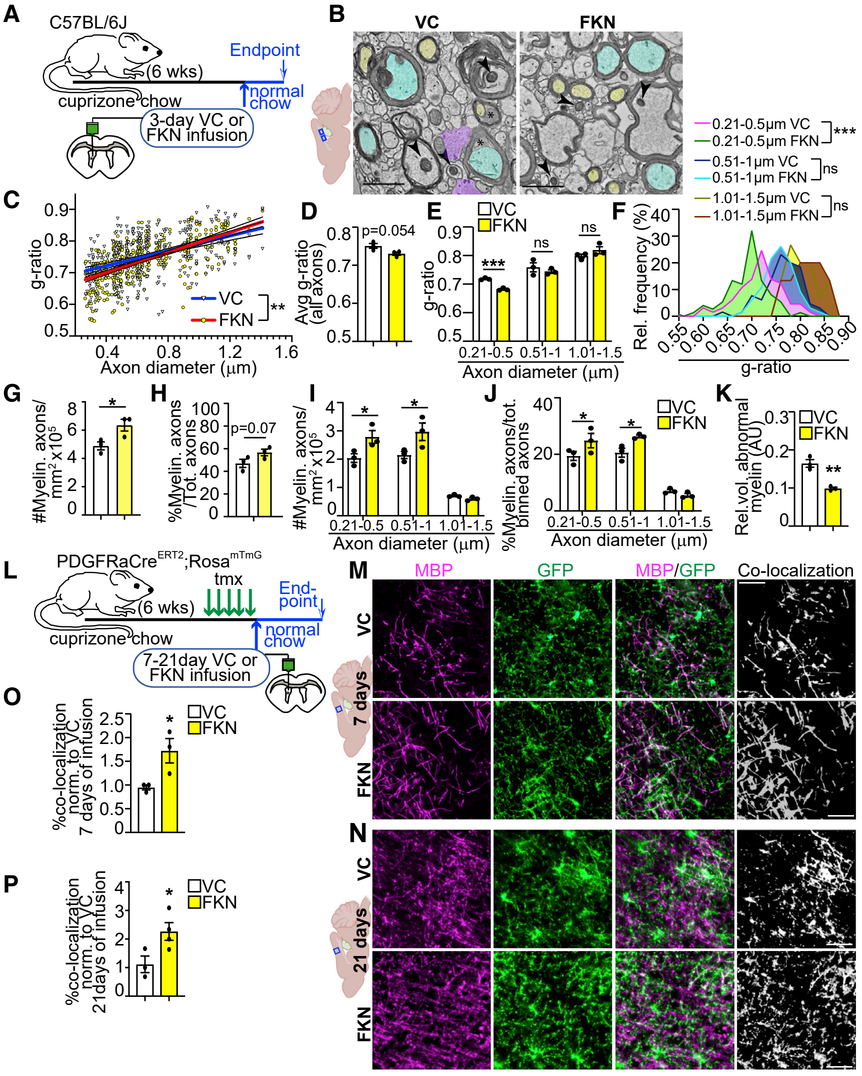

Figure 6. FKN enhances remyelination

(A) Ten-week-old C57BL/6J mice were fed cuprizone chow for 6 weeks. VC/FKN was infused into the lateral ventricle for 3 days after cuprizone cessation, and mice were returned to normal chow.

(B) Representative TEM images of CC sections from VC- and FKN-infused mice. Arrowheads designate well-preserved mitochondria, asterisks abnormal myelin, yellow and blue small and medium diameter axons, respectively, and purple glial fibrillar protein positive regions.

(C–F) Analysis of (B) for g-ratio versus axon diameter correlation (C), average g-ratio for all axons (D), and average g-ratio (E) or g-ratio frequency distribution (F) for binned axons.

(G–J) Analysis of (B) for total (G and H) or binned (I and J) myelinated axon density (G and I) or proportion (H and J).

(K) Morphometric analysis of abnormal myelin. AU = arbitrary units.

(L) Ten-week-old PDGFRαCre^{ERT2};Rosa$^{mT/mG}$ were fed cuprizone chow for 6 weeks. Tamoxifen was injected in the last week of cuprizone treatment. VC/FKN was infused into the lateral ventricle after cuprizone cessation for 7–21 days and mice were returned to normal chow.

(M and N) Representative images of VC- (top) and FKN- (bottom) infused cortical GM immunostained for MBP (magenta) and GFP (green) after 7 (M) and 21 (N) days of infusion. White represents MBP and GFP co-localization.

(O and P) Quantification of (M), (N) normalized to VC after 7 (O) and 21 (P) days of infusion.

Scale bars, 1 µm in (B), 50 µm in (M and N). Error bars represent SEM. Each data point in (C) represents an axon, and in (D–K) a mouse. Data were analyzed using unpaired t test, except data in (C and F) were analyzed with linear regression and data in (E, I, and J) with multiple t test. ***$p < 0.001$, **$p < 0.01$, *$p < 0.05$, ns = not significant, n = 3–4 mice per group.

See also Figure S5.

OPC differentiation media containing T3 (triiodothyronine), a potent inducer of oligodendrocyte differentiation (herein referred to as OPC-DM) (Watson et al., 2021). It is known that PDGF-AA is highly expressed in CC but not cortical GM, and that OPCs in the white matter, but not GM, proliferate in response to PDGF-AA (Hill et al., 2013; Krupinski et al., 1997). We thus asked whether FKN modulates OPC proliferation in OPC-DM, which is devoid of growth factors. Figures S6D, 7E, and 7F show FKN increased OPC proliferative index in the presence and absence of microglia. Therefore, FKN enhances OPC proliferation and differentiation at least in part by directly affecting OPCs.

Next, we investigated changes in the interplay between OPCs and microglia after exposure to FKN. We pre-treated OPCs and/or microglia with VC or FKN, followed by VC/FKN washout and subsequent co-cultures of pre-treated cells in OPC-DM (without additional FKN) (Figure 7A). Figure 7G shows that the proportion of MBP+OLIG2+ oligodendrocytes was only increased in co-cultures, in which both OPCs and microglia were pre-treated with FKN. Pre-treating only OPCs or microglia with FKN did not alter the proportion of oligodendrocytes (Figure 7G).

These results provide a novel mechanistic insight into FKN-CX3CR1 signaling and demonstrate that FKN acts

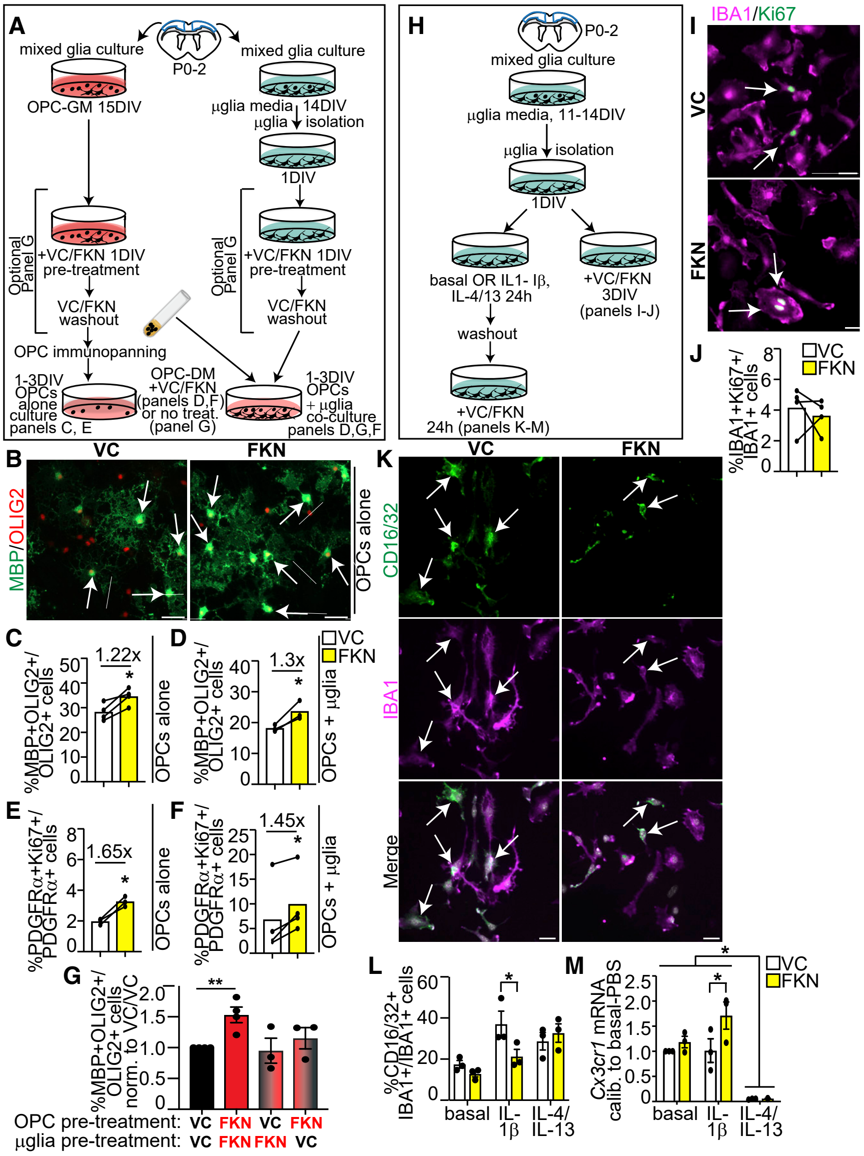

Figure 7. FKN regulates OPC and microglia cell fates *in vitro*

(A) OPCs were cultured with or without microglia in OPC DM and VC/FKN for 1–3 days *in vitro* (DIV).

(B) Representative images of OPCs cultured in VC or FKN for 3DIV and immunostained for MBP (green) and OLIG2 (red). Arrows designate MBP+OLIG2+ oligodendrocytes.

(C–F) Quantification of % MBP+Olig2+ cells at 3DIV (C and D) and OPC proliferative index at 1DIV (E and F) in OPC cultures (C, E) or OPC-microglia co-cultures (D and F).

(G) Quantification of % MBP+OLIG2+ oligodendrocytes in co-cultures with VC- and/or FKN-pre-treated OPCs and microglia.

(H) Microglia were incubated with VC/FKN with or without pre-stimulation with IL-1β and IL-4/IL-13 cytokines or no cytokines (basal conditions).

(I) Representative images of microglia treated with VC/FKN for 3DIV and immunostained with IBA1 (purple) and Ki67 (green).

(J) Quantification of (I).

(K) Representative images of IL-1β-pre-stimulated microglia treated with VC/FKN and immunostained with CD16/32 (green), IBA1 (purple) and counterstained with Hoechst (white).

(L) Quantification of (K).

(M) qPCR analysis of *Cx3cr1* mRNA expression normalized to *Gapdh* and *Hnrnpab* housekeeping genes and calibrated against basal VC-treated microglia. Scale bars, 20 μm. Error bars represent SEM. Each connected line corresponds to one biological replicate. Data were analyzed with Student's paired t test, except graph in (G) was analyzed with one-way ANOVA followed by Dunnett's post hoc test, graph in (L) was with multiple t test and graph in (M) with two-way ANOVA followed by Tukey post hoc test. $^{**}p < 0.01$, $^{*}p < 0.05$, n = 3–4 independent experiments.

See also Figure S6.

on both OPCs and microglia to increase oligodendrocyte formation.

FKN modulates microglia biology *in vitro*

Next, we assessed *in vitro* microglia response to FKN. Exogenous FKN did not alter the proportion or proliferative index of IBA1+ microglia (Figures S6F, 7I, and 7J). We then incubated microglia with or without polarizing cytokines IL-1β or IL-4/IL-13 for 1DIV (Figure 7H). We confirmed TNF-α, a pro-inflammatory cytokine, was upregulated specifically in media conditioned by IL-1β pre-treated microglia (Figure S6H). Incubation of basal or pre-stimulated microglia with FKN did not affect the proportion of total IBA1+ microglia in basal, interleukin (IL)-1β, or IL-4/IL-13 conditions (Figures 7H and S6G). However, FKN reduced the proportion of CD16/32+IBA1+ IL-1β pre-stimulated microglia by ~1.43-fold (Figures 7K and 7L). Thus, in agreement with *in vivo* results, FKN reduces CD16/32+ microglia *in vitro*.

It was recently shown that Cx3cr1 overexpression reduces pro-inflammatory microglia/macrophages or mediators (Inoue et al., 2021; Zhang et al., 2019). We thus hypothesized that FKN could be attenuating microglia activation by modulating Cx3cr1 expression. qRT-PCR

revealed *Cx3cr1* mRNA was expressed in basal and IL-1β pre-stimulated microglia, but its expression was negligible in IL-4/IL-13 pre-stimulated microglia (Figure 7M). Notably, *Cx3cr1* mRNA was ~1.7-fold upregulated by FKN treatment specifically in the IL-1β pre-stimulated microglia (Figure 7M). Therefore, FKN reduces pro-inflammatory microglia activation and upregulates *Cx3cr1* receptor expression.

Finally, we determined the phagocytic ability of microglia in the presence of FKN. Here, we treated isolated primary murine microglia with VC/FKN for 3 h in the presence of myelin debris conjugated to pHRodo, which fluoresces in the acidic lysosomal environment (pH ~5.5) (Bhattacherjee et al., 2019) (Figure S6I). Figures S6J and S6K shows FKN-treated microglia had an ~1.2-fold increase in pHRodo mean fluorescence intensity when compared with VC. Thus, FKN increases myelin debris phagocytosis by microglia *in vitro*.

DISCUSSION

Our data demonstrate exogenous FKN engages parenchymal OPCs, which express *Cx3cr1*, for oligodendrocyte regeneration and remyelination in a cuprizone demyelination mouse model.

Prior reports demonstrate administration of exogenous soluble FKN before induction of stroke or Parkinson-like lesions leads to neuroprotection (Cipriani et al., 2011; Morganti et al., 2012). However, the ability of FKN to promote regeneration after a demyelinating injury has not been tested. We show that ICV administration of soluble FKN after cuprizone-induced demyelination leads to increased production of *de novo* oligodendrocytes and remyelination *in vivo* from activated parenchymal OPCs.

Our results also show exogenous FKN increases small-diameter axon myelination. Intriguingly, patients with MS show specific neurodegeneration of small caliber axons (Evangelou et al., 2001; Lassmann, 2003). Our work highlights the potential neuroprotective role of FKN for these small caliber axons and raises an intriguing question about oligodendrocyte-axon interaction. Pío del Río-Hortega described four types of oligodendrocytes, from which type I and II oligodendrocytes myelinate small-diameter axons (reviewed in Simons and Nave [2015]). While we have previously shown FKN increases oligodendrocyte-axon interactions (Watson et al., 2021), we do not currently understand whether FKN stimulates the generation of specific oligodendrocyte subtype(s) that may have a preference for small-diameter axon myelination, and the biological significance of this phenomenon.

Notably, it was previously demonstrated that a CX3CR1 allosteric modulator AZD8797, which is proposed to act as an antagonist and which does not cross the blood brain barrier, protects from autoimmune demyelination by blocking peripheral leukocyte infiltration (Ridderstad Wollberg et al., 2014). On the other hand, FKN infusion or overexpression specifically in the CNS has been shown to have beneficial effects in normal healthy mice as well as rodent models of stress, stroke, schizophrenia, synucleinopathy, as well as Parkinson and Alzheimer diseases (Watson et al., 2021, and reviewed in Watson et al., 2020). Our results extend these reports and demonstrate that infusion of FKN into demyelinated CNS increases oligodendrocyte and myelin regeneration.

Our experimental design does not allow distinguishing direct *in vivo* effects of FKN on OPCs versus microglia. Our *in vitro* experiments, however, show FKN directly modulates OPC fates when cultured without microglia. Surprisingly, our co-culture results demonstrate that both OPCs and microglia need to be pre-treated with FKN to elicit an increase in oligodendrocyte formation. These results suggest that FKN acts on both OPCs and microglia to increase oligodendrocyte formation.

We also show CC and cortical GM OPCs display a differential proliferation response to FKN infusion, which was not associated with a differential response in microglia or astrocyte activation. It was previously shown that white matter and GM OPCs are transcriptionally homogeneous yet respond differently to IFNγ (Sherafat et al., 2021a). In the future, it will be interesting to determine whether FKN elicits different signaling cascades in white matter versus GM OPCs, and/or whether this is mediated by other cell types.

In summary, we demonstrate that infusion of FKN into the brain after demyelinating injury increases oligodendrocyte and myelin regeneration from parenchymal OPCs. Therefore, FKN represents a novel candidate for remyelination strategies.

EXPERIMENTAL PROCEDURES

Resource availability

Corresponding author

Further information and requests for resources and reagents should be directed to and fulfilled by the lead contact, Anastassia Voronova (voronova@ualberta.ca).

Materials availability

This study did not generate new unique reagents.

Data and code availability

This study did not generate new unique datasets.

Growth factors and cytokines

For cell cultures, murine soluble FKN (R&D Systems) was reconstituted in 1X PBS and used at 250 ng/mL. For ICV infusions, FKN was reconstituted in 0.2% BSA in 1X PBS and infused at 200 ng/day rate

using Alzet osmotic mini-pumps as per Watson et al. (2021). Please see supplemental materials and methods for other details.

Experimental model and subject details

Mice

Animal use protocols were approved by the Research Ethics Office at the Universities of Alberta and Ottawa in accordance with the Canadian Council of Animal Care Policies. Mice from both sexes were used for all experiments. PDGFRαCre^{ERT2} (*B6N.Cg-Tg(Pdgfra-cre/ERT)467Dbe/J*; RRID:IMSR_JAX:018280), RosaYFP^{STOP} (*B6.129X1-Gt(ROSA)26Sortm1(EYFP)Cos/J*; RRID:IMSR_JAX:006148), Rosa$^{mT/mG}$ (*B6.129(Cg)-Gt(ROSA)26Sortm4(ACTB-tdTomato,-EGFP)Luo/J*; RRID:IMSR_JAX:007676) and WT C57BL/6J mice were obtained from Jackson Laboratories (Kang et al., 2010; Muzumdar et al., 2007; Srinivas et al., 2001). CD1 mice were purchased from Charles River Laboratory.

Cuprizone-induced demyelination experiments

Ten-week-old mice were subjected to nutragel (Bio-Serv) *ad libitum* containing 0.2% cuprizone (Sigma) for 6 weeks.

Tamoxifen injections

PDGFRαCre^{ERT2};RosaYFP^{STOP} or PDGFRαCre^{ERT2};Rosa$^{mT/mG}$ animals were injected with 3 mg tamoxifen for 5 days as described in Li et al. (2022) and Watson et al. (2021). Seventy-two hours after last tamoxifen injection, ICV surgery was performed as per below.

ICV infusions

Infusions are described in Li et al. (2022) and Watson et al. (2021). Briefly, infusion was performed into the right ventricle using the following coordinates relative to bregma: −1.000 ML, −0.300 AP, −2.500 DV. For one-time injection, 0.5–1 μL of FKN-647 or matched volume and equimolar amount of BSA-647 were injected once into 2- to 3-month-old WT C57BL/6J mice as described in Watson et al. (2021). For multi-day infusion, 7-day or 28-day osmotic mini-pumps (Alzet, 1007D or 1004) were connected containing VC (vehicle-control) or FKN in VC. After surgery, mice were returned to normal chow. BrdU (Sigma) was injected at 100 mg/kg dose 24 h before perfusions.

Lysolecithin injections

Lysolecithin (LPC)-mediated demyelination was performed as described in Kosaraju et al. (2020). Briefly, 1-month-old WT mice were injected with LPC (Sigma, L1381) (1 μL of 1% solution in 1x PBS) using a stereotactic apparatus at two sites: +1.0 mm AP, +1.0 mm ML, −2.2 mm DV and +0.7 mm AP, +1.0ML, −2.2 DV. Animals were allowed to recover for 14 days.

Myelin isolation

Myelin was isolated as described in Bhattacherjee et al. (2019). Briefly, brains from 5-month-old Sprague-Dawley rats were homogenized, layered over sucrose, ultracentrifuged, and purified via density gradients. Purified myelin was labeled with pHRodo (Invitrogen) and stored at −80°C.

Tissue dissection and collection

Mice under 21 days of age were euthanized with CO_2, and over 21 days with 102 mg/kg of body weight Euthansol (WDDC), followed by transcardiac perfusion with HBSS and 4% paraformaldehyde (PFA, Acros Organics). For TEM, animals were perfused with HBSS followed by 4% PFA in 0.1M Sodium Cacodylate buffer with 2 mM $CaCl_2$. Dissected regions of interest were incubated with 2% PFA, 2.5% Glutaraldehyde, 0.1M Sodium Cacodylate buffer, and 2 mM $CaCl_2$, infiltrated with Spurr's resin and polymerized. Sections were cut from two blocks per sample using an ultramicrotome (Leica, EM UC6).

Primary cell cultures

Complete methods are in the supplemental information.

Microglia cultures. Microglia were cultured from P0-P2 CD1 cortices in DMEM/F12 (Gibco) with 10% FBS (Sigma), 1% Pen/Strep, 1% sodium pyruvate, and 50 μM β-mercaptoethanol (herein referred to as **microglia media**) as per Galleguillos et al. (2022) with slight modifications. Namely, 10 ng/mL GM-CSF was added on days 8–11. Microglia were isolated on day 14 using lidocaine (Sigma) and shaking. For proliferation analysis, microglia were cultured with VC/FKN and without β-mercaptoethanol for 3 days. For co-cultures, isolated microglia were optionally pre-treated with VC/FKN for 24 h. Please see supplemental materials and methods for more details. VC/FKN was washed off, and isolated OPCs (with or without VC/FKN pre-treatment) were plated on top of microglia as described below.

Cortical OPC cultures. OPCs were cultured from P0-P2 CD1 cortices in Serum-Free Media (SFM; please see supplemental materials and methods for details) supplemented with 2% B27 supplement (Life Technologies), 10 ng/mL FGF (Peprotech), and 10 ng/mL PDGF-AA (R&D) (herein referred to **OPC growth media [OPC-GM]**). On day 15, cultures were optionally pre-treated with VC or FKN for 24 h. On day 16, VC/FKN was washed off. OPCs were immunopanned on day 15 (without pre-treatment) or day 16 (when cells were pre-treated) with PDGFRα-specific magnetic beads (Miltenyi). Recovered cells were cultured on top of microglia or alone in SFM with 2% B27 and 40 ng/mL 3,3′,5-Triiodo-L-thyronine (T3, Sigma) and VC/FKN (herein referred to **OPC differentiation media [OPC-DM]**) for 1–3 days.

Microglia polarization. Isolated microglia were treated with no cytokines (basal conditions), 10 ng/mL IL-1β (Cedarlane), or 20 ng/mL IL-4 (Miltenyi) and IL-13 (R&D Systems) for 24 h. Media was changed and VC/FKN was added for additional 24 h.

*Myelin phagocytosis assays*pHRodo-Myelin and VC/FKN was added to microglia cultured in basal conditions for 3 h and imaged as described below.

Conditioned medium preparation/ELISA. Conditioned medium was collected from pre-stimulated microglia, centrifuged at 2,000 × *g* for 7 min to remove dead cells, and subjected to TNF-α ELISA kit (Invitrogen).

Reagents and immunostaining

Immunocytochemistry

Cell cultures were fixed and stained as described in Watson et al. (2021) with antibodies listed in the supplemental materials and methods. Nuclei were counterstained with Hoechst 33,258 (Riodel-De Haen Ag).

Immunohistochemistry

Cryopreserved brains were sectioned at 18 μm. For FKN-647 diffusion assay, sections were rehydrated in 1X PBS, immediately counterstained with Hoechst 33258 to visualize nuclei. Staining with FluoroMyelin was performed as per manufacturer's instructions (Invitrogen). For all other staining, sections were stained as described in Li et al. (2022) and Watson et al. (2021). Antibodies

are listed in the supplemental materials and methods. Nuclei were counterstained with Hoechst 33258.

RNA scope

Brain cryosections from WT demyelinated mice were subjected to RNA scope as described in Voronova et al. (2017) and Watson et al. (2021) with probes targeting murine *Cx3cr1* and/or *Pdgfrα* mRNA or negative control probe according to the manufacturer's instructions (ACD). Some sections were counterstained with anti-IBA1 antibodies as in Voronova et al. (2017).

Microscopy

Brain sections were imaged with (1) Zeiss LSM700 confocal microscope with photomultiplier tube (PMT) with ×40 objective, where high-magnification digital image acquisition was performed with 2–5x digital Zoom using Zen (Zeiss), (2) Olympus IX81 fluorescence microscope equipped with Okogawa CSU X1 spinning disk confocal scan head, ×20 objective and a Hamamatsu camera (Hamamatsu), where digital image acquisition was performed with Volocity (Perkin Elmer) software, or (3) with Zeiss Axio Imager M2 fluorescence microscope, ORCA-Flash LT sCMOS Camera and the Zen software (Zeiss). Z-stacks spanning 6–10 μm were taken with optical slice thickness 0.2–0.5 μm and stacked images or orthogonal sections through 3D projections are shown.

Images from all fixed primary cell culture experiments were captured with ×20 objective using Zeiss Axio Imager M2 fluorescence microscope, ORCA-Flash LT sCMOS Camera, and the Zen software (Zeiss). Images were captured in a single plane.

Live microglia cultures were imaged using an inverted Zeiss Axio Observer Z1 microscope equipped with Axiocam 503 Mono camera and ×20 objective.

For TEM, images were acquired using JEOL JEM-2100, Gatan Orius camera with digital micrograph at 200 kV acceleration voltage.

RNA isolation and qRT-PCR

Total RNA was purified using Omega Biotek E.Z.N.A. microelute kit, and reverse transcribed to generate cDNA using QuantiTect Reverse Transcription Kit (Qiagen) as described in Voronova et al. (2017). qPCR was performed using the Ssoadvanced SYBR Green kit (BioRad), primers listed in the supplemental materials and methods, and using Eppendorf Realplex2 (Eppendorf) instrument. Data were normalized to *Gapdh* and *Hnrnpab* and analyzed using $2^{-\Delta\Delta Ct}$ method.

Quantification, co-localization and statistical analysis

In vitro data are presented from at least three independent biological experiments. Five to 10 fields of view and at least 250 cells from each treatment and biological experiment were counted.

In vivo analysis was performed in a blind fashion by two independent observers. Five matched sections per brain were analyzed from six mice across three independent litters. At least 300 cells per CC and 800 cells per cortical column in every sample were counted.

For co-localization analysis, cortical GM was imaged using ×20 objective and captured using Z-stacks with an optical thickness of 0.5 μm spanning 5–8 μm. Z-stacks were subjected to Imaris and voxel-based MBP/mGFP co-localization analysis was performed.

TEM images were analyzed as described in Bando et al. (2015) and Edgar et al. (2020). In brief, the g-ratio was measured for myelinated axons, where axon and compact myelin morphology were normal. At least eight images and 100 axons per sample were analyzed. The number of intersections of normal and abnormal myelin with 64 equidistant grids overlaid in Fiji software was counted. Axonal density was analyzed using axons that fit within the ROI. At least 30 images per sample were analyzed.

All data were subjected to normality tests with D'Agostino & Pearson, Shapiro-Wilk, and Kolmogorov-Smirnov tests and were considered normal. For two group comparisons, two-tailed paired Student's t tests (*in vitro* datasets) or two-tailed unpaired Student's t tests (for *in vivo* datasets) or multiple t tests were used to assess statistical significance between means, where a p value <0.05 was considered significant. For three or more group comparisons, one-way or two-way ANOVA followed by Dunnett's or Tukey multiple comparisons tests were used. For comparing slopes of g-ratios with axon diameters, simple linear regression was applied where 95% CI of the best-fit line was used.

SUPPLEMENTAL INFORMATION

Supplemental information can be found online at https://doi.org/10.1016/j.stemcr.2022.12.001.

AUTHOR CONTRIBUTIONS

Conceptualization, M.M.A.A., A.V.; Methodology, M.M.A.A., A.E.S.W., T.F., A.V., N.S., D.G., J.K., J.W., J.M.E., M.N.-N., B.W.; Formal Analysis, M.M.A.A., A.E.S.W., N.L.D., S.B., P.S., B.S.W., K.G., J.M.E., A.V.; Investigation, M.M.A.A., A.E.S.W., N.L.D., D.G., S.S., A.V.; Resources, A.V., S.S., F.G.; Writing-Original Draft, A.V.; Writing-Revisions, A.E.S.W. Writing-Review & Editing, M.M.A.A., A.E.S.W., N.L.D., J.M.E., A.V. Supervision, S.S., J.M.E., A.V.; Funding Acquisition, A.V.

ACKNOWLEDGMENTS

This work was funded by MS Society of Canada (3573) and CIHR operating grants (PS 166120), CIHR team grant (NEURON 161466 under the frame of Neuron Cofund), and Canada Research Chairs award in Neural Stem Cell Biology awarded to A.V., UK MS Society Grant (#37) to J.M.E., and by NSERC Discovery grant (RGPIN-2016-06343) and GlycoNet (ND-05) to S.S. M.M.A.A. received IBRO Short state grant to train in TEM by J.M.E. A.E.S.W. and S.B. were supported by the MS Society of Canada EndMS scholarships; A.E.S.W., K.G. and B.S.W. by WCHRI Scholarships; N.L.D. by the NSERC PGS-D, Brad Mates E Drive for Research Fund and the NMHI. We thank Drs. Hughes and Simmonds for access to the confocal microscope, Imaris software, and qRT-PCR instrument; Tracy Tan and Fajar Khan for technical assistance. TEM was carried out in the Experimental Oncology Cell Imaging Facility at the University of Alberta.

CONFLICT OF INTERESTS

The authors declare no competing interests.

Received: March 14, 2022
Revised: December 6, 2022
Accepted: December 7, 2022
Published: January 5, 2023

REFERENCES

Abdi, M., Pasbakhsh, P., Shabani, M., Nekoonam, S., Sadeghi, A., Fathi, F., Abouzaripour, M., Mohamed, W., Zibara, K., Kashani, I.R., and Zendedel, A. (2021). Metformin therapy attenuates pro-inflammatory microglia by inhibiting NF-κB in cuprizone demyelinating mouse model of multiple sclerosis. Neurotox Res *39*, 1732–1746.

Bando, Y., Nomura, T., Bochimoto, H., Murakami, K., Tanaka, T., Watanabe, T., and Yoshida, S. (2015). Abnormal morphology of myelin and axon pathology in murine models of multiple sclerosis. Neurochem. Int. *81*, 16–27. https://doi.org/10.1016/j.neuint.2015.01.002.

Baxi, E.G., DeBruin, J., Jin, J., Strasburger, H.J., Smith, M.D., Orthmann-Murphy, J.L., Schott, J.T., Fairchild, A.N., Bergles, D.E., and Calabresi, P.A. (2017). Lineage tracing reveals dynamic changes in oligodendrocyte precursor cells following cuprizone-induced demyelination. Glia *65*, 2087–2098. https://doi.org/10.1002/glia.23229.

Bhattacherjee, A., Rodrigues, E., Jung, J., Luzentales-Simpson, M., Enterina, J.R., Galleguillos, D., St Laurent, C.D., Nakhaei-Nejad, M., Fuchsberger, F.F., Streith, L., et al. (2019). Repression of phagocytosis by human CD33 is not conserved with mouse CD33. Commun. Biol. *2*, 450. https://doi.org/10.1038/s42003-019-0698-6.

Cardona, S.M., Kim, S.V., Church, K.A., Torres, V.O., Cleary, I.A., Mendiola, A.S., Saville, S.P., Watowich, S.S., Parker-Thornburg, J., Soto-Ospina, A., et al. (2018). Role of the fractalkine receptor in CNS autoimmune inflammation: new approach utilizing a mouse model expressing the human CX3CR1(I249/M280) variant. Front. Cell. Neurosci. *12*, 365. https://doi.org/10.3389/fncel.2018.00365.

Cipriani, R., Villa, P., Chece, G., Lauro, C., Paladini, A., Micotti, E., Perego, C., De Simoni, M.G., Fredholm, B.B., Eusebi, F., et al. (2011). CX3CL1 is neuroprotective in permanent focal cerebral ischemia in rodents. J. Neurosci. *31*, 16327–16335. https://doi.org/10.1523/jneurosci.3611-11.2011.

de Almeida, M.M.A., Pieropan, F., de Mattos Oliveira, L., dos Santos Junior, M.C., David, J.M., David, J.P., da Silva, V.D.A., dos Santos Souza, C., Costa, S.L., and Butt, A.M. (2020). The flavonoid agathisflavone modulates the microglial neuroinflammatory response and enhances remyelination. Pharmacol. Res. *159*, 104997. https://doi.org/10.1016/j.phrs.2020.104997.

Edgar, J.M., Smith, R.S., and Duncan, I.D. (2020). Transmission electron microscopy and morphometry of the CNS white matter. Methods Mol. Biol. *2143*, 233–261. https://doi.org/10.1007/978-1-0716-0585-1_18.

Evangelou, N., Konz, D., Esiri, M.M., Smith, S., Palace, J., and Matthews, P.M. (2001). Size-selective neuronal changes in the anterior optic pathways suggest a differential susceptibility to injury in multiple sclerosis. Brain *124*, 1813–1820. https://doi.org/10.1093/brain/124.9.1813.

Fernández-Castañeda, A., Chappell, M.S., Rosen, D.A., Seki, S.M., Beiter, R.M., Johanson, D.M., Liskey, D., Farber, E., Onengut-Gumuscu, S., Overall, C.C., et al. (2020). The active contribution of OPCs to neuroinflammation is mediated by LRP1. Acta Neuropathol. *139*, 365–382. https://doi.org/10.1007/s00401-019-02073-1.

Galleguillos, D., Wang, Q., Steinberg, N., Zaidi, A., Shrivastava, G., Dhami, K., Daskhan, G.C., Schmidt, E.N., Dworsky-Fried, Z., Giuliani, F., et al. (2022). Anti-inflammatory role of GM1 and other gangliosides on microglia. J. Neuroinflammation *19*, 9. https://doi.org/10.1186/s12974-021-02374-x.

Göttle, P., Förster, M., Weyers, V., Küry, P., Rejdak, K., Hartung, H.P., and Kremer, D. (2019). An unmet clinical need: roads to remyelination in MS. Neurol. Res. Pract. *1*, 21. https://doi.org/10.1186/s42466-019-0026-0.

Heß, K., Starost, L., Kieran, N.W., Thomas, C., Vincenten, M.C.J., Antel, J., Martino, G., Huitinga, I., Healy, L., and Kuhlmann, T. (2020). Lesion stage-dependent causes for impaired remyelination in MS. Acta Neuropathol. *140*, 359–375. https://doi.org/10.1007/s00401-020-02189-9.

Hill, R.A., Patel, K.D., Medved, J., Reiss, A.M., and Nishiyama, A. (2013). NG2 cells in white matter but not gray matter proliferate in response to PDGF. J. Neurosci. *33*, 14558–14566. https://doi.org/10.1523/jneurosci.2001-12.2013.

Huang, D., Shi, F.D., Jung, S., Pien, G.C., Wang, J., Salazar-Mather, T.P., He, T.T., Weaver, J.T., Ljunggren, H.G., Biron, C.A., et al. (2006). The neuronal chemokine CX3CL1/fractalkine selectively recruits NK cells that modify experimental autoimmune encephalomyelitis within the central nervous system. Faseb. J. *20*, 896–905. https://doi.org/10.1096/fj.05-5465com.

Inoue, K., Morimoto, H., Ohgidani, M., and Ueki, T. (2021). Modulation of inflammatory responses by fractalkine signaling in microglia. PLoS One *16*. e0252118. https://doi.org/10.1371/journal.pone.0252118.

Jablonska, B., Aguirre, A., Raymond, M., Szabo, G., Kitabatake, Y., Sailor, K.A., Ming, G.L., Song, H., and Gallo, V. (2010). Chordin-induced lineage plasticity of adult SVZ neuroblasts after demyelination. Nat. Neurosci. *13*, 541–550. https://doi.org/10.1038/nn.2536.

Jäkel, S., Agirre, E., Mendanha Falcão, A., van Bruggen, D., Lee, K.W., Knuesel, I., Malhotra, D., ffrench-Constant, C., Williams, A., and Castelo-Branco, G. (2019). Altered human oligodendrocyte heterogeneity in multiple sclerosis. Nature *566*, 543–547. https://doi.org/10.1038/s41586-019-0903-2.

Kang, S.H., Fukaya, M., Yang, J.K., Rothstein, J.D., and Bergles, D.E. (2010). NG2+ CNS glial progenitors remain committed to the oligodendrocyte lineage in postnatal life and following neurodegeneration. Neuron *68*, 668–681. https://doi.org/10.1016/j.neuron.2010.09.009.

Klein, B., Mrowetz, H., Barker, C.M., Lange, S., Rivera, F.J., and Aigner, L. (2018). Age influences microglial activation after cuprizone-induced demyelination. Front. Aging Neurosci. *10*, 278. https://doi.org/10.3389/fnagi.2018.00278.

Kosaraju, J., Seegobin, M., Gouveia, A., Syal, C., Sarma, S.N., Lu, K.J., Ilin, J., He, L., Wondisford, F.E., Lagace, D., et al. (2020). Metformin promotes CNS remyelination and improves social interaction following focal demyelination through CBP Ser436 phosphorylation. Exp. Neurol. *334*, 113454. https://doi.org/10.1016/j.expneurol.2020.113454.

Krupinski, J., Issa, R., Bujny, T., Slevin, M., Kumar, P., Kumar, S., and Kaluza, J. (1997). A putative role for platelet-derived growth factor in

angiogenesis and neuroprotection after ischemic stroke in humans. Stroke *28*, 564–573. https://doi.org/10.1161/01.str.28.3.564.

Lampron, A., Larochelle, A., Laflamme, N., Préfontaine, P., Plante, M.M., Sánchez, M.G., Yong, V.W., Stys, P.K., Tremblay, M.È., and Rivest, S. (2015). Inefficient clearance of myelin debris by microglia impairs remyelinating processes. J. Exp. Med. *212*, 481–495. https://doi.org/10.1084/jem.20141656.

Lassmann, H. (2003). Axonal injury in multiple sclerosis. J. Neurol. Neurosurg. Psychiatry *74*, 695–697. https://doi.org/10.1136/jnnp.74.6.695.

Li, Y., Dittmann, N.L., Eve, A., Watson, S., de Almeida, M.M.A., Footz, T., and Voronova, A. (2022). Hepatoma derived growth factor enhances oligodendrocyte genesis from subventricular zone precursor cells. ASN Neuro *14*. 17590914221086340. https://doi.org/10.1177/17590914221086340.

Li, Z., He, Y., Fan, S., and Sun, B. (2015). Clemastine rescues behavioral changes and enhances remyelination in the cuprizone mouse model of demyelination. Neurosci. Bull. *31*, 617–625. https://doi.org/10.1007/s12264-015-1555-3.

Lyons, A., Lynch, A.M., Downer, E.J., Hanley, R., O'Sullivan, J.B., Smith, A., and Lynch, M.A. (2009). Fractalkine-induced activation of the phosphatidylinositol-3 kinase pathway attentuates microglial activation in vivo and in vitro. J. Neurochem. *110*, 1547–1556. https://doi.org/10.1111/j.1471-4159.2009.06253.x.

McMurran, C.E., Jones, C.A., Fitzgerald, D.C., and Franklin, R.J.M. (2016). CNS remyelination and the innate immune system. Front. Cell Dev. Biol. *4*, 38. https://doi.org/10.3389/fcell.2016.00038.

Mendiola, A.S., Church, K.A., Cardona, S.M., Vanegas, D., Garcia, S.A., Macklin, W., Lira, S.A., Ransohoff, R.M., Kokovay, E., Lin, C.H.A., et al. (2022). Defective fractalkine-CX3CR1 signaling aggravates neuroinflammation and affects recovery from cuprizone-induced demyelination. J. Neurochem. *162*, 430–443. https://doi.org/10.1111/jnc.15616.

Miron, V.E., Boyd, A., Zhao, J.W., Yuen, T.J., Ruckh, J.M., Shadrach, J.L., van Wijngaarden, P., Wagers, A.J., Williams, A., Franklin, R.J.M., et al. (2013). M2 microglia and macrophages drive oligodendrocyte differentiation during CNS remyelination. Nat. Neurosci. *16*, 1211–1218. https://doi.org/10.1038/nn.3469.

Morganti, J.M., Nash, K.R., Grimmig, B.A., Ranjit, S., Small, B., Bickford, P.C., and Gemma, C. (2012). The soluble isoform of CX3CL1 is necessary for neuroprotection in a mouse model of Parkinson's disease. J. Neurosci. *32*, 14592–14601. https://doi.org/10.1523/jneurosci.0539-12.2012.

Muzumdar, M.D., Tasic, B., Miyamichi, K., Li, L., and Luo, L. (2007). A global double-fluorescent Cre reporter mouse. Genesis *45*, 593–605. https://doi.org/10.1002/dvg.20335.

Nait-Oumesmar, B., Decker, L., Lachapelle, F., Avellana-Adalid, V., Bachelin, C., and Baron-Van Evercooren, A. (1999). Progenitor cells of the adult mouse subventricular zone proliferate, migrate and differentiate into oligodendrocytes after demyelination. Eur. J. Neurosci. *11*, 4357–4366. https://doi.org/10.1046/j.1460-9568.1999.00873.x.

Nemes-Baran, A.D., White, D.R., and DeSilva, T.M. (2020). Fractalkine-Dependent microglial pruning of viable oligodendrocyte progenitor cells regulates myelination. Cell Rep. *32*, 108047. https://doi.org/10.1016/j.celrep.2020.108047.

O'Sullivan, S.A., and Dev, K.K. (2017). The chemokine fractalkine (CX3CL1) attenuates H2O2-induced demyelination in cerebellar slices. J. Neuroinflammation *14*, 159. https://doi.org/10.1186/s12974-017-0932-4.

Pabon, M.M., Bachstetter, A.D., Hudson, C.E., Gemma, C., and Bickford, P.C. (2011). CX3CL1 reduces neurotoxicity and microglial activation in a rat model of Parkinson's disease. J. Neuroinflammation *8*, 9. https://doi.org/10.1186/1742-2094-8-9.

Peters, A. (2009). The effects of normal aging on myelinated nerve fibers in monkey central nervous system. Front. Neuroanat. *3*, 11. https://doi.org/10.3389/neuro.05.011.2009.

Ridderstad Wollberg, A., Ericsson-Dahlstrand, A., Juréus, A., Ekerot, P., Simon, S., Nilsson, M., Wiklund, S.J., Berg, A.L., Ferm, M., Sunnemark, D., and Johansson, R. (2014). Pharmacological inhibition of the chemokine receptor CX3CR1 attenuates disease in a chronic-relapsing rat model for multiple sclerosis. Proc. Natl. Acad. Sci. USA *111*, 5409–5414. https://doi.org/10.1073/pnas.1316510111.

Sherafat, A., Pfeiffer, F., and Nishiyama, A. (2021a). Shaping of regional differences in oligodendrocyte dynamics by regional heterogeneity of the pericellular microenvironment. Front. Cell. Neurosci. *15*, 721376. https://doi.org/10.3389/fncel.2021.721376.

Sherafat, A., Pfeiffer, F., Reiss, A.M., Wood, W.M., and Nishiyama, A. (2021b). Microglial neuropilin-1 promotes oligodendrocyte expansion during development and remyelination by trans-activating platelet-derived growth factor receptor. Nat. Commun. *12*, 2265. https://doi.org/10.1038/s41467-021-22532-2.

Simons, M., and Nave, K.A. (2015). Oligodendrocytes: myelination and axonal support. Cold Spring Harb. Perspect. Biol. *8*. a020479. https://doi.org/10.1101/cshperspect.a020479.

Snaidero, N., Velte, C., Myllykoski, M., Raasakka, A., Ignatev, A., Werner, H.B., Erwig, M.S., Möbius, W., Kursula, P., Nave, K.A., and Simons, M. (2017). Antagonistic functions of MBP and CNP establish cytosolic channels in CNS myelin. Cell Rep. *18*, 314–323. https://doi.org/10.1016/j.celrep.2016.12.053.

Srinivas, S., Watanabe, T., Lin, C.S., William, C.M., Tanabe, Y., Jessell, T.M., and Costantini, F. (2001). Cre reporter strains produced by targeted insertion of EYFP and ECFP into the ROSA26 locus. BMC Dev. Biol. *1*, 4. https://doi.org/10.1186/1471-213x-1-4.

Voronova, A., Yuzwa, S.A., Wang, B.S., Zahr, S., Syal, C., Wang, J., Kaplan, D.R., and Miller, F.D. (2017). Migrating interneurons secrete fractalkine to promote oligodendrocyte formation in the developing mammalian brain. Neuron *94*, 500–516.e9. https://doi.org/10.1016/j.neuron.2017.04.018.

Wang, J., Pan, H., Lin, Z., Xiong, C., Wei, C., Li, H., Tong, F., and Dong, X. (2021). Neuroprotective effect of fractalkine on radiation-induced brain injury through promoting the M2 polarization of microglia. Mol. Neurobiol. *58*, 1074–1087. https://doi.org/10.1007/s12035-020-02138-3.

Watson, A.E.S., de Almeida, M.M.A., Dittmann, N.L., Li, Y., Torabi, P., Footz, T., Vetere, G., Galleguillos, D., Sipione, S., Cardona, A.E., et al. (2021). Fractalkine signaling regulates oligodendroglial cell genesis from SVZ precursor cells. Stem Cell Rep. *16*, 1968–1984. https://doi.org/10.1016/j.stemcr.2021.06.010.

Watson, A.E.S., Goodkey, K., Footz, T., and Voronova, A. (2020). Regulation of CNS precursor function by neuronal chemokines.

Neurosci. Lett. *715*, 134533. https://doi.org/10.1016/j.neulet.2019.134533.

Xing, Y.L., Röth, P.T., Stratton, J.A.S., Chuang, B.H.A., Danne, J., Ellis, S.L., Ng, S.W., Kilpatrick, T.J., and Merson, T.D. (2014). Adult neural precursor cells from the subventricular zone contribute significantly to oligodendrocyte regeneration and remyelination. J. Neurosci. *34*, 14128–14146. https://doi.org/10.1523/jneurosci.3491-13.2014.

Yeung, M.S.Y., Djelloul, M., Steiner, E., Bernard, S., Salehpour, M., Possnert, G., Brundin, L., and Frisén, J. (2019). Dynamics of oligodendrocyte generation in multiple sclerosis. Nature *566*, 538–542. https://doi.org/10.1038/s41586-018-0842-3.

Zhang, S.Z., Wang, Q.Q., Yang, Q.Q., Gu, H.Y., Yin, Y.Q., Li, Y.D., Hou, J.C., Chen, R., Sun, Q.Q., Sun, Y.F., et al. (2019). NG2 glia regulate brain innate immunity via TGF-beta2/TGFBR2 axis. BMC Med. *17*, 204. https://doi.org/10.1186/s12916-019-1439-x.

Stem Cell Reports, Volume *18*

Supplemental Information

Fractalkine enhances oligodendrocyte regeneration and remyelination in a demyelination mouse model

Monique M.A. de Almeida, Adrianne E.S. Watson, Sana Bibi, Nicole L. Dittmann, Kara Goodkey, Pedram Sharafodinzadeh, Danny Galleguillos, Maryam Nakhaei-Nejad, Jayasankar Kosaraju, Noam Steinberg, Beatrix S. Wang, Tim Footz, Fabrizio Giuliani, Jing Wang, Simonetta Sipione, Julia M. Edgar, and Anastassia Voronova

Figure S1

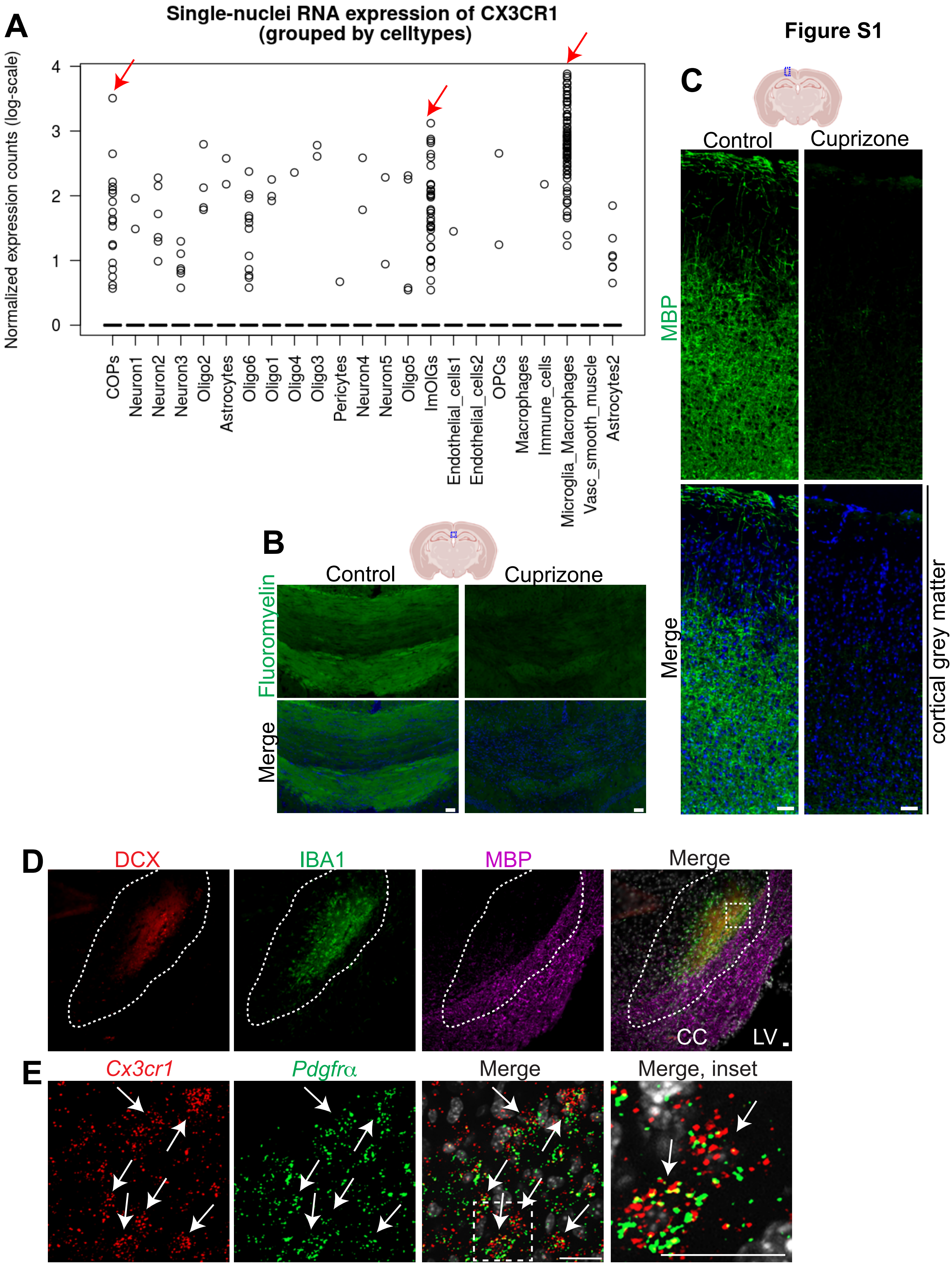

A
Single-nuclei RNA expression of CX3CR1
(grouped by celltypes)
Normalized expression counts (log-scale)
0
1
2
3
4
COPs
Neuron1
Neuron2
Neuron3
Oligo2
Astrocytes
Oligo6
Oligo1
Oligo4
Oligo3
Pericytes
Neuron4
Neuron5
Oligo5
ImOlGs
Endothelial_cells1
Endothelial_cells2
OPCs
Macrophages
Immune_cells
Microglia_Macrophages
Vasc_smooth_muscle
Astrocytes2
B
Control
Cuprizone
Fluoromyelin
Merge
C
Control
Cuprizone
MBP
Merge
cortical grey matter
D
DCX
IBA1
MBP
Merge
CC
LV
E
Cx3cr1
Pdgfrα
Merge
Merge, inset

Figure S1. Related to Figure 1. ***Cx3cr1 mRNA is expressed in OPCs and microglia/macrophages.*** **A.** *CX3CR1* mRNA expression extracted from single nuclei RNA sequencing from brain white matter of individuals with progressive MS (Jäkel et al., 2019), https://castelobranco.shinyapps.io/MSCtrl_CCA_18/). ImOLGs=Immune oligodendroglia; Oligo = Oligodendrocyte cluster; cOPC=committed OPC. Red arrows designate cell clusters with enriched *CX3CR1* mRNA expression. **B-C**. Representative images of mice fed normal chow (control, left) and demyelinated mice subjected to cuprizone chow for 6 weeks (cuprizone, right). Myelin is detected with Fluoromyelin stain in corpus callosum (green, **B**) and MBP in cortical grey matter (green, **C**). Images counter-stained with Hoechst 33258 (blue) and are representative of areas analyzed throughout the paper when referring to midline corpus callosum and cortical grey matter. **D.** Representative image of 1-month old mouse injected with lysolethicin into corpus callosum and immunostained for DCX (red), IBA1 (green) and MBP (purple). Dashed lines indicate demyelination and DCX/IBA1 cell infiltration boundary. **E**. Double-label RNA scope analysis of lysolethicin-demyelinated corpus callosum for *Cx3cr1* (red) and *Pdgfr*α (green) mRNAs. Approximate location of image is shown in hatched box in C in Merge panel. Arrows indicate *Cxc3r1*-postive, *Pdgfr*α-positive cells. Inset in hatched box is shown on the right side. Scale bars are 50 µm in **B-C** and 20 µm in **D-E**. LV = lateral ventricle.

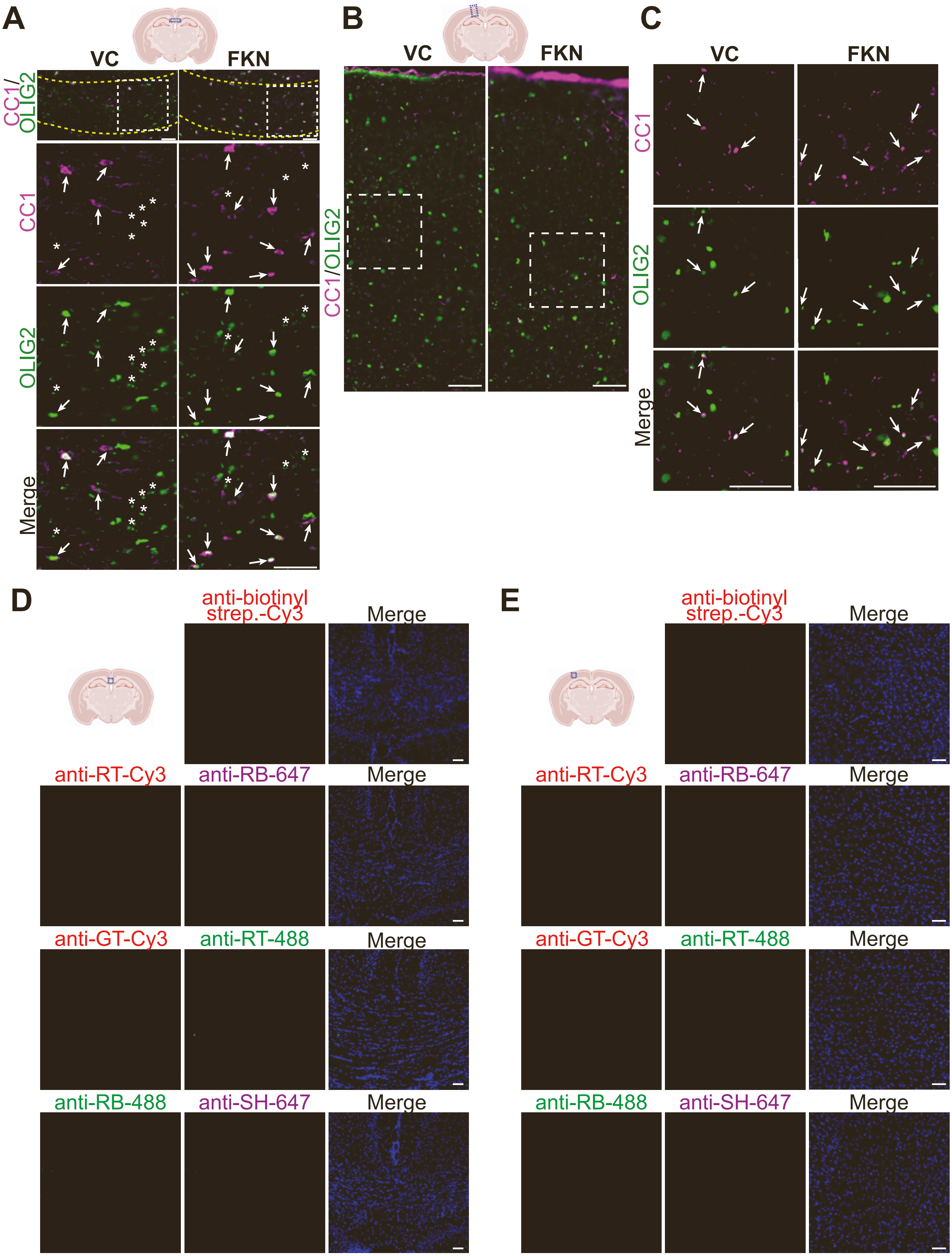
A
VC
FKN
CC1/OLIG2
CC1
OLIG2
Merge
B
VC
FKN
CC1/OLIG2
C
VC
FKN
CC1
OLIG2
Merge
D
anti-biotinyl strep.-Cy3
Merge
anti-RT-Cy3
anti-RB-647
Merge
anti-GT-Cy3
anti-RT-488
Merge
anti-RB-488
anti-SH-647
Merge
E
anti-biotinyl strep.-Cy3
Merge
anti-RT-Cy3
anti-RB-647
Merge
anti-GT-Cy3
anti-RT-488
Merge
anti-RB-488
anti-SH-647
Merge

Figure S2. Related to Figure 2. ***FKN infusion increases oligodendrocyte density after demyelination.*** **A**. Top: representative images of midline corpus callosum immunostained for CC1 (magenta) and OLIG2 (green) from demyelinated VC- and FKN-infused mice (please see Fig. 2A for experimental design). Approximate location is indicated by blue rectangle in the brain schematic. Dashed squares from top are shown at higher magnification in the bottom panels. Arrows indicate double-positive CC1+OLIG2+ oligodendrocytes, and asterisks OLIG2+ punctate staining (please see Fig. 2H for more details). Marker+ cells were quantified in Fig. 2C-D in the area between yellow hatched lines. Scale bar: 50 µm. **B-C.** Representative images of cortical column (**B**) immunostained for CC1 (magenta) and OLIG2 (green) from VC- and FKN-infused mice. Approximate location is indicated by blue rectangle in the brain schematic. Dashed squares from **B** are shown at higher magnification in **C**. Arrows indicate double-positive CC1+OLIG2+ oligodendrocytes. Scale bar: 200 µm. **D-E.** Secondary antibody controls in medial midline corpus callosum (**D**) and cortical grey matter (**E**). Brain sections from mice treated with cuprizone chow for 6 weeks were returned for 3 (**D**) and 7 (**E**) days to normal chow. Resulting brain sections were processed and stained as described as in the "Immunohistochemistry" section in Materials and Methods with the exclusion of primary antibodies. Sections were counter-stained with Hoechst 33258 (blue; "Merge" panel). Approximate location is indicated by blue rectangle in the brain schematics. GT = goat, RB = rabbit, RT = rat, SH = sheep, Strep = streptavidin. Scale bars are 50 µm.

Figure S3

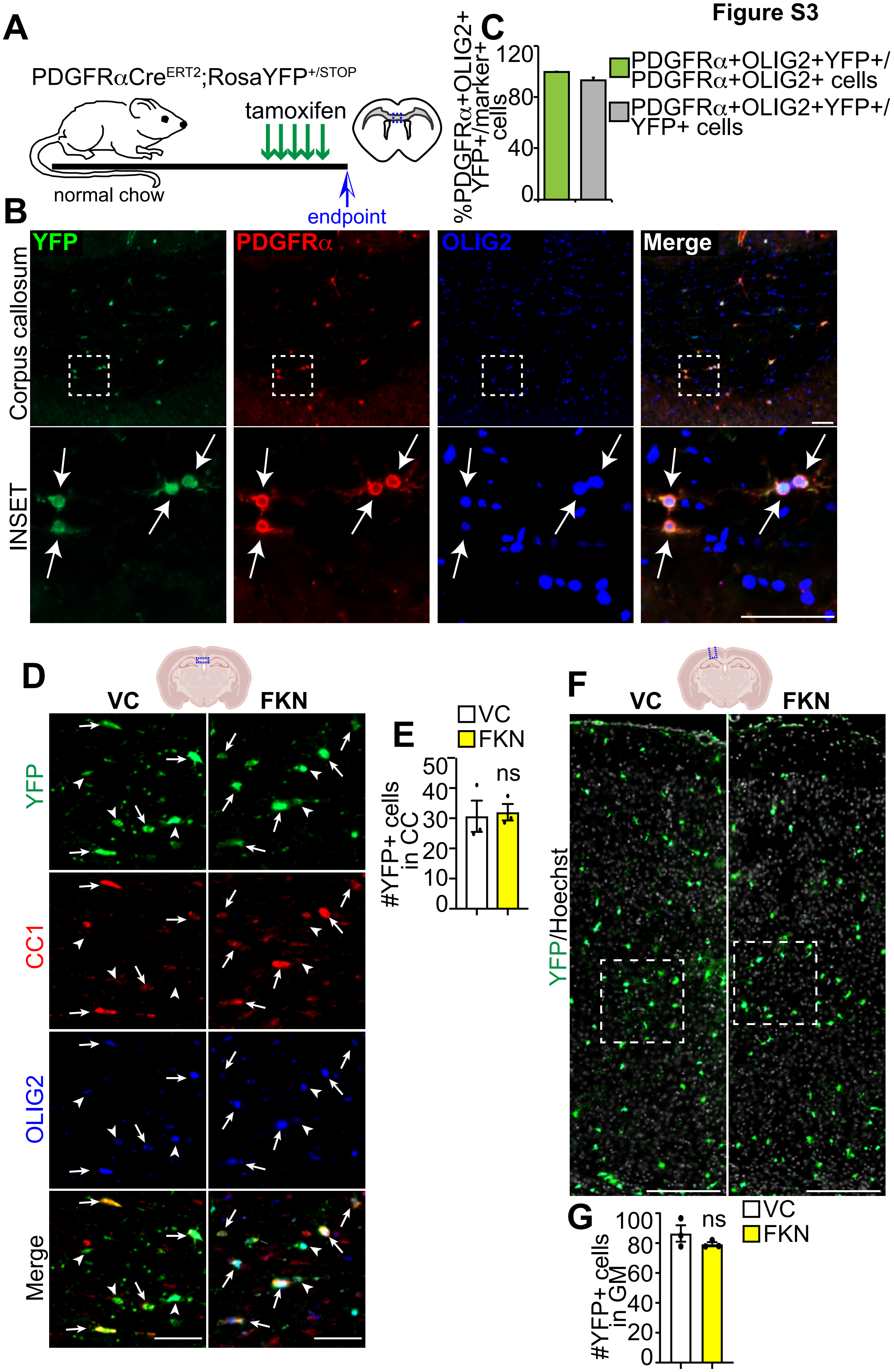
A
PDGFRαCre[ERT2];RosaYFP[+/STOP]
tamoxifen
normal chow
endpoint
B
YFP
PDGFRα
OLIG2
Merge
Corpus callosum
INSET
C
%PDGFRα+OLIG2+ YFP+/marker+ cells
120
80
40
0
PDGFRα+OLIG2+YFP+/ PDGFRα+OLIG2+ cells
PDGFRα+OLIG2+YFP+/ YFP+ cells
D
VC
FKN
YFP
CC1
OLIG2
Merge
E
VC
FKN
#YFP+ cells in CC
50
40
30
20
10
0
ns
F
VC
FKN
YFP/Hoechst
G
#YFP+ cells in GM
100
80
60
40
20
0
ns
VC
FKN

Figure S3. Related to Figure 3. ***FKN infusion increases de novo oligodendrocyte formation after demyelination*** **A**. Schematic illustration of **B**-**C**. 10 week-old PDGFRαCre^{ERT2};RosaYFP$^{STOP/+}$ were injected with tamoxifen once daily for 5 days. 72 hours later, mice were perfused. **B.** Representative images of midline corpus callosum immunostained for YFP (green), PDGFRα (red) and OLIG2 (blue). Approximate location is indicated by blue rectangle in the brain schematic. Arrows indicate triple-positive YFP+PDGFRα+OLIG2+ recombined OPCs. Scale bar: 50 µm. **C.** Quantification of **B** for % PDGFRα+OLIG2+YFP+/PDGFRα+OLIG2+ cells (green bar) and % PDGFRα+OLIG2+YFP+/YFP+ cells (grey bar). n=3 mice, error bars represent SEM. **D.** Representative images of midline corpus callosum immunostained for YFP (green), CC1 (red) and OLIG2 (blue) from remyelinating VC- and FKN-infused PDGFRαCre^{ERT2};RosaYFP$^{STOP/+}$ mice (please see Fig. 3C for experimental design). Approximate location is indicated by blue rectangle in the brain schematic. Arrows indicate triple-positive YFP+CC1+OLIG2+ newborn oligodendrocytes. Arrowheads indicate YFP+CC1-OLIG2+ cells. Scale bar: 50 µm. **E.** Quantification of Fig S3D for average number of YFP+ cells per section in the medial midline corpus callosum. * $p<0.05$. n=3 mice per group from at least 2 independent litters **F.** Representative images of cortical grey matter column immunostained for YFP (green) and counterstained with Hoechst 33258 (white) from remyelinating VC- and FKN-infused PDGFRαCre^{ERT2};RosaYFP$^{STOP/+}$ mice (please see Fig. 3C for experimental design). Approximate location is indicated by a blue rectangle in the brain schematic. Dashed squares are shown at higher magnification in Fig. 3K. **G.** Quantification of **F** for average number of YFP+ cells per section. n=3 mice per group from at least 2 independent litters. 3-5 anatomically matched sections per brain were analyzed with at least 300 cells counted per brain. For all graphs error bars represent SEM. Data was analyzed using unpaired t-test.

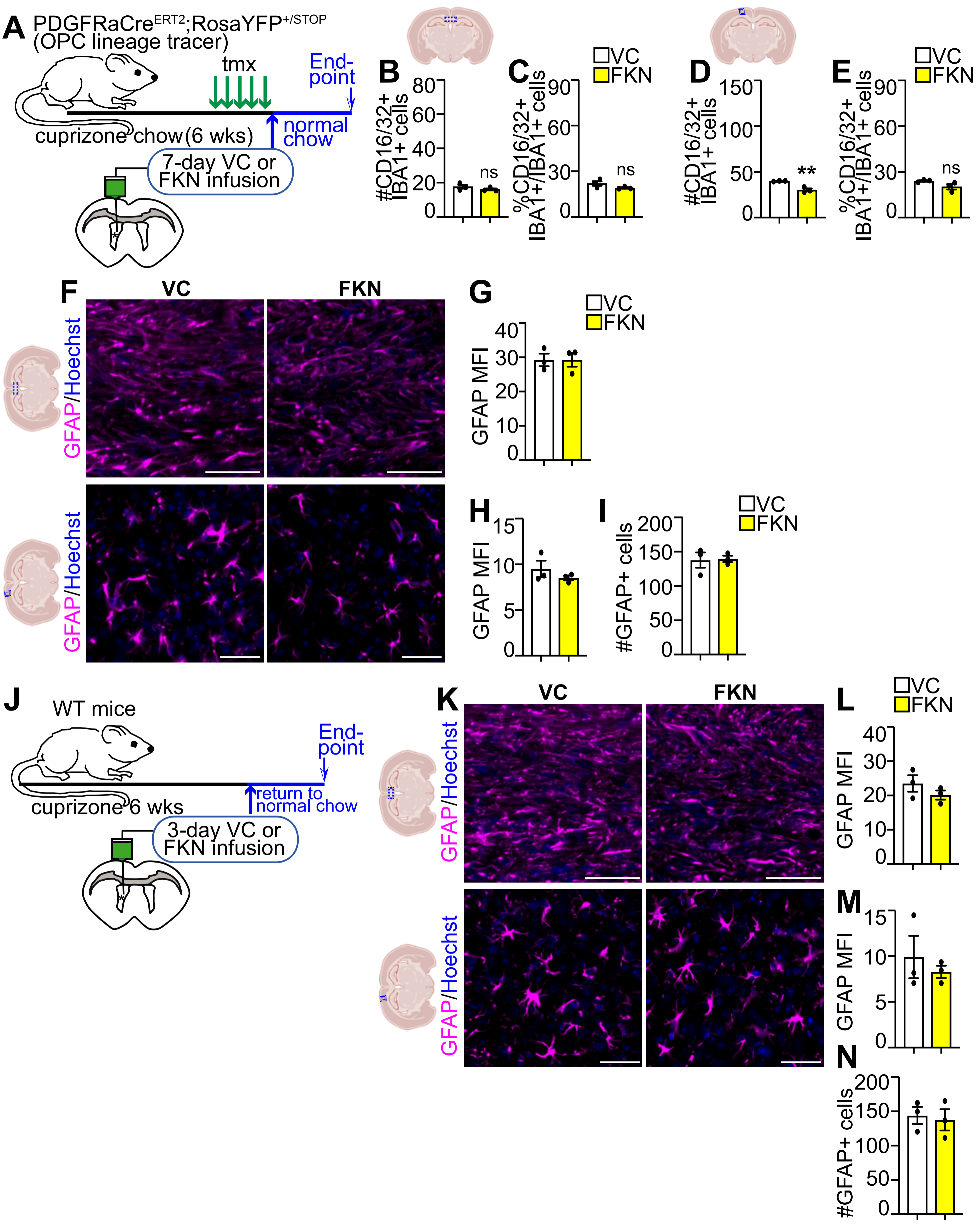
A
PDGFRaCreERT2;RosaYFP+/STOP
(OPC lineage tracer)
tmx
End-point
normal chow
cuprizone chow (6 wks)
7-day VC or FKN infusion
B
#CD16/32+ IBA1+ cells
ns
C
%CD16/32+ IBA1+/IBA1+ cells
ns
VC
FKN
D
#CD16/32+ IBA1+ cells
**
E
%CD16/32+ IBA1+/IBA1+ cells
ns
F
VC
FKN
GFAP/Hoechst
G
GFAP MFI
H
GFAP MFI
I
#GFAP+ cells
J
WT mice
End-point
return to normal chow
cuprizone 6 wks
3-day VC or FKN infusion
K
VC
FKN
GFAP/Hoechst
L
GFAP MFI
M
GFAP MFI
N
#GFAP+ cells

Figure S4. Related to Figure 5. ***FKN does not affect astrocyte activation or density.*** **A-I.** Analysis of microglia and astrocyte activation in 7-day infused remyelinating mice. **A.** Schematic illustration of experiment. 10 week-old PDGFRαCre^{ERT2};RosaYFP$^{STOP/+}$ were demyelinated with cuprizone chow for 6 weeks. In the last week of cuprizone treatment, mice were injected with tamoxifen once daily for 5 days. 72 hours later, intracerebral ventricular (ICV) surgery was performed. FKN or VC was infused unilaterally into the lateral ventricle (indicated by asterisk) for 7 days via osmotic mini-pump. After ICV surgery, mice were returned to normal chow for recovery. **B-E.** Analysis of average number of CD16/32+IBA1+ cells (**B, D**) and the proportion of IBA1+CD16/32+ cells (%IBA1+CD16/32+/IBA1+ cells **C, E**) in midline corpus callosum (**B-C**) and cortical grey matter (**D-E**). Approximate location of analyzed images is depicted as blue rectangles in brain schematics. ** $p<0.01$, ns = not significant. n=3 mice per group from at least 2 independent litters. **F.** Representative images of midline corpus callosum (top row) and cortical grey matter (bottom row) immunostained for GFAP (magenta) and counterstained with Hoechst 33258 (blue) from VC- and FKN-infused mice. Approximate location is indicated by blue rectangle in the brain schematic. Scale bars: 50 µm. **G-H.** Analysis of **F** for mean fluorescence intensity (MFI) of GFAP signal in corpus callosum (**G**) and cortical grey matter (**H**). **I**. Quantification of **F** for average number of GFAP+ cells in cortical grey matter of VC and FKN-infused mice. n=3 mice per group from at least 2 independent litters. **J-N.** Analysis of astrocyte activation in 3-day infused remyelinating mice **J.** Schematic illustration of experiment. 10 week-old WT C57BL/6J mice were demyelinated with cuprizone-chow administration for 6 weeks. At this point, FKN or VC was infused unilaterally into the lateral ventricle (indicated by asterisk) for 3 days via osmotic mini-pump. After intracerebral ventricular (ICV) surgery, mice were returned to normal chow for recovery. **K**. Representative images of midline corpus callosum (top row) and cortical grey matter (bottom row) immunostained for GFAP (magenta) and counterstained with Hoechst 33258 (blue) from VC- and FKN-infused mice. Approximate location is indicated by blue rectangle in the brain schematic. Scale bars: 50 µm. **L-M.** Analysis of **K** for mean fluorescence intensity (MFI) of GFAP signal in corpus callosum (**L**) and cortical grey matter (**M**). **N**. Quantification of **K** for average number of GFAP+ cells in cortical grey matter of VC and FKN-infused mice. n=3 mice per group from at least 2 independent litters. Data were analyzed using unpaired t-test. All error bars represent SEM.

Figure S5

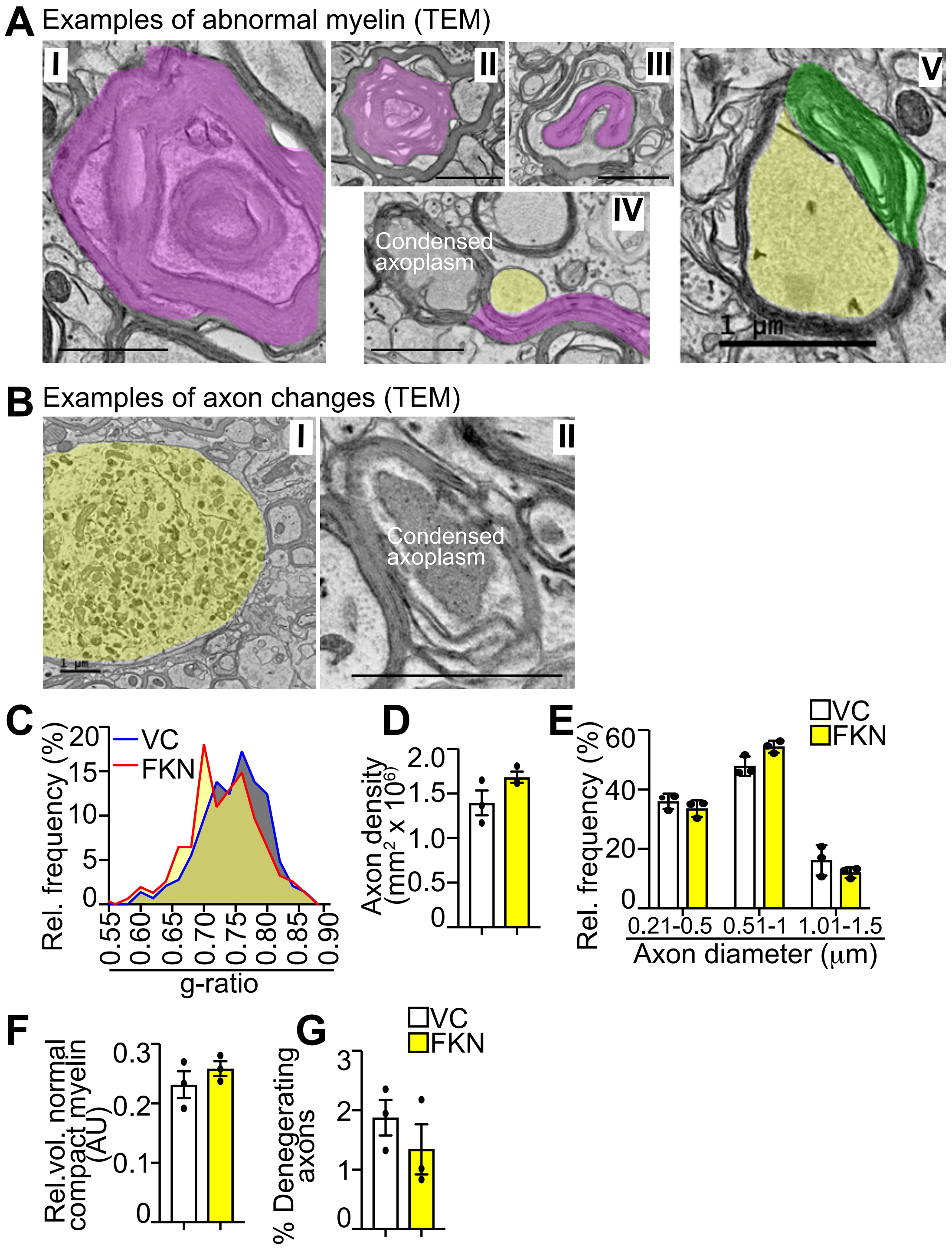
A Examples of abnormal myelin (TEM)
I
II
III
IV
V
Condensed axoplasm
1 µm
B Examples of axon changes (TEM)
I
II
Condensed axoplasm
1 µm
C
VC
FKN
Rel. frequency (%)
g-ratio
0.55
0.60
0.65
0.70
0.75
0.80
0.85
0.90
D
Axon density (mm² x 10⁶)
E
Rel. frequency (%)
Axon diameter (µm)
0.21-0.5
0.51-1
1.01-1.5
VC
FKN
F
Rel.vol. normal compact myelin (AU)
G
% Denegerating axons
VC
FKN

Figure S5. Related to Figure 6. ***Examples of abnormal myelin and axon changes.*** **A.** Representative TEM images from VC-infused (micrographs in I, III-V) or FKN-infused (micrograph in II) mice illustrating redundant (purple) and split (green) abnormal myelin. **B**. Representative TEM images from VC-infused (micrograph in I) or FKN-infused (micrograph in II) mice illustrating swollen (yellow with filled organelles) and condensed (dark grey) abnormal axoplasm. Scale bars: 1 μm. **C-E**. Analysis of Fig. 6B for g-ratio frequency distribution (**C**), average total axon density (**D**, each data point represents a mouse), relative axon frequency within 0.21-0.5, 0.51-1 and 1.01-1.5 μm diameter (**E**, each data point represents a mouse). **F-G**. Morphometric analysis of **A** for normal compact myelin (**F**, each data point represents a mouse) and of **B** for degenerating axons (**G**, each data point represents a mouse). n=3 mice per group from at least 2 independent litters. For g-ratio analysis, at least 100 axons from 8 images per sample were analyzed. For morphometric analysis, 30 images per sample were analyzed. For all bar graphs error bars represent SEM. Data were analyzed using unpaired t-test, except data in **E** were analyzed with multiple t-test.

Figure S6

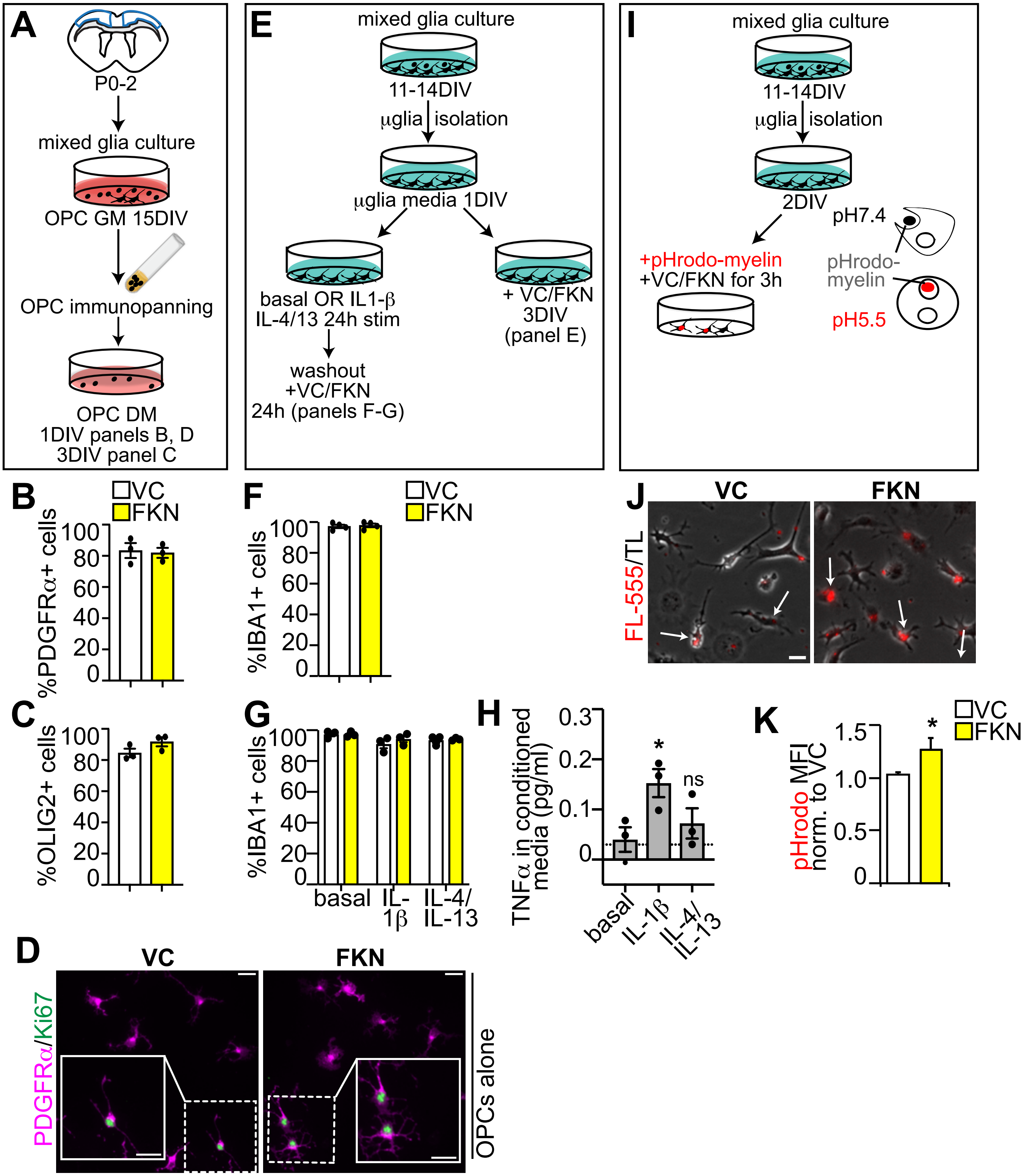

A
P0-2
mixed glia culture
OPC GM 15DIV
OPC immunopanning
OPC DM
1DIV panels B, D
3DIV panel C
E
mixed glia culture
11-14DIV
μglia isolation
μglia media 1DIV
basal OR IL1-β
IL-4/13 24h stim
washout
+VC/FKN
24h (panels F-G)
+ VC/FKN
3DIV
(panel E)
I
mixed glia culture
11-14DIV
μglia isolation
2DIV
+pHrodo-myelin
+VC/FKN for 3h
pH7.4
pHrodo-
myelin
pH5.5
B
VC
FKN
%PDGFRα+ cells
100
80
60
40
20
0
F
VC
FKN
%IBA1+ cells
J
VC
FKN
FL-555/TL
C
%OLIG2+ cells
G
%IBA1+ cells
basal
IL-1β
IL-4/
IL-13
H
TNFα in conditioned media (pg/ml)
0.3
0.2
0.1
0
*
ns
basal
IL-1β
IL-4/
IL-13
K
VC
FKN
pHrodo MFI
norm. to VC
1.5
1.0
0.5
0
*
D
VC
FKN
PDGFRα/Ki67
OPCs alone

Figure S6. Related to Figure 7. ***FKN increases myelin debris phagocytosis by microglia in vitro.*** **A.** Schematic illustration of **B-D**. Cortices from P0-P2 CD1 pups were cultured as mixed glia in OPC GM (growth media). After 15 days OPC were isolated by immunopanning using PDGFRα+ magnetic beads (please see Experimental Procedures for more details). OPCs were cultured for 1-3 DIV in OPC DM (differentiation media). **B**. Quantification of % PDGFRα+ cells in OPC cultures treated with VC/FKN for 1DIV. n=3 biological replicates. **C**. Quantification of % OLIG2+ cells in OPC cultures treated with VC/FKN for 3DIV. n=3 biological replicates. **D**. Representative images of OPCs cultured in the absence of microglia and presence of VC or 250 ng/ml FKN for 1DIV and immunostained for PDGFRα (magenta) and Ki67 (green). Insets are shown at higher magnification and demonstrate PDGFRα+Ki67+ OPCs. Scale bar: 20 μm. **E.** Schematic illustration of **F-H**. Cortices from P0-P2 CD1 pups were cultured as mixed glia in microglia media. After 11-14 days, microglia were isolated by shaking. Microglia were then pre-stimulated with IL-1β and IL-4/IL-13 cytokines or no cytokines (basal conditions) for 24h and further treated with VC/FKN for additional 24h (results in panels **F-G**). Alternately, isolated microglia were cultured in microglia media with VC/FKN for 3DIV (results in panel **F**). **F**. Quantification of % IBA1+ cells in microglia cultures after 3DIV. n=3 biological replicates. **G**. Quantification of % IBA1+ cells in basal, IL-1β and IL-4/IL-13 pre-stimulated microglia cultures treated with VC or FKN for additional 24h. n=3 biological replicates. **H**. ELISA measurements of TNFα in media conditioned by basal, IL-1β and IL-4/IL-13 pre-stimulated microglia. Dashed line corresponds to media not exposed to microglia. Each data point corresponds to biological replicate. * $p<0.05$, ns = not significant. n=3 biological replicates. **I**. Schematic illustration of **J-K**. Cortices from P0-P2 CD1 pups were cultured as mixed glia in microglia media. After 11-14 days, microglia were isolated by shaking. Microglia were then cultured without FBS for 24h and further incubated with pHRodo-myelin and VC/FKN for additional 3h. **J**. Representative images of primary microglia (cells imaged with transmitted light [TL]) incubated with pHRodo-myelin (red fluorescence designated as FL-555) from VC- and FKN-treated cultures. Arrows designate microglia with pHRodo signal. Scale bars: 20 μm. **K**. Quantification of **J** for mean fluorescence intensity (MFI) of pHRodo signal normalized to VC. * $p<0.05$. n=4 biological replicates. Data were analyzed with paired t-test, except data in **G** were analyzed with multiple t-test and data in **H** were analyzed with one-way ANOVA followed by Dunnett’s post-hoc test. All error bars represent SEM.

Supplemental Experimental Procedures

MATERIALS AND METHODS

Growth factors and cytokines: Murine soluble FKN (CX3CL1) was obtained from R&D Systems (cat #458-MF), FKN-647 from Almac (cat #CAF-51-A-01, #CAF-14-A-03). BSA-647 was obtained from ThermoFisher (cat# A34785). For primary cell culture experiments, FKN was reconstituted in sterile 1X PBS at a concentration of 100 µg/mL and used at 250 ng/ml. For intracerebral ventricular (ICV) infusions, FKN-647 was reconstituted at 0.5 mg/mL in 0.1% BSA in 1X PBS, and unconjugated FKN was reconstituted at 16.7 ng/mL for 3-7 day infusion or 75.8 ng/mL for 21-day infusion in sterile 0.2% bovine serum albumin (BSA, Jackson ImmunoResearch) in 1x PBS and infused at 200 ng/day rate using Alzet osmotic mini-pumps as per (Watson et al., 2021). Murine IL-1β was obtained from Cedarlane (cat# CLCYT273), reconstituted in cell culture grade H_2O supplemented with 0.1% BSA at a concentration of 10 µg/ml and used at 10 ng/ml; mouse IL-4 was obtained from Miltenyi Biotec (cat#130-097-761) and reconstituted in cell culture grade H_2O at 10 µg/ml; mouse IL-13 was purchased from R&D Systems (cat#413ML/CF) and resuspended in D-PBS at 50µg/ml, IL-4 and IL-13 were used at 20 ng/ml; GM-CSF (granulocyte-macrophage colony-stimulating factor) was obtained from R&D Systems (cat# 415ML/CF), reconstituted at 100 µg/ml and used at 10 ng/ml; FGF was obtained from Peprotech (cat #100-18B), reconstituted in cell culture grade H_2O at 10 µg/ml and used at 10 ng/ml; PDGF-AA was obtained from R&D (cat# 1055-AA-050), reconstituted in sterile 4 mM HCl at 10 µg/ml and used at 10 ng/ml.

Experimental Model And Subject Details

Mice: Animal use protocols were approved by the Research Ethics Office at the University of Alberta and the University of Ottawa in accordance with the Canadian Council of Animal Care Policies. Mice from both sexes were used for all cell culture and *in vivo* experiments. PDGFRαCre^{ERT2} (B6N.Cg-Tg(Pdgfra-cre/ERT)467Dbe/J; RRID:IMSR_JAX:018280), RosaYFP^{STOP} (B6.129X1-Gt(ROSA)26Sortm1(EYFP)Cos/J; RRID:IMSR_JAX:006148), Rosa$^{mT/mG}$ (B6.129(Cg)-Gt(ROSA)26Sortm4(ACTB-tdTomato,-EGFP)Luo/J; RRID:IMSR_JAX:007676) and wild-type (WT) C57BL/6J mice were obtained from Jackson Laboratories (Kang et al., 2010; Muzumdar et al., 2007; Srinivas et al., 2001). Experimental mice were generated by mating PDGFRαCre^{ERT2}-positive hemizygous male with homozygous RosaYFP^{STOP} or Rosa$^{mT/mG}$ females. PDGFRαCre^{ERT2}-positive progeny were used in lineage tracing experiments. For some demyelination experiments as well as for RNA scope, WT animals were used as indicated in the figure legends. CD1 mice purchased from Charles River Laboratory were used for all primary cell culture experiments (developmental age: postnatal day [P] 0-2). For genotyping, the following primers were used: PDGFRαCre^{ERT2}: TCAGCCTTAAGCTGGGACAT (5'-3', forward), ATGTTTAGCTGGCCCAAATG (5'-3', reverse), RosaYFP^{STOP}: AAAGTCGCTCTGAGTTGTTAT (5'-3', common), GGAGCGGGAGAAATGGATATG (5'-3', wildtype), AAGACCGCGAAGAGTTTGTC, (5'-3', mutant), Rosa$^{mT/mG}$: CTTTAAGCCTGCCCAGAAGA (5'-3', common), AGGGAGCTGCAGTGGAGTAG (5'-3', wildtype), TAGAGCTTGCGGAACCCTTC (5'-3', mutant).

Cuprizone-induced demyelination experiments: For cuprizone-induced demyelination, 10-week old WT, PDGFRαCre^{ERT2};RosaYFP^{STOP} or PDGFRαCre^{ERT2};Rosa$^{mT/mG}$ mice were subjected to nutragel (Bio-Serv) containing 0.2% cuprizone (bis-cyclohexanone oxaldihydrazone, Sigma) for 6 weeks as described in (Saito et al., 2021). Animal weight and health was monitored

weekly. After 6 weeks of cuprizone chow, intracerebral ventricular surgery (ICV) was performed (please see details below), at which point mice were returned to normal chow for 3-21 days to allow remyelination.

Tamoxifen injections: PDGFRαCre^{ERT2};RosaYFP^{STOP} or PDGFRαCre^{ERT2};mT/mG animals were injected with 3 mg tamoxifen dissolved in 10% ethanol (Commercial Alcohols) and 90% sunflower seed oil (Sigma) daily for 5 days during last week of cuprizone treatment. 72h after last tamoxifen injection, intracerebral ventricular surgery (ICV) was performed. Normal (non-demyelinated) 10-week old PDGFRαCre^{ERT2}; RosaYFP^{STOP} mice were injected with tamoxifen as above. 72h after last tamoxifen injection, mice were perfused as per details below.

ICV infusions: For FKN-647 and BSA-647 one-time injection experiments, 2-3-month old wild-type C57/BL6J mice were used. For FKN infusion experiments, we used wild-type C57/BL6J, PDGFRαCre^{ERT2};RosaYFP^{STOP} or PDGFRαCre^{ERT2};mT/mG demyelinated with cuprizone as described above. Mice were anesthetized via inhalation of isoflurane and placed in a stereotaxic frame. Syringe needle (for one-time injection) or cannulas (Alzet, for multi-day infusion) were positioned after craniotomy for infusion into right ventricle using the following coordinates relative to bregma: -1.000 medio-lateral (ML), -0.300 anterior-posterior (AP), -2.500 dorso-ventral (DV). For one-time injection, 0.5-1 µl of FKN-647 or matched volume and equimolar amount of BSA-647 were injected once over a 10-20 min period as described in (Watson et al., 2021). For multi-day infusion, 7-day or 28-day osmotic mini-pumps (Alzet, 1007D or 1004) were connected containing VC (vehicle-control: 0.2% BSA in 1x PBS) or 16.7 ng/mL or 75.8 ng/mL FKN in VC. Overall, 200 ng of FKN was delivered over every 24h for 3-21 days. Cannula was secured to the skull with Loctite 454. After surgery, mice were returned to normal chow. BrdU (5-bromo-2'-deoxyuridine, Sigma) was injected at 100 mg/kg dose 24h before perfusions.

Lysolethicin (LPC) injections: LPC-mediated demyelination was performed as described in (Kosaraju et al., 2020). Briefly, one-month old WT mice were injected with LPC (Sigma, L1381) (1 µl of 1% solution in 1xPBS) using a stereotactic apparatus at two sites: +1.0 mm AP, +1.0 mm ML, −2.2 mm DV and + 0.7 mm AP, +1.0ML, −2.2 DV. After injection, needle was held in place for 3 min to reduce back-flow. Animals were allowed to recover for 14 days.

Myelin isolation: Myelin was isolated as described in (Bhattacherjee et al., 2019). Briefly, brains from 5-month old Sprague Dawley rats were minced and homogenized in ice-cold 0.3 M sucrose. The homogenate was layered over 0.83 M sucrose and ultracentrifuged for 45 min at 75,000 g. The crude myelin was extracted, homogenized and ultracentrifuged twice more (12,000 g, 15 and 10 min, 4°C). Myelin was further purified by another density gradient and subjected to Tris·Cl buffer osmotic shock. Myelin was then resuspended in 100 mM $NaHCO_3$, pH 8.5 at 100 mg/ml concentration and incubated with 10 µM of pHRodo dye (Invitrogen) for 1h at room temperature. The excess dye was washed out by washing myelin in 1xPBS four times. The labeled myelin was stored in 1xPBS containing 1% DMSO as single-use aliquots at −80°C.

Tissue dissection and collection: Mice under 21 days of age were euthanized with CO_2. Dissected brains were kept in ice-cold Hank's Balanced Salt Solution (HBSS, Gibco) until dissections and subsequent primary cultures. Mice over 21 days of age were anesthetized with 102 mg/kg of body weight Euthansol (WDDC) and perfused transcardially with HBSS, followed by 4% paraformaldehyde in 1X PBS (PFA, Acros Organics). Dissected brains were incubated in 4% PFA at 4°C for additional 24 hours, after which brains were cryopreserved for 72 hours in 30% sucrose (Fisher) in 1X PBS. Brains were embedded in optimal cutting temperature (O.C.T) compound (Thermo Scientific™ Shandon™ Cryomatrix™) and flash frozen for downstream

assays. For transmission electron microscopy, animals were perfused with HBSS followed by 4% PFA in 0.1M Sodium Cacodylate buffer with 2 mM $CaCl_2$ (solution A). The brain was removed and post-fixed in solution A for 1 hour at RT, followed by 24h incubation at 4°C. Then, 1 mm slices were cut with sagittal mouse brain matrix (RWD), and region of interest dissected into 2.5 mm thickness pieces, which were transferred into 2% PFA, 2.5% Glutaraldehyde, 0.1M Sodium Cacodylate buffer with 2 mM $CaCl_2$. Samples were infiltrated with Spurr's resin and polymerized in 70°C oven with fresh resin for 24 h. Ultrathin 70 nm sections were generated using an ultramicrotome (Leica, EM UC6).

Primary cell cultures:

Microglia cultures: Microglial cells were expanded and isolated through a mixed glial culture as per (Galleguillos et al., 2022; Saura et al., 2003) with slight modifications. Briefly, cerebral cortices from P0-P2 CD1 brains were enzymatically digested and mechanically dissociated and cortical cells were seeded in DMEM/F12 (Dulbecco's Modified Eagle Medium, Ham's F-12, Hyclone) with 10% FBS (Fetal Bovine Serum, Sigma), 1% penicillin/streptomycin (Pen/Strep, Lonza), 1% sodium pyruvate (Invitrogen) and 50 μM β-mercaptoethanol (herein referred to as **microglia media**). Media was replaced twice a week. To increase the number of microglial cells, the culture was treated with 10 ng/ml GM-CSF (Granulocyte Macrophage Colony Stimulating Factor, R&D systems) on days 8-11, which was replaced with microglia media without penicillin/streptomycin the day before microglia isolation. On day 14, conditioned media was collected and filtered and microglial cells were isolated using 15 mM lidocaine (Sigma) and shaking the plate at 100 rpm for 10 min as described in (Galleguillos et al., 2022; Rabinovitch and DeStefano, 1975). Isolated microglial cells were plated at 47,000-52,000 cells/cm^2 density for 24h in 50% conditioned media and 50% microglia media for polarization, assessment of proliferation or for co-culture with OPCs as described below. For proliferation analysis, after 24h media was replaced to microglia media without β-mercaptoethanol and VC (1xPBS) or 250 ng/ml FKN in VC was added for 3 days. For co-cultures, isolated OPCs were plated on top of microglia as described below. For co-cultures with pre-treatment, isolated microglia were plated at 47,000-52,000 cells/cm2 density for 24h in 50% conditioned media and 50% microglia media for 24h, followed by 24h in microglia media without FBS but with VC (1xPBS) or 250ng/ml FKN. VC/FKN pre-treated cells were washed once with microglia media without FBS. Isolated pre-treated OPCs were plated on top of microglia as described below.

Cortical OPC cultures: Cerebral cortices from P0-P2 CD1 brains were dissociated as described above and cultured on plates pre-coated with 40 µg/ml Poly-D-Lysine (Sigma) and 4 µg/ml laminin (Corning) in Serum-Free Media (SFM: DMEM low glucose [Gibco], F12 [Gibco], 0.6% glucose [Sigma], 0.1125% $NaHCO_3$ [Gibco], 5 mM N-2-hydroxyethylpiperazine-N-2-ethane sulfonic acid [HEPES, Gibco], 100 µg/mL L-glutamine [Lonza], 1% Pen/Strep) supplemented with 2% B27 supplement (Life Technologies), 10 ng/ml FGF (Peprotech) and 10 ng/ml PDGF-AA (R&D) (herein referred to **OPC growth media [GM]**). Media was changed every 4 days. After 15 days, cells were lifted with accutase (Life Technologies) and subjected to immunopanning with PDGFRα-specific magnetic beads as per manufacturer's instructions (Miltenyi). Recovered cells were then seeded at 47,000 cells/cm^2 on top of pre-plated microglia or alone in SFM supplemented with 2% B27 and 40 ng/ml 3,3',5-Triiodo-L-thyronine (T3, Sigma) (herein referred to **OPC differentiation media [DM]**). Cells were incubated for an additional 1-3 days. For co-cultures with pre-treatment, OPCs were cultured for 15 days as described above, followed by treatment with VC (1xPBS) or 250 ng/mL FKN for 24h. Cells were then washed, lifted with accutase, isolated via immunopanning, and seeded on top of pre-plated pre-treated microglia and incubated for an additional 3 days in OPC DM as described above.

Microglia polarization: Isolated microglia were treated with polarizing cytokines as described in (Durafourt et al., 2012; Galleguillos et al., 2022; Krasnow et al., 2017). Briefly, microglia were plated on poly-D-lysine and laminin coated coverslips and incubated in 50% conditioned media/50% microglia media for 24h. At this time point, media was changed to microglia medium without FBS or β-mercaptoethanol containing no cytokines (basal conditions), 10 ng/ml IL-1β (Cedarlane) or 20 ng/ml IL-4 (Miltenyi) and IL-13 (R&D Systems). After 24h polarization, conditioned media was collected for ELISA (enzyme-linked immunosorbent assay) as specified below, and replaced with microglia media without FBS or β-mercaptoethanol and containing VC (1xPBS) or 250 ng/ml FKN dissolved in VC. 24h after VC/FKN treatment, cells were processed for downstream assays.

Myelin phagocytosis assays: Myelin labeled with pHRodo was added to microglia cultured in basal conditions at 1:1,000 concentration. VC (1x PBS) or 250 ng/ml FKN (dissolved in VC) was added at the same time as myelin debris. Cultures were incubated for 3h and imaged as described below.

Conditioned medium preparation/ELISA: microglia were cultured in the presence of no cytokines (basal conditions), IL-1β or IL-4 and IL-13 as described above. Conditioned medium was collected and centrifuged at 1000 g for 7 min to remove dead cells and debris. TNF-α protein detection was performed via ELISA kit (Invitrogen) following manufacturer's directions. Measurements were performed via technical duplicates from 3 independent preparations of microglia conditioned medium.

Reagents and Immunostaining:

Immunocytochemistry (ICC): Cell cultures were fixed and stained as described in (Watson et al., 2021). Briefly, adherent cell cultures were fixed with 4% PFA for 10 minutes at room temperature, permeabilized with 0.2% NP-40 (Sigma) for 5 min, and blocked with 6% donkey serum (Jackson ImmunoResearch) in 0.5% BSA in 1X PBS. Primary antibodies were added for overnight at 4°C, and appropriate secondary antibodies were added for 1 hour at room temperature (list of antibodies is in the "Antibodies" section below). Nuclei were counter-stained with Hoechst 33258 (Riodel-De Haen Ag). Slides were mounted with Fluoromount G (ThermoFisher).

Immunohistochemistry (IHC): Cryopreserved brains were sectioned at 18 μm and stained as described in (Watson et al., 2021). For FKN-647 diffusion assay, sections were rehydrated in 1XPBS, immediately counterstained with Hoechst 33258 to visualize nuclei and mounted with Fluoromount G. For all other staining, sections were rehydrated in 1X PBS and then permeabilized and blocked with 5% BSA and 0.3% Triton-X100 in 1X PBS for 1 hour at room temperature. Tissue sections were incubated overnight at 4°C with appropriate primary antibodies listed in "Antibodies" section diluted in 5% BSA in 1X PBS. Mouse on mouse (MOM) kit was used for primary antibodies raised in mouse, in accordance with manufacturer's instructions (VectorLabs). Appropriate secondary antibodies listed in "Antibodies" section were added for 1h at room temperature. Nuclei were counterstained with Hoechst 33258. Sections were mounted with Fluoromount G.

Staining with FluoroMyelin was performed as per manufacturer's instructions (Invitrogen). Briefly, sections were rehydrated in 1X PBS and then permeabilized and blocked with PBS and 0.2% Triton-X100 in 1X PBS for 25 min at room temperature. Stock FluoroMyelin was diluted 300X in 1X PBS and added to sections for 20 min at room temperature. Nuclei were counterstained with Hoechst 33258. Sections were mounted with Fluoromount G.

Antibodies: Mouse anti-APC/CC1 (1:300, Calbiochem, RRID:AB_2057371), sheep anti-BrdU (Abcam, 1:1000, ICC, RRID:AB_302659), rabbit anti-GFAP (Dako, 1:1000, immunostaining, RRID: AB_10013382), rat anti-GFAP (Thermo Fisher, 1;1000, RRID:AB_2532994), chicken anti-eGFP (Abcam, 1:1000, IHC, RRID: AB_300798), goat anti-IBA1 (Novus Bio, 1:300 for IHC, 1:1000 for ICC, RRID:AB_521594), rabbit anti-IBA1 (Wako, 1:1000, RRID: AB_839504); mouse anti-Ki67 (BD Pharmingen, 1:300, ICC, RRID: AB_396287), rat anti-MBP (a. a. 82-87) (Millipore, 1:500, ICC, RRID: AB_94975), rabbit anti-Olig2 (Millipore, 1:1000 for IHC, 1:2000 for ICC, immunostaining, RRID: AB_570666), mouse anti-Olig2 (Millipore, 1:1000 for IHC, 1:500 for ICC, RRID: AB_10807410), goat anti-PDGFRα (R&D Systems, 1:400 for IHC, 1:300 for ICC, RRID: AB_2236897), rat anti-CD16/32 (Tonbo Biosciences, 1:400 for IHC, 1:400 for ICC, RRID:AB_2621487), rabbit anti-DCX (Abcam, 1:1,000 for IHC, RRID:AB_732011). Fluorescently labeled highly cross-absorbed secondary antibodies (Jackson ImmunoResearch) were used at 1:1000 dilution. If MOM kit was used, Cy3-, DTAF-, or Cy5 conjugated streptavidin (Jackson ImmunoResearch) were used at 1:1000 dilution.

RNA scope: Brain cryosections from WT demyelinated mice were subjected to RNA scope as described in (Voronova et al., 2017; Watson et al., 2021) with probes targeting murine *Cx3cr1* and/or *Pdgfrα* mRNA or negative control probe purchased from Advanced Cell Diagnostics according to the manufacturer's instructions. Briefly, 18 μm sections were dehydrated with ethanol and rehydrated with protease (RNA scope kit) diluted 1:5 in 1xPBS for 10 min at 37°C. For RNA scope-IHC combination, sections were rehydrated with protease diluted 1:10 in 1xPBS for 10 min at 37°C to preserve protein epitopes. Protease-treated sections were washed with wash buffer provided by the RNA scope kit and incubated with probes for 2h at 37°C. Sections were then washed again and incubated with signal amplification solutions as per the manufacturer's protocol. At this point, sections were either counter-stained with DAPI (provided in the RNA scope kit) and mounted, or processed for IHC. For latter, RNA scope-processed sections were post-fixed in 4% PFA for 10 min at room temperature. After extensive washes with 1xPBS, sections were permeabilized, blocked and stained with anti-IBA1 antibodies as described in the IHC section.

Microscopy: Brain sections were imaged with Zeiss LSM700 confocal microscope with photomultiplier tube (PMT) with 40X objective, where high magnification digital image acquisition was performed with 2-5x digital Zoom using Zen (Zeiss) (Figs. 1, 2, 3D), or with Olympus IX81 fluorescence microscope equipped with Okogawa CSU X1 spinning disk confocal scan head, 20x objective and a Hamamatsu camera (Hamamatsu), where digital image acquisition was performed with Volocity (Perkin Elmer) software (Fig. S1E). Z-stacks spanning 6-10 μm were taken with optical slice thickness 0.2-0.5 μm and stacked images or orthogonal sections through 3D projections are shown.

Images from all fixed primary cell culture experiments as well as *in vivo* experiments in Figs. 3K, 4-5, 6M-N, S1C-D, S3, S4 were captured with 20X objective using Zeiss Axio Imager M2 fluorescence microscope, ORCA-Flash LT sCMOS Camera and the Zen software (Zeiss). Images were captured in single plane except images in Fig. 6M-N were captured using Z-stacks with optical thickness of 0.5 μm spanning 5-8 μm.

Live microglia cultures were imaged using an inverted Zeiss Axio Observer Z1 microscope equipped with Axiocam 503 Mono camera and 20x objective. Five random pictures per well were taken with transmitted light and red excitation filter.

For electron microscopy, ultrathin 70 nm sections were cut from two blocks per sample and imaged under a transmission electron microscope (JEOL JEM-2100, Gatan Orius camera with Digital micrograph) at 200kV acceleration voltage.

RNA isolation and qRT-PCR: Total RNA from primary microglia was purified using Omega Biotek E.Z.N.A. microelute kit as per manufacturer's instructions. 50-80 ng of purified total RNA was reverse transcribed to generate cDNA using QuantiTect Reverse Transcription Kit (Qiagen) as described in (Voronova et al., 2017). One-fortieth of RT reaction was used as a template for qPCR amplification using the Ssoadvanced SYBR Green kit (Biorad) and the following primers: Cx3cr1 (Forward: CGGCCATCTTAGTGGCGTC and Reverse: GGATGTTGACTTCCGAGTTGC), housekeeping genes Hnrnpab (Forward: AGGACGCGGGAAAAATGTTC and Reverse: CAGTCAACAACCTCTCCAAACT) and β-actin (Forward: GGCTGTATTCCCCTCCATCG and Reverse: CCAGTTGGTAACAATGCCATGT). Data were acquired using Eppendorf Realplex2 (Eppendorf) instrument in technical duplicates and from three biological replicates. Data were normalized to β-actin and Hnrnpab as recommended in (Tanaka et al., 2017) and analyzed using $2^{-\Delta\Delta Ct}$ method as in (Livak and Schmittgen, 2001). Basal PBS treated microglia samples were used as a calibrator (equated to 1). All primers were validated, and qPCR assays were performed in accordance with Minimum Information for Publication of Quantitative Real- Time PCR Experiments guidelines (Bustin et al., 2009).

QUANTIFICATION, CO-LOCALIZATION AND STATISTICAL ANALYSIS

Cell cultures were analyzed with a Zeiss Axioimager fluorescence microscope. Digital image acquisition was performed with ZEN (Zeiss) software. In all culture experiments, 5-10 fields of view were captured with a 20X objective. At least 250 cells from each treatment and biological experiment were counted. Proliferation index is presented as %Ki67-positive, marker-positive cells over total marker-positive cells. *In vitro* data (ICC and q-PCR) are presented from at least 3 independent biological experiments.

For all *in vivo* experiments, midline corpus callosum or cortical grey matter in medial sections was imaged using 20X objective as indicated in figures, with exception of Fig. 3E-F, I-J, where rostral and caudal corpus callosum was imaged. Areas of interest were identified with Hoechst staining. For PDGFRαCre^{ERT2};RosaYFP^{STOP} mice, the results are presented as percentage of marker-positive, YFP-positive cells relative to total YFP-positive cells. For other quantifications, average # marker+ cells per field of view or defined cortical column is presented. Proliferative index is presented as % marker+BrdU+/marker+ cells. 5 matched sections per brain were analyzed from 6 mice across 3 independent litters. At least 300 cells per corpus callosum and 800 cells per cortical column in every sample were counted. *In vivo* analysis was performed in a blind fashion by two independent observers.

For co-localization analysis, cortical grey matter was imaged using 20X objective and captured using Z-stacks with an optical thickness of 0.5 μm spanning 5-8 μm. At least 4 anatomically matched images per mouse, from 3 mice per treatment group from at least two independent litters were analyzed. Z-stacks were then subjected to Imaris software (Imaris64 9.3.0) and voxel-based MBP/mGFP co-localization analysis was performed. Colocalization was calculated within the region of interest (ROI) and determined by the intensity threshold. % of ROI co-localized was normalized to VC.

Electron microscopy images were analyzed for g-ratio and morphometric parameters as described in (Bando et al., 2015; Edgar et al., 2010; Edgar et al., 2009; Edgar et al., 2020). In brief, the g-ratio was measured for myelinated axons, where axon and compact myelin morphology were normal as described in (Bando et al., 2015). The following axons were excluded from g-ratio analysis: axons with abnormal myelin morphology (e.g. decompacted, redundant and splitting myelin, see Fig. S5A) and axons that showed signs of degradation or swelling (see Fig. S5B) (Bando et al., 2015). On average, at least 8 images and 100 axons per sample were analyzed. Axonal and myelin abnormalities were separately measured using

morphometric analysis as described in (Edgar et al., 2020; Schäffner et al., 2021). Briefly, relative proportion of normal and abnormal myelin was determined by manually counting the number of intersections of normal and abnormal myelin with 64 equidistant grids overlaid in Fiji software. To determine the proportion of degenerating (e.g. axons with darkened axoplasm or swollen axons with organelle accumulation) or myelinated/unmyelinated axons, axons identified based on microtubules and neurofilaments were counted within a region of interest (ROI) superimposed onto the micrographs. Axonal density was analyzed using axons that fit within the ROI is presented as number of total or myelinated axons/mm^2. Proportion of degenerating or myelinated axons is presented as % of total axons or as total axons binned by axonal diameter. For axonal density and morphometric analyses, at least 30 images per sample were analyzed.

Images were counted in Zen or Fiji software (Schindelin et al., 2012) in a blinded fashion. Representative images were processed in Photoshop CC 2015 and figures in Adobe Illustrator CC 2015. Biorender was used to generate brain schematics.

Sample sizes (n) indicated in the figure legends 2-7 correspond to the number of biological replicates analyzed. Data in Fig. 1 and S1 is presented from two mice per treatment. All data are presented as mean ± SEM or as connected lines, where each line corresponds to one biological replicate.

All data were subjected to normality tests with D'Agostino & Pearson, Shapiro-Wilk and Kolmogorov-Smirnov tests and were considered normal. For two group comparisons, two-tailed paired student's t-tests (*in vitro* datasets) or two-tailed unpaired student's t-tests (for *in vivo* datasets) or multiple t-tests were used to assess statistical significance between means, where a p-value <0.05 was considered significant. For three or more group comparisons one-way or two-way ANOVA followed by Dunnett's or Tukey multiple comparisons tests were used. For comparing slopes of g-ratios with axon diameters, simple linear regression was applied where 95% CI of the best-fit line was used. In all cases, Prism (version 8.0.2) was used. Number of experiments and statistical information are stated in the corresponding figure legends. In figures, asterisks denote statistical significance marked by *, $p < 0.05$; **, $p < 0.01$; ***, $p < 0.001$.

References:

Bando, Y., Nomura, T., Bochimoto, H., Murakami, K., Tanaka, T., Watanabe, T., and Yoshida, S. (2015). Abnormal morphology of myelin and axon pathology in murine models of multiple sclerosis. Neurochemistry international 81, 16-27, https://doi.org/10.1016/j.neuint.2015.01.002.

Bhattacherjee, A., Rodrigues, E., Jung, J., Luzentales-Simpson, M., Enterina, J.R., Galleguillos, D., St Laurent, C.D., Nakhaei-Nejad, M., Fuchsberger, F.F., Streith, L., *et al.* (2019). Repression of phagocytosis by human CD33 is not conserved with mouse CD33. Communications biology 2, 450, https://doi.org/10.1038/s42003-019-0698-6.

Bustin, S.A., Benes, V., Garson, J.A., Hellemans, J., Huggett, J., Kubista, M., Mueller, R., Nolan, T., Pfaffl, M.W., Shipley, G.L., *et al.* (2009). The MIQE guidelines: minimum information for publication of quantitative real-time PCR experiments. Clin Chem 55, 611-622, https://doi.org/10.1373/clinchem.2008.112797.

Durafourt, B.A., Moore, C.S., Zammit, D.A., Johnson, T.A., Zaguia, F., Guiot, M.C., Bar-Or, A., and Antel, J.P. (2012). Comparison of polarization properties of human adult microglia and blood-derived macrophages. Glia 60, 717-727, https://doi.org/10.1002/glia.22298.

Edgar, J.M., McCulloch, M.C., Montague, P., Brown, A.M., Thilemann, S., Pratola, L., Gruenenfelder, F.I., Griffiths, I.R., and Nave, K.A. (2010). Demyelination and axonal

preservation in a transgenic mouse model of Pelizaeus-Merzbacher disease. EMBO Mol Med 2, 42-50, https://doi.org/10.1002/emmm.200900057.

Edgar, J.M., McLaughlin, M., Werner, H.B., McCulloch, M.C., Barrie, J.A., Brown, A., Faichney, A.B., Snaidero, N., Nave, K.A., and Griffiths, I.R. (2009). Early ultrastructural defects of axons and axon-glia junctions in mice lacking expression of Cnp1. Glia 57, 1815-1824, https://doi.org/10.1002/glia.20893.

Edgar, J.M., Smith, R.S., and Duncan, I.D. (2020). Transmission Electron Microscopy and Morphometry of the CNS White Matter. Methods Mol Biol 2143, 233-261, https://doi.org/10.1007/978-1-0716-0585-1_18.

Galleguillos, D., Wang, Q., Steinberg, N., Zaidi, A., Shrivastava, G., Dhami, K., Daskhan, G.C., Schmidt, E.N., Dworsky-Fried, Z., Giuliani, F., *et al.* (2022). Anti-inflammatory role of GM1 and other gangliosides on microglia. J Neuroinflammation 19, 9, https://doi.org/10.1186/s12974-021-02374-x.

Jäkel, S., Agirre, E., Mendanha Falcão, A., van Bruggen, D., Lee, K.W., Knuesel, I., Malhotra, D., ffrench-Constant, C., Williams, A., and Castelo-Branco, G. (2019). Altered human oligodendrocyte heterogeneity in multiple sclerosis. Nature 566, 543-547, https://doi.org/10.1038/s41586-019-0903-2.

Kang, S.H., Fukaya, M., Yang, J.K., Rothstein, J.D., and Bergles, D.E. (2010). NG2+ CNS glial progenitors remain committed to the oligodendrocyte lineage in postnatal life and following neurodegeneration. Neuron 68, 668-681, https://doi.org/10.1016/j.neuron.2010.09.009.

Kosaraju, J., Seegobin, M., Gouveia, A., Syal, C., Sarma, S.N., Lu, K.J., Ilin, J., He, L., Wondisford, F.E., Lagace, D., *et al.* (2020). Metformin promotes CNS remyelination and improves social interaction following focal demyelination through CBP Ser436 phosphorylation. Exp Neurol 334, 113454, https://doi.org/10.1016/j.expneurol.2020.113454.

Krasnow, S.M., Knoll, J.G., Verghese, S.C., Levasseur, P.R., and Marks, D.L. (2017). Amplification and propagation of interleukin-1β signaling by murine brain endothelial and glial cells. J Neuroinflammation 14, 133, https://doi.org/10.1186/s12974-017-0908-4.

Livak, K.J., and Schmittgen, T.D. (2001). Analysis of relative gene expression data using real-time quantitative PCR and the 2(-Delta Delta C(T)) Method. Methods 25, 402-408, https://doi.org/10.1006/meth.2001.1262.

Muzumdar, M.D., Tasic, B., Miyamichi, K., Li, L., and Luo, L. (2007). A global double-fluorescent Cre reporter mouse. Genesis 45, 593-605, https://doi.org/10.1002/dvg.20335.

Rabinovitch, M., and DeStefano, M.J. (1975). Use of the local anesthetic lidocaine for cell harvesting and subcultivation. In vitro 11, 379-381, https://doi.org/10.1007/bf02616374.

Saito, L.B., Fernandes, J.P., Smith, M.J., Doan, M.A.L., Branton, W.G., Schmitt, L.M., Wuest, M., Monaco, M.C., Major, E.O., Wuest, F., *et al.* (2021). Intranasal anti-caspase-1 therapy preserves myelin and glucose metabolism in a model of progressive multiple sclerosis. Glia 69, 216-229, https://doi.org/10.1002/glia.23896.

Saura, J., Tusell, J.M., and Serratosa, J. (2003). High-yield isolation of murine microglia by mild trypsinization. Glia 44, 183-189, https://doi.org/10.1002/glia.10274.

Schäffner, E., Edgar, J., Lehning, M., Strauß, J., Bosch-Queralt, M., Wieghofer, P., Berghoff, S., Krueger, M., Morawski, M., Reinert, T., *et al.* (2021). Myelin insulation as a risk factor for axonal degeneration in autoimmune demyelinating disease. bioRxiv, 2021.2011.2011.468223, https://doi.org/10.1101/2021.11.11.468223.

Schindelin, J., Arganda-Carreras, I., Frise, E., Kaynig, V., Longair, M., Pietzsch, T., Preibisch, S., Rueden, C., Saalfeld, S., Schmid, B., *et al.* (2012). Fiji: an open-source platform for biological-image analysis. Nature Methods 9, 676-682, https://doi.org/10.1038/nmeth.2019.

Srinivas, S., Watanabe, T., Lin, C.S., William, C.M., Tanabe, Y., Jessell, T.M., and Costantini, F. (2001). Cre reporter strains produced by targeted insertion of EYFP and ECFP into the ROSA26 locus. BMC Dev Biol 1, 4, https://doi.org/10.1186/1471-213x-1-4.

Tanaka, A., To, J., O'Brien, B., Donnelly, S., and Lund, M. (2017). Selection of reliable reference genes for the normalisation of gene expression levels following time course LPS stimulation of murine bone marrow derived macrophages. BMC immunology 18, 43, https://doi.org/10.1186/s12865-017-0223-y.

Voronova, A., Yuzwa, S.A., Wang, B.S., Zahr, S., Syal, C., Wang, J., Kaplan, D.R., and Miller, F.D. (2017). Migrating Interneurons Secrete Fractalkine to Promote Oligodendrocyte Formation in the Developing Mammalian Brain. Neuron 94, 500-516.e509, https://doi.org/10.1016/j.neuron.2017.04.018.

Watson, A.E.S., de Almeida, M.M.A., Dittmann, N.L., Li, Y., Torabi, P., Footz, T., Vetere, G., Galleguillos, D., Sipione, S., Cardona, A.E., *et al.* (2021). Fractalkine signaling regulates oligodendroglial cell genesis from SVZ precursor cells. Stem Cell Reports 16, 1968-1984, https://doi.org/10.1016/j.stemcr.2021.06.010.
